# Effect of acupuncture for temporomandibular disorders: a randomized clinical trial

**DOI:** 10.1093/qjmed/hcae094

**Published:** 2024-05-06

**Authors:** Lu Liu, Qiuyi Chen, Tianli Lyu, Luopeng Zhao, Quan Miao, Yuhan Liu, Limin Nie, Feiyu Fu, Shuting Li, Chenxi Zeng, Yixin Zhang, Peiyue Peng, Woyu Wang, Ying Lin, Bin Li

**Affiliations:** Department of Acupuncture and Moxibustion, Beijing Hospital of Traditional Chinese Medicine, Capital Medical University, Beijing Key Laboratory of Acupuncture Neuromodulation, Beijing 100010, China; Department of Acupuncture and Moxibustion, Beijing Hospital of Traditional Chinese Medicine, Capital Medical University, Beijing Key Laboratory of Acupuncture Neuromodulation, Beijing 100010, China; Department of Acupuncture and Moxibustion, Beijing Hospital of Traditional Chinese Medicine, Capital Medical University, Beijing Key Laboratory of Acupuncture Neuromodulation, Beijing 100010, China; Department of Acupuncture and Moxibustion, Beijing Hospital of Traditional Chinese Medicine, Capital Medical University, Beijing Key Laboratory of Acupuncture Neuromodulation, Beijing 100010, China; Department of Acupuncture and Moxibustion, Beijing Hospital of Traditional Chinese Medicine, Capital Medical University, Beijing Key Laboratory of Acupuncture Neuromodulation, Beijing 100010, China; Department of Acupuncture and Moxibustion, Beijing Hospital of Traditional Chinese Medicine, Capital Medical University, Beijing Key Laboratory of Acupuncture Neuromodulation, Beijing 100010, China; Department of Acupuncture and Moxibustion, Beijing Hospital of Traditional Chinese Medicine, Capital Medical University, Beijing Key Laboratory of Acupuncture Neuromodulation, Beijing 100010, China; Department of Acupuncture and Moxibustion, Beijing Hospital of Traditional Chinese Medicine, Capital Medical University, Beijing Key Laboratory of Acupuncture Neuromodulation, Beijing 100010, China; Department of Acupuncture and Moxibustion, Beijing Hospital of Traditional Chinese Medicine, Capital Medical University, Beijing Key Laboratory of Acupuncture Neuromodulation, Beijing 100010, China; Department of Acupuncture and Moxibustion, Beijing Hospital of Traditional Chinese Medicine, Capital Medical University, Beijing Key Laboratory of Acupuncture Neuromodulation, Beijing 100010, China; Department of Acupuncture and Moxibustion, Beijing Hospital of Traditional Chinese Medicine, Capital Medical University, Beijing Key Laboratory of Acupuncture Neuromodulation, Beijing 100010, China; Department of Acupuncture and Moxibustion, Beijing Hospital of Traditional Chinese Medicine, Capital Medical University, Beijing Key Laboratory of Acupuncture Neuromodulation, Beijing 100010, China; Department of Acupuncture and Moxibustion, Beijing Hospital of Traditional Chinese Medicine, Capital Medical University, Beijing Key Laboratory of Acupuncture Neuromodulation, Beijing 100010, China; Department of Acupuncture and Moxibustion, Beijing Hospital of Traditional Chinese Medicine, Capital Medical University, Beijing Key Laboratory of Acupuncture Neuromodulation, Beijing 100010, China; Department of Acupuncture and Moxibustion, Beijing Hospital of Traditional Chinese Medicine, Capital Medical University, Beijing Key Laboratory of Acupuncture Neuromodulation, Beijing 100010, China

## Abstract

**Background:**

Temporomandibular disorders (TMD) are the leading cause of pain and disability among frequently occurring facial pain and the second leading cause of musculoskeletal conditions.

**Aim:**

We examined whether acupuncture could alleviate pain intensity in patients with TMD.

**Design and methods:**

Sixty participants with TMD were randomly assigned (ratio 1:1) to receive three acupuncture or sham acupuncture sessions weekly for 4 weeks. The primary outcome was the change in the mean weekly pain intensity from baseline to week 4. Secondary and exploratory outcomes included proportion of participants with ≥30% or ≥50% reduction in pain intensity, change in jaw opening and movement, graded chronic pain scale, jaw functional limitations scale-20-item, depression, anxiety and stress scales-21, Pittsburgh sleep quality index at week 4 and 8, and the pressure pain threshold and surface electromyography at week 4.

**Results and conclusion:**

The acupuncture group showed significantly reduced pain intensity compared to the sham group at week 4 (−1.49, 95% confidence interval [CI]: −2.32 to −0.65; *P* < 0.001) and week 8 (−1.23, 95% CI: −2.11 to −0.54; *P* = 0.001). Acupuncture’s effectiveness surpassed sham’s at 4 weeks and lasted 8 weeks. Participants in the acupuncture group experienced significantly greater improvements in the 30% and 50% response rate, jaw opening and movement, GCPS, JFLS-20, DASS-21 and PSQI than those in the sham acupuncture group. There were no significant between-group differences in PPT and sEMG. In summary, acupuncture provided marked pain relief and improvement in physical and emotional function for patients with TMD compared with sham acupuncture.

## Introduction

Temporomandibular disorders (TMD) involve symptoms from the temporomandibular joint (TMJ), mastication muscles and their associated structures,^1^ affecting 11–33% of the population, mainly younger individuals.^2^^,^^3^ It is the leading cause of pain and disability among frequently occurring facial pain and the second leading cause of musculoskeletal conditions, costing an estimated $4 billion annually.^4^ The recent diagnostic criteria classify TMD (DC/TMD) into two groups, one of which is pain-related disorders (including myalgia, arthralgia and TMD-related headaches).^5^ Facial pain, the primary symptom prompting treatment, averages a TMD pain intensity of 5.0 on an 11-point scale.^6^ Patients with TMD often report comorbid headaches, depression, sleep disorders and fibromyalgia.^7–9^

The primary goals in managing TMD are to reduce pain, enhance TMJ function and alleviate masticatory muscle spasms.^10^ Common treatments include conservative treatment (pharmacotherapy and non-pharmacotherapy), minimally invasive surgical procedures and invasive surgical procedures.^11–13^ A Cochrane review revealed a lack of randomized controlled trials for TMD pharmacotherapy, leading to mostly empirical treatment.^14^ While several reviews have evaluated its pain relief efficacy, few have examined improvements in joint function.^14^^,^^15^ Non-pharmacotherapy encompasses occlusal splint therapy, acupuncture, cognitive behavioral therapy, physiotherapy and education.^16^ Evidence-based non-pharmacotherapy should be the first-line treatment in patients with TMD due to their low risk of side effects and reversibility.^1^^,^^16^

Recommended by the World Health Organization for pain management,^17^ acupuncture is a recognized non-pharmacotherapy option for TMD treatment.^18^ Systematic reviews indicated acupuncture effectively alleviates pain and masseter muscle tenderness in patients with TMD.^19^^,^^20^ However, several trials observed no differences between real and sham acupuncture.^21^^,^^22^ These inconsistencies could stem from variations in placebo controls and study designs. An ideal placebo acupuncture design should be physiologically inert yet indistinguishable from real acupuncture. The “Park Sham Device” has been validated for effectively blinding participants while simulating a real acupuncture therapeutic setting.^23^

Given the urgent need for well-designed randomized controlled trials with standardized, patient-based outcomes for TMD,^24^ we conducted a randomized, sham-controlled, patient-blinded clinical trial to assess whether a 4-week real acupuncture course, compared to sham acupuncture, alleviates pain intensity and joint functional disability in patients with TMD.

## Methods

### Study design

This single-center, single-blind, randomized controlled trial was conducted in China. The protocol and statistical analysis plan are presented in Supplement 1. The study comprised a 1-week screening, 1-week baseline, 4-week treatment and 4-week follow-up period (Figure S1 of Supplement 2). All participants provided written informed consent.

This study was approved by the Research Ethics Committee of Beijing Hospital of Traditional Chinese Medicine, Capital Medical University (2018BL-060-01) and was conducted in accordance with the International Conference on Harmonization-Good Clinical Practice and Declaration of Helsinki.^25^ The protocol was registered (clinicaltrials.gov NCT04210921).

### Participants

Eligible participants were males and females aged 18–80 with a minimum three-month history of pain-related TMD, confirmed by the Diagnostic Criteria of TMD (DC/TMD) (Supplement 2 pages 5–7),^5^ and were verified by an examiner. Exclusion criteria were intra-articular TMD diagnosis; had received TMD-specific medication, acupuncture, occlusal splint or other concomitant therapy for managing facial pain within one month before the screening period; had other medical conditions or were pregnant or lactating. Detailed criteria are in Supplement 2 (pages 3–4).

### Randomization and blinding

Participants were randomized (1:1) to the acupuncture or the sham acupuncture group using an interactive web-based system with a computer-generated code and block size of four, stratified by baseline TMD-specific medication exposure. An independent statistician generated the randomization sequence without further involvement in the enrollment, treatment or assessment. Participants, statistical analysts and outcome assessors were blinded to the groups.

### Interventions

Needling was performed by licensed acupuncturists with ≥5 years of experience. Patients underwent unified and standardized training before trial initiation. Patients received a 30-min treatment thrice weekly for 4 weeks. Treatments occurred in private rooms to prevent communication, and standardized procedures ensured consistent rituals in both the groups (Supplement 2, pages 9–10).

In the acupuncture group, prescriptions were based on information from classic and modern literature^19^^,^^26^^,^^27^ and previous studies.^18^ Acupuncturists performed the treatment at acupoints as follows: bilateral LI4 and GB34; affected-side SI19, ST6 and ST7. The acupoint details are presented in Table S1 and Figure S2 of Supplement 2. Participants were placed in the supine position, and the skin was sterilized. Disposable, single-use, real stainless-steel needles (DONGBANG Acupuncture Inc., Korea) were inserted using a normal guide device secured on the skin using a self-adhesive pad (Figure S3 of Supplement 2). Needles of 0.35 mm diameter and 70 mm length were used, with twirling, lifting and thrusting to induce deqi (a characteristic sensation of soreness, numbness, distention or heaviness that indicates effective needling). Details of acupoints, manipulation of the acupoints and acupuncture device are presented in Table S2 and Figure S2 of Supplement 2.

Patients in the sham acupuncture group received noninvasive acupuncture at the same acupoints as those in the acupuncture group. Sham needles, similar to real ones but blunt and sliding within their handles, were applied using a Park Sham device secured on the skin using a self-adhesive pad (Figure S3 of Supplement 2). The manipulation rituals were the same as those for acupuncture but without deqi. Differences and similarities between both the treatments are summarized in Table S2 of Supplement 2.

Participants documented pain-related information using a TMD diary, including the following characteristics: pain severity, pain duration and acute pain medication use. They were allowed to take acute pain medications (NSAIDs, acetaminophen or aspirin) prescribed by our dentist. We required the same acute pain medication to be used throughout the treatment. TMD-specific medications (see page 11 of Supplement 1) were not allowed throughout the study.

### Outcomes

The primary outcome was the change from baseline in mean weekly pain intensity (measured by VAS) at week 4. Secondary outcomes were the proportion of participants with ≥30% or ≥50% reduction in mean weekly pain intensity, change from baseline in jaw opening and movement (pain-free jaw opening, maximum unassisted jaw opening, maximum assisted jaw opening, protrusion movement, left lateral movement and right lateral movement), graded chronic pain scale (GCPS), jaw functional limitations scale-20-item (JFLS-20), depression, anxiety and stress scales-21 (DASS-21) and the Pittsburgh sleep quality index (PSQI) at week 4. Additional secondary outcomes included the mean change from baseline in pressure pain threshold (PPT) and surface electromyography (sEMG) at week 4, with measurement sites detailed in Supplement 2 (Figures S4 and S5). These outcomes were also assessed at week 8 as exploratory outcomes.

Safety outcomes included adverse events (AEs), abnormal laboratory tests, vital signs and weight. Before treatment, participants’ expectations were assessed using an acupuncture expectancy scale. The participants’ satisfaction and blinding were assessed at the end of the treatment period. Compliance was determined by the proportion completing at least 10 of 12 sessions (≥80% compliance rate). Study procedures and assessments are summarized in Table S3 of Supplement 2.

### Statistical analyses

Based on our pilot study, the reduced pain intensity of patients with TMD after the 4-week treatment was 3.4 ± 0.3 and 2.9 ± 0.7 in the acupuncture and sham acupuncture groups, respectively. We calculated that 50 patients would provide ≥90% study power at a two-sided alpha of 0.05. Accounting for a 10% dropout rate, we aimed for 30 patients per group.

The intention-to-treat (ITT) set included all randomly assigned participants. The per-protocol (PP) set, a subset of the ITT set, contained participants completing the study without major protocol deviations or omissions. The safety set consisted of randomly assigned participants who received at least one treatment. The study’s outcomes were analyzed based on the ITT and PP sets. All safety-related analyses were performed using the safety set.

The weekly pain intensity was calculated if a participant completed at least four of the seven daily reports of pain intensity. For patients with missing days and fewer than 4 days of TMD diary data for 1 week, the weekly pain intensity was considered missing before the multiple imputation procedure (see page 88 in Supplement 1). The statistics were based on 10 sets of imputed data, where the mean is the average of the means from the 10 data sets and the standard error (SE) of the mean was adjusted based on the imputation variance estimates.

The primary outcome, the change from baseline in the mean weekly pain intensity at week 4, was performed using a linear regression model adjusted for sex, age, weekly pain intensity at baseline and TMD-specific medication. The least-squares mean (LSM) with SE for the treatment group, LSM, 95% confidence intervals (CIs) for treatment difference and associated *P*-values are provided. Continuous secondary and exploratory outcomes were analyzed similarly to the primary outcome. For the proportion of ≥30% or ≥50% reduction in mean weekly pain intensity, a logistic regression model was used, which was adjusted for sex, age, weekly pain intensity at baseline and TMD-specific medication. Mean percentages with SE and odds ratios (ORs) with 95% CI were presented. AEs were summarized by participant counts and percentages in both the groups, compared using Fisher’s exact test. Blinding effectiveness was assessed by the distribution of participants who thought they received acupuncture, thought they received sham acupuncture or did not know which, using *χ*^2^ test and Bang’s blinding index.^28^ We conducted a PP analysis as sensitivity analyses.

All statistical tests were two-sided with a significance level of 0.05, without adjustments for multiple comparisons and performed using R software (version 4.0.3, R Core Team, Vienna, Austria).

## Results

### Study participants

**Figure 1.**
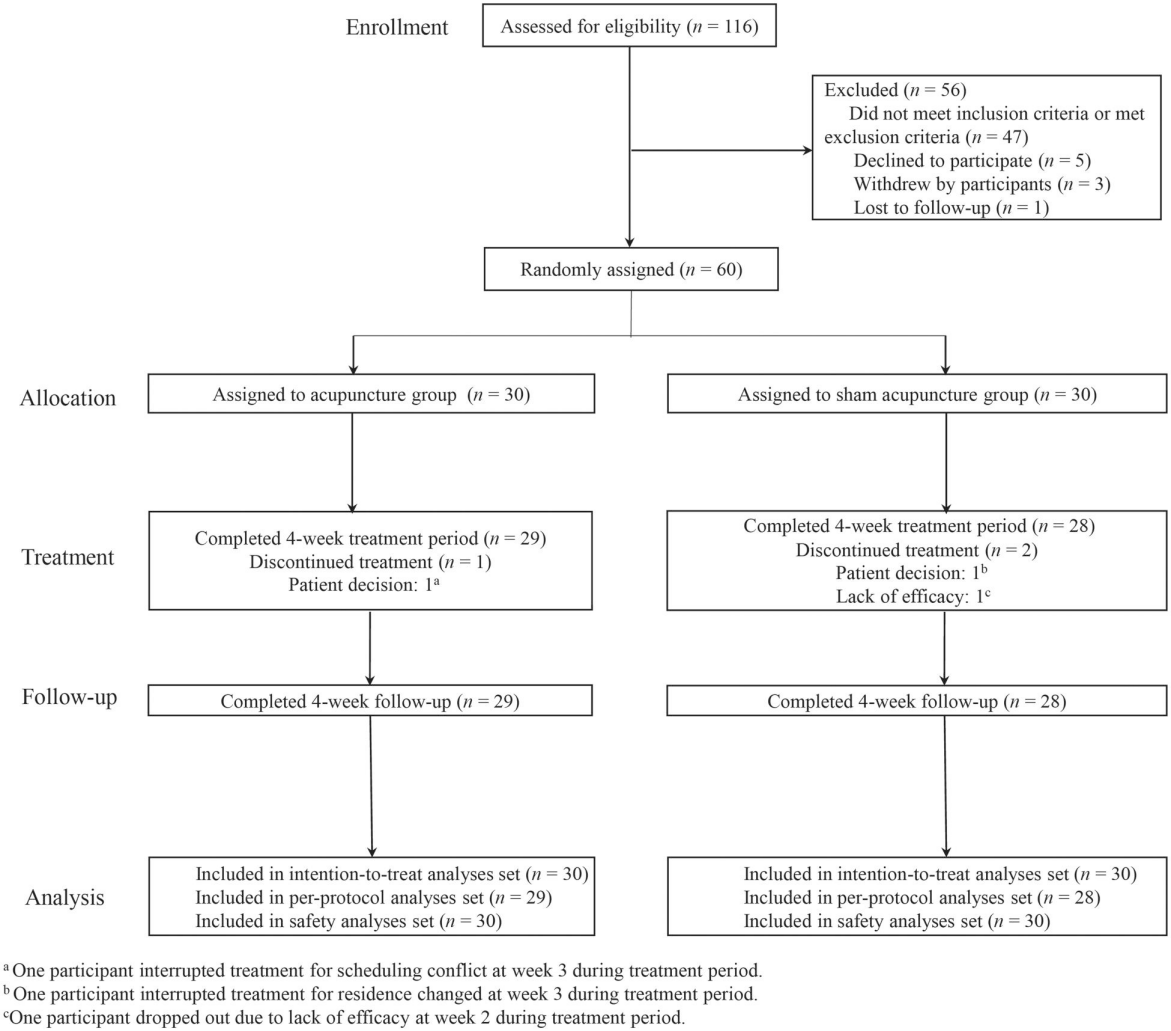
Screening, randomization, intervention and follow-up of participants through study.

After screening 116 individuals for eligibility between 1 April 2019 and 23 July 2022, 56 participants were excluded. The main reasons for screen failure were not meeting the inclusion criteria (Figure 1; Table S4 of Supplement 2). Thus, the ITT population comprised 60 patients (Table 1), and 57 patients completed the treatment plan in the PP population (Table S5 of Supplement 2). They were comparable across the two groups (Table 1). Most participants were females (88.3%) with a mean age of 44.4 (16.7) years.

**Table 1.** Demographic and baseline clinical characteristics in the intention-to-treat population

Characteristics	Acupuncture group (*n* = 30)	Sham acupuncture group (*n* = 30)
Demographics		
Age, mean (SD), years	42.3 (15.6)	46.4 (17.3)
Sex, no. (%)		
Female	26 (86.7)	27 (90.0)
Male	4 (13.3)	3 (10.0)
Body-mass index, kg/m^2^	22.1 (3.2)	22.0 (2.5)
Current employment status		
Employed	19 (63.3)	20 (66.7)
Student	5 (16.7)	3 (10.0)
Unemployed, retired	6 (20.0)	7 (23.3)
Education		
≤High school	6 (20.0)	4 (13.3)
College	20 (66.7)	21 (70.0)
Graduate degree	4 (13.3)	5 (16.7)
Marital status		
Never married	9 (30.0)	10 (33.3)
Married	19 (63.3)	19 (63.3)
Divorced	2 (6.7)	1 (3.3)
Annual income (CNY)		
¥100 000 or less	6 (20.0)	13 (43.3)
¥100 000–¥200 000	11 (36.7)	8 (26.7)
¥200 000–¥500 000	12 (40.0)	9 (30.0)
>¥500 000	1 (3.3)	0 (0.0)
Disease characteristics during the baseline		
Facial pain		
Time since onset, years	2.1 (2.8)	2.0 (2.7)
Weekly pain intensity, mean (SD)	5.3 (1.8)	5.8 (2.0)
Painful days in the last 30 days	16.7 (11.6)	18.9 (11.3)
TMD-specific medication, *n* (%)	6 (20.0)	7 (23.3)
DC/TMD examination findings		
TMD myalgia, *n* (%)	23 (76.7)	23 (76.7)
TMD arthralgia, *n* (%)	28 (93.3)	26 (86.7)
TMD headache, *n* (%)	7 (23.3)	6 (20.0)
Jaw opening and movement		
Pain-free jaw opening, mm	22.0 (9.9)	24.6 (8.8)
Maximum unassisted jaw opening, mm	28.6 (9.1)	32.2 (6.8)
Maximum assisted jaw opening, mm	32.0 (9.1)	35.8 (5.9)
Protrusion movement, mm	3.2 (2.1)	3.8 (1.9)
Left lateral movement, mm	5.3 (2.5)	5.8 (1.9)
Right lateral movement, mm	5.2 (2.4)	6.2 (2.6)
Graded chronic pain scale		
Grades I–IIa, *n* (%)	9 (30.0)	10 (33.3)
Grades IIb–IV, *n* (%)	21 (70.0)	20 (66.7)
Characteristic pain intensity, 0–100 scale	56.1 (21.2)	58.5 (16.9)
Disability score, 0–100 scale	34.3 (26.1)	35.4 (26.9)
Jaw functional limitations scale-20 (JFLS-20)		
Mastication, 0–10 scale	5.0 (3.4)	5.3 (3.6)
Vertical jaw mobility, 0–10 scale	4.3 (3.5)	5.2 (3.2)
Verbal and emotional expression, 0–10 scale	2.8 (4.1)	3.1 (3.8)
Overall, 0–10 scale	4.0 (3.4)	4.6 (3.4)
Depression, anxiety and stress scales (DASS-21)		
Depression	4.6 (2.6)	4.9 (2.4)
Anxiety	3.9 (2.9)	4.8 (3.0)
Stress	5.1 (3.6)	5.1 (3.9)
Overall	13.6 (7.2)	14.9 (7.9)
Pittsburgh sleep quality index (PSQI)		
Subjective sleep quality	1.2 (0.8)	1.3 (0.8)
Sleep latency	1.3 (1.2)	1.5 (0.9)
Sleep duration	1.1 (0.9)	1.2 (0.9)
Habitual sleep efficiency	0.7 (1.0)	0.8 (1.0)
Sleep disturbances	1.2 (0.6)	1.5 (0.8)
Use of sleep medication	0.3 (0.8)	0.7 (1.1)
Daytime dysfunction	1.5 (1.0)	1.8 (1.2)
Overall	7.2 (3.7)	8.7 (4.2)
Quantitative sensory testing		
Pressure pain thresholds (PPTs)		
Masseter PPT, 0–500 kPa	124.4 (48.3)	128.9 (42.7)
Anterior temporalis PPT, 0–500 kPa	138.9 (61.7)	142.4 (43.3)
Sternocleidomastoid PPT, 0–500 kPa	114.0 (40.3)	111.8 (36.8)
Trapezius PPT, 0–500 kPa	161.9 (65.4)	176.9 (61.1)
TMJ PPT, 0–500 kPa	119.3 (43.9)	126.9 (42.0)
sEMG		
Mandibular resting position (MR)		
RMS, masseter muscle, left, μV	10.2 (15.7)	14.6 (27.7)
RMS, masseter muscle, right, μV	13.5 (19.6)	15.2 (23.0)
RMS, anterior temporalis muscle, left, μV	15.2 (14.1)	17.6 (24.2)
RMS, anterior temporalis muscle, right, μV	8.6 (5.6)	11.4 (11.2)
Habitual chewing (HC)		
RMS, masseter muscle, left, μV	95.2 (91.5)	64.9 (45.4)
RMS, masseter muscle, right, μV	98.6 (116.8)	69.1 (48.6)
RMS, anterior temporalis muscle, left, μV	103.8 (67.5)	77.4 (58.6)
RMS, anterior temporalis muscle, right, μV	106.0 (62.9)	81.3 (47.5)
Maximal voluntary contraction (MVC)		
RMS, masseter muscle, left, μV	68.0 (45.7)	59.1 (42.2)
RMS, masseter muscle, right, μV	77.1 (77.0)	54.2 (27.5)
RMS, anterior temporalis muscle, left, μV	57.2 (35.6)	52.9 (37.5)
RMS, anterior temporalis muscle, right, μV	58.3 (34.4)	57.1 (31.1)

Data are mean (SD) or *n* (%). SD, standard deviation; CNY, China Yuan; DC/TMD, diagnostic criteria for temporomandibular disorder; GCPS, graded chronic pain scale; JFLS, jaw functional limitation scale; DASS, depression, anxiety and stress scales; PSQI, Pittsburgh sleep quality index; PPT, pressure pain threshold; TMJ, temporomandibular joint; sEMG, surface electromyography; RMS, root mean square; MR, mandibular resting position; HC, habitual chewing; MVC, maximal voluntary contraction.

### Efficacy outcomes

#### Pain-related index

**Figure 2.**
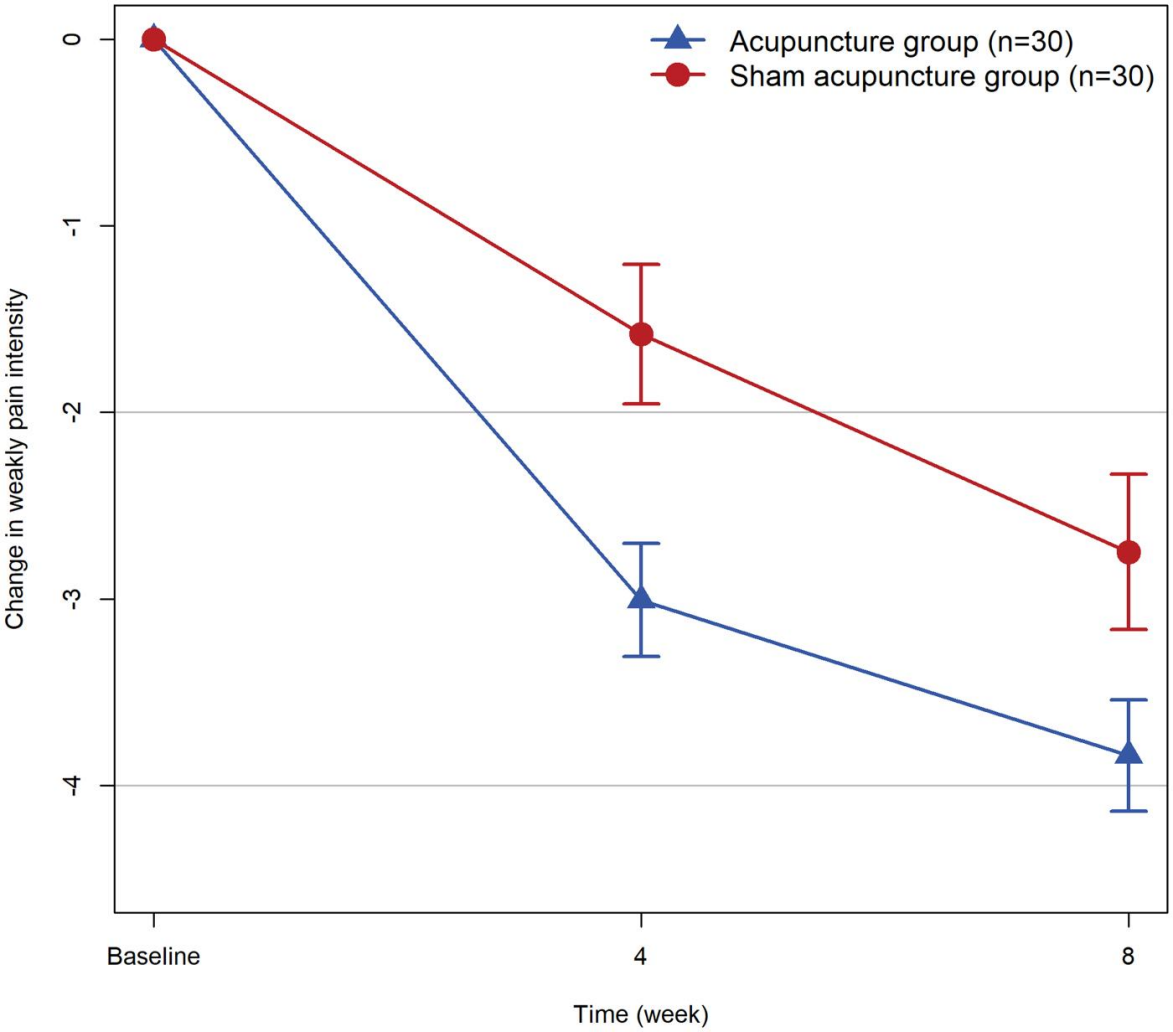
Change from baseline in weekly pain intensity.

The acupuncture group had significantly greater decreases in the mean change from baseline in weekly pain intensity at week 4 than the sham acupuncture group (LSM difference: −1.49 [95% CI: −2.32 to −0.65]; *P* < 0.001; Table 2 and Figure 2), with similar results in the PP analysis (Table S6 of Supplement 2). These reductions were maintained at week 8 (LSM difference: −1.33 [95% CI: −2.11 to −0.54]; *P* = 0.001).

**Table 2.** Primary, secondary and exploratory clinical efficacy outcomes during the treatment and follow-up periods in the intention-to-treat population

	At Week 4			At Week 8 (exploratory outcomes)		
	Acupuncture group (*n* = 30)	Sham acupuncture group (*n* = 30)	Difference or odds ratio (95% CI)	Acupuncture group (*n* = 30)	Sham acupuncture group (*n* = 30)	Difference or odds ratio (95% CI)
Primary outcome						
Change from baseline in mean weekly pain intensity	−3.0 (0.3)	−1.6 (0.4)	−1.49 (−2.32 to −0.65)*	−3.8 (0.3)	−2.7 (0.4)	−1.33 (−2.11 to −0.54)*
Secondary outcomes						
≥30% reduction in mean weekly pain intensity	86.7 (6.3)	43.3 (9.2)	9.48 (2.59 to 42.76)*	96.7 (3.3)	76.7 (7.9)	9.75 (1.37 to 47.84)*
≥50% reduction in mean weekly pain intensity	53.3 (9.3)	20.0 (7.4)	5.34 (1.58 to 21.14)*	86.7 (6.3)	60.0 (9.1)	4.50 (1.23 to 19.66)^*^
Jaw opening and movement						
Change from baseline in pain-free jaw opening, mm	5.6 (0.7)	2.3 (0.4)	3.25 (1.76 to 4.74)*	5.1 (0.4)	2.1 (0.3)	2.94 (1.89 to 3.99)*
Change from baseline in maximum unassisted jaw opening, mm	3.4 (0.4)	1.6 (0.3)	1.99 (1.02 to 2.96)*	3.0 (0.3)	1.5 (0.3)	1.58 (0.76 to 2.41)*
Change from baseline in maximum assisted jaw opening, mm	3.0 (0.4)	1.2 (0.2)	1.83 (0.86 to 2.80)*	3.3 (0.4)	1.2 (0.2)	2.22 (1.32 to 3.11)*
Change from baseline in protrusion movement, mm	1.3 (0.2)	0.3 (0.1)	1.01 (0.59 to 1.43)*	1.6 (0.2)	0.2 (0.1)	1.37 (0.88 to 1.85)*
Change from baseline in left lateral movement, mm	1.2 (0.2)	0.4 (0.1)	0.83 (0.40 to 1.27)*	1.1 (0.2)	0.1 (0.1)	1.02 (0.62 to 1.42)*
Change from baseline in right lateral movement, mm	1.4 (0.2)	0.2 (0.1)	1.05 (0.59 to 1.50)*	1.1 (0.2)	0.2 (0.1)	0.84 (0.43 to 1.25)*
Graded chronic pain scale (GCPS)						
Change from baseline in characteristic pain intensity, 0–100 scale	−27.4 (4.0)	−15.4 (1.8)	−12.21 (−21.28 to −3.14)*	−24.1 (3.0)	−10.6 (2.5)	−13.66 (−21.95 to −5.38)*
Change from baseline in disability score, 0–100 scale	−22.5 (4.2)	−8.6 (3.3)	−14.88 (−25.85 to −3.90)*	−20.3 (4.6)	−7.9 (1.6)	−12.56 (−22.76 to −2.36)*
Jaw functional limitations scale-20 (JFLS-20)						
Change from baseline in mastication, 0–10 scale	−2.4 (0.3)	−0.9 (0.2)	−1.38 (−2.08 to −0.69)*	−2.5 (0.3)	−1.1 (0.2)	−1.36 (−1.98 to −0.73)*
Change from baseline in vertical jaw mobility, 0–10 scale	−2.3 (0.4)	−0.9 (0.1)	−1.45 (−2.34 to −0.56)*	−2.3 (0.3)	−1.0 (0.2)	−1.37 (−2.14 to −0.60)*
Change from baseline in verbal and emotional expression, 0–10 scale	−1.7 (0.3)	−0.7 (0.1)	−1.05 (−1.60 to −0.51)*	−1.8 (0.2)	−0.9 (0.2)	−0.94 (−1.52 to −0.36)*
Change from baseline in overall, 0–10 scale	−2.1 (0.3)	−0.8 (0.1)	−1.30 (−1.86 to −0.73)*	−2.2 (0.2)	−1.0 (0.1)	−1.22 (−1.68 to −0.76)*
Depression, anxiety and stress scales-21 (DASS-21)						
Change from baseline in depression	−2.2 (0.3)	−1.3 (0.3)	−0.85 (−1.65 to −0.06)*	−1.8 (0.3)	−0.9 (0.3)	−1.01 (−1.80 to −0.21)*
Change from baseline in anxiety	−1.8 (0.3)	−0.6 (0.3)	−1.10 (−1.89 to −0.30)*	−1.5 (0.4)	−0.3 (0.2)	−1.15 (−2.00 to −0.30)*
Change from baseline in stress	−2.0 (0.3)	−0.6 (0.4)	−1.38 (−2.47 to −0.29)*	−1.9 (0.3)	−0.4 (0.3)	−1.42 (−2.31 to −0.53)*
Change from baseline in overall	−5.9 (0.6)	−2.5 (0.6)	−3.33 (−5.23 to −1.43)*	−5.3 (0.8)	−1.6 (0.5)	−3.57 (−5.41 to −1.74)*
Pittsburgh sleep quality index (PSQI)						
Change from baseline in subjective sleep quality	−0.2 (0.1)	0.1 (0.1)	−0.34 (−0.65 to −0.02)*	−0.2 (0.1)	−0.0 (0.1)	−0.15 (−0.52 to 0.23)
Change from baseline in sleep latency	−0.4 (0.2)	0.2 (0.2)	−0.68 (−1.12 to −0.23)*	−0.5 (0.2)	0.0 (0.2)	−0.64 (−1.09 to −0.18)*
Change from baseline in sleep duration	−0.2 (0.2)	0.0 (0.1)	−0.20 (−0.57 to 0.17)	−0.1 (0.2)	0.0 (0.1)	−0.14 (−0.53 to 0.25)
Change from baseline in habitual sleep efficiency	−0.1 (0.2)	−0.1 (0.2)	−0.12 (−0.56 to 0.32)	−0.2 (0.2)	−0.0 (0.2)	−0.22 (−0.69 to 0.25)
Change from baseline in sleep disturbances	−0.2 (0.1)	0.0 (0.1)	−0.20 (−0.56 to 0.17)	−0.3 (0.2)	−0.2 (0.1)	−0.13 (−0.56 to 0.31)
Change from baseline in use of sleep medication	−0.2 (0.1)	−0.2 (0.2)	0.00 (−0.35 to 0.36)	−0.2 (0.1)	−0.0 (0.1)	−0.13 (−0.41 to 0.15)
Change from baseline in daytime dysfunction	−0.4 (0.1)	0.1 (0.1)	−0.44 (−0.80 to −0.08)*	−0.6 (0.1)	0.0 (0.2)	−0.49 (−0.88 to −0.11)*
Change from baseline in overall	−1.7 (0.5)	0.2 (0.5)	−1.95 (−3.34 to −0.56)*	−2.0 (0.5)	−0.1 (0.5)	−1.93 (−3.30 to −0.56)*
Quantitative sensory testing						
Pressure pain thresholds (PPTs)						
Change from baseline in masseter PPT, 0–500 kPa	3.5 (6.3)	1.3 (4.9)	3.62 (−12.70 to 19.94)	–	–	–
Change from baseline in anterior temporalis PPT, 0–500 kPa	5.7 (7.8)	1.6 (4.2)	4.20 (−14.36 to 22.76)	–	–	–
Change from baseline in sternocleidomastoid PPT, 0–500 kPa	3.8 (5.9)	2.5 (6.7)	1.44 (−17.28 to 20.15)	–	–	–
Change from baseline in trapezius PPT, 0–500 kPa	26.9 (10.7)	11.6 (5.8)	16.28 (−7.58 to 40.13)	–	–	–
Change from baseline in TMJ PPT, 0–500 kPa	25.2 (7.1)	15.5 (5.9)	9.15 (−9.78 to 28.08)	–	–	–
sEMG						
Mandibular resting position (MR)						
RMS, change from baseline in masseter muscle, left, μV	−1.3 (3.5)	−2.6 (5.1)	0.11 (−11.99 to 12.22)	–	–	–
RMS, Change from baseline in masseter muscle, right, μV	−4.1 (3.7)	−6.2 (4.4)	0.77 (−10.86 to 12.40)	–	–	–
RMS, Change from baseline in anterior temporalis muscle, left, μV	−3.8 (2.8)	−7.7 (4.6)	2.15 (−8.73 to 13.02)	–	–	–
RMS, Change from baseline in anterior temporalis muscle, right, μV	−0.4 (1.0)	−0.6 (2.7)	−0.46 (−6.35 to 5.42)	–	–	–
Habitual chewing (HC)						
RMS, change from baseline in masseter muscle, left, μV	−12.4 (12.4)	−11.0 (8.0)	−3.13 (−33.71 to 27.46)	–	–	–
RMS, change from baseline in masseter muscle, right, μV	−10.7 (22.0)	−17.6 (8.9)	7.99 (−39.71 to 55.70)	–	–	–
RMS, change from baseline in anterior temporalis muscle, left, μV	−7.5 (10.9)	−8.2 (10.6)	2.16 (−29.48 to 33.80)	–	–	–
RMS, change from baseline in anterior temporalis muscle, right, μV	−5.2 (9.6)	−17.9 (8.9)	14.30 (−12.94, 41.54)	–	–	–
Maximal voluntary contraction (MVC)						
RMS, change from baseline in masseter muscle, left, μV	−13.2 (7.8)	11.9 (11.1)	−24.69 (−51.47, 2.10)	–	–	–
RMS, change from baseline in masseter muscle, right, μV	−16.4 (14.9)	4.2 (7.2)	−20.67 (−54.72, 13.38)	–	–	–
RMS, change from baseline in anterior temporalis muscle, left, μV	−9.6 (6.3)	−8.3 (6.7)	−3.03 (−21.67, 15.60)	–	–	–
RMS, change from baseline in anterior temporalis muscle, right, μV	−0.8 (9.1)	−15.4 (5.3)	14.24 (−7.75 to 36.24)	–	–	–

Data are least squares mean (SE), mean difference (95% CI), mean percentage (SE) or odds ratio (95% CI). GCPS, graded chronic pain scale; JFLS, jaw functional limitation scale; DASS, depression, anxiety and stress scales; PSQI, Pittsburgh sleep quality index; PPT, pressure pain threshold; TMJ, temporomandibular joint; sEMG, surface electromyography; RMS, root mean square; MR, mandibular resting position; HC, habitual chewing; MVC, maximal voluntary contraction.

* *P* < 0.05.

At week 4, 86.7% and 53.3% of acupuncture group patients achieved at least 30% and 50% reduction in the mean weekly pain intensity, respectively, compared to 43.3% and 20.0% in the sham acupuncture group (*P* = 0.001 and *P* = 0.010, respectively). At week 8, 30% and 50% pain reduction occurred in 96.7% and 86.7% of the participants in the acupuncture group, and in 76.7% and 60.0% of those in the sham acupuncture group, respectively (*P* = 0.021 and *P* = 0.030, respectively; Table 2, Figures S6 and S7 of Supplement 2).

#### Physical functioning

At week 4, the acupuncture group showed significant improvements in pain-free jaw opening, maximum unassisted and assisted jaw opening, protrusion and lateral movements compared to the sham acupuncture group (all *P* < 0.001). Similar results were observed for the exploratory outcomes at week 8 (Table 2).

The acupuncture group had greater reductions in GCPS (characteristic pain intensity, week 4: *P* = 0.009; week 8: *P* = 0.002; disability score, week 4: *P* = 0.009; week 8: *P* = 0.017, Table 2), JFLS-20 (mastication, weeks 4 and 8: *P* < 0.001; vertical jaw mobility, week 4: *P* = 0.002; week 8: *P* < 0.001; verbal and emotional expression, week 4: *P* < 0.001; week 8: *P* = 0.002; overall, weeks 4 and 8: *P* < 0.001, Table 2) than the sham acupuncture group at weeks 4 and 8.

#### Emotional functioning

At weeks 4 and 8, participants in the acupuncture group showed significantly greater decreases in DASS-21 for depression (*P* = 0.036 and *P* = 0.014, respectively), anxiety (*P* = 0.008 and *P* = 0.009, respectively), stress (*P* = 0.014 and *P* = 0.002, respectively) and overall scores (all *P* < 0.001, Table 2) than those in the sham acupuncture group. They also experienced significant reductions in insomnia severity (weeks 4 and 8: *P* = 0.007). Similarly, significant patterns of change existed in sleep latency (week 4: *P* = 0.004; week 8: *P* = 0.007) and daytime dysfunction (week 4: *P* = 0.017; week 8: *P* = 0.014, Table 2).

#### Additional treatment outcomes

The PPT change from baseline over the muscles in the masseter, temporalis, sternocleidomastoid, trapezius and TMJ did not differ between the groups (masseter: *P* = 0.658; temporalis: *P* = 0.652; sternocleidomastoid: *P* = 0.878; trapezius: *P* = 0.177; TMJ: *P* = 0.337, Table 2). sEMG data on masseter and anterior temporalis muscle showed no significant differences during mandibular resting, habitual chewing and maximal voluntary contraction between the two groups (Table 2).

Participant satisfaction was higher with acupuncture than sham acupuncture at week 4 (Table S7 of Supplement 2). At baseline, both the groups showed no difference in the participants’ expectations of acupuncture (Table S8 of Supplement 2). Acupuncture compliance was similar between the groups (Table S9 of Supplement 2). The effectiveness of blinding showed no differences between the two groups, and the Bang Blinding Index indicated good blinding (Table S10 of Supplement 2).

### Safety

In our study of 60 patients, two in the acupuncture group reported AEs (subcutaneous hematoma and needling pain after treatment). No AEs occurred in the sham acupuncture group and no significant difference between the groups (*P* = 0.492; Table 3). Nonetheless, all events were mild and self-limiting, and no serious AEs were reported in either group. No clinically meaningful differences in laboratory tests, vital signs or weight were observed between the two groups (Tables S11–S12 of Supplement 2).

**Table 3.** Adverse events related to treatment^a^

	Acupuncture group (*n* = 30)	Sham acupuncture group (*n* = 30)
Overall*	2 (6.7)	0 (0)
Serious adverse event	0 (0)	0 (0)
Adverse event	2 (6.7)	0 (0)
Subcutaneous hematoma	1 (3.3)	0 (0)
Needling pain after treatment	1 (3.3)	0 (0)

Data are *n* (%).

a Adverse events were analyzed in all participants who received treatment. Adverse events were counted by type rather than frequency in the same participant. Adverse events with different types occurring in a single participant were defined as independent adverse events. An adverse event with multiple occurrences in a single participant was defined as 1 adverse event. ^*^The Fisher’s exact test was used to analyze adverse events between the two groups (*P* = 0.492).

## Discussion

### Study participants

This single-center, single-blind, randomized controlled trial demonstrated that acupuncture was more effective than sham acupuncture in reducing the change from baseline in mean weekly pain intensity at week 4, with lasting effects up to 4 weeks after treatment. Acupuncture also led to more participants achieving at least 30% and 50% pain reduction. Moreover, it yielded clinically significant improvements in physical and emotional functioning compared to sham acupuncture. Overall, the acupuncture group experienced greater and potentially longer-lasting therapeutic effects. Acupuncture’s safety over the 4-week treatment was comparable to that of sham acupuncture, aligning with previous studies and underscoring its favorable safety profile.^22^

We used a noninvasive sham Park device as the control to improve blinding and participant adherence. To our knowledge, an ideal acupuncture placebo is noninvasive. Since 1998, researchers have developed several placebo acupuncture devices, with the Streitberger, Park and Takakura devices being the most prevalent for placebo acupuncture.^29^ Subsequently, noninvasive devices have been introduced to minimize the physiological effects of skin penetration.^30^ The blinding assessment results and low dropout rate in our trial suggest a successful blinding.

In this study, blinding was achieved only for the participants, as blinding of the acupuncturist was not feasible. To minimize the bias of a single-blind study, we employed a blinded endpoint approach by using a third-party assessor (outcome assessors) who was unaware of the treatment group assignments to evaluate the endpoints.

### Comparison with other studies

A meta-analysis^20^ revealed that acupuncture effectively reduces pain in patients with TMD, especially in those with myofascial pain symptoms. Several studies^18^^,^^22^^,^^31^ have compared acupuncture with non-penetrating sham acupuncture in patients with TMD. However, varied diagnostic criteria for TMD across these trials have led to heterogeneity, hindering accurate cross-comparisons. De Salles-Neto et al.^22^ observed that acupuncture significantly reduced pain intensity than sham acupuncture (effect size: −1.72 [95% CI: −3.30 to −0.14]), similar to the effect size observed in our study. Smith et al. used immediate VAS instead of mean weekly VAS score as the primary outcome, making direct comparison with our results difficult.^18^ Our findings also differ from Zotelli et al.’s trial, which found no significant difference between acupuncture and sham acupuncture in pain reduction.^31^ The inconsistency may largely stem from different treatment strategies. Unlike most trials not showing long-term effects of acupuncture for TMD, our study indicated sustained benefits during follow-up. The effect size for acupuncture (−1.49) was comparable to that of other conservative treatments such as propranolol (−1.8)^32^ and botulinum toxin A (−1.16).^33^

### Strengths and limitations of this study

This trial’s advantages include using a validated DC/TMD examination^5^ for participant selection and diagnostic monitoring, primary outcomes sourced from TMD diaries to eliminate recall bias, excellent participant retention and diary compliance, and exploration of acupuncture’s long-term effects on patients with TMD.

This study had several limitations. The external validity of this single-center trial is limited. While multicenter studies offer more participant diversity, single-center trials allow for easier quality control and better internal authenticity. Second, although the study’s sample size is small, it was estimated from a previous study and yields nearly 100% statistical power according to our results. Third, blinding acupuncturists was unfeasible due to the nature of acupuncture. However, maintaining consistent rituals across groups and minimizing communication between acupuncturists and patients helped reduce bias from unblinded acupuncturists to some extent. Finally, this trial was conducted in a culture with a strong belief in acupuncture’s benefits, which might differ from other cultures. Treatment expectations, influenced by cultural factors, can affect efficacy, so these findings should be cautiously extrapolated to other populations.

### Clinical relevance

According to the American Association of Dental Research, conservative treatments should be the primary intervention for TMDs.^1^ Conservative treatments such as medications, occlusal splints, self-management instructions, behavioral modification and physiotherapy are endorsed for the initial care of nearly all TMDs. Medications, such as analgesics, corticosteroids and muscle relaxants, are generally used to relieve pain^34^; however, drug misuse and abuse, adverse effects and limited evidence of efficacy are of concern in the pharmacologic management of TMDs; thus, the clinician should cautiously use them.^14^ Occlusal splints have been widely used to treat patients with TMD. A recent systematic review concluded that their efficacy is greater than that of placebo; however, occlusal splints may not change pain intensity.^35^ The complications from the excessive or incorrect use of any appliance include caries, gingival inflammation, speech difficulties and psychological dependence on the appliance.^36^^,^^37^ Our results reveal that acupuncture can be an effective alternative treatment for patients with TMD for substantial pain relief and jaw function improvement. Understanding the potential efficacy, benefits and risks of acupuncture may provide clinical evidence for patients with contraindications to other conservative treatment.

According to the World Health Organization’s global report on traditional and complementary medicine, by 2018, 98 member states had developed national policies on traditional medicine, and 109 had enacted national laws or regulations on traditional medicine.^38^ However, in countries where traditional medicine is not institutionalized, only conventional medical doctors are allowed to practice acupuncture.^39^ Combining traditional medicine interventions with conventional treatment is more likely to be effective and synergistic.^40^ Therefore, comparing conventional medicine combined with traditional medicine and conventional medicine alone could be a valuable target for future research.

In conclusion, acupuncture proved safer and more effective than sham acupuncture for patients with TMD, providing greater pain relief and improving physical and emotional function. These beneficial effects persisted after treatment discontinuation. Acupuncture may represent an important alternative treatment option for TMD-associated pain, particularly for patients contraindicated for other conservative treatments or who are intolerant to conventional therapeutic drugs.

## Supplementary Material

hcae094_Supplementary_Data

## Data sharing

Anonymized individual clinical data will be made available upon request from the corresponding author (libin@bjzhongyi.com) for research (non-commercial) purposes to individuals affiliated with academic or public health institutions.
