# Supplementary material for: Effect of acupuncture for temporomandibular disorders: a randomized clinical trial
Source: QJM. 2024 May 6;117(9):647–56. doi: 10.1093/qjmed/hcae094 (PMC11537310; doi:10.1093/qjmed/hcae094)
Supplement: hcae094_Supplementary_Data [file hcae094_supplementary_data.zip › hcae094_Supplementary_Data/Supplement_1-20240503.docx]

**SUPPLEMENT** **1:** **Study** **Protocol** **and** **Statistical** **Analysis** **Plan**

**Effect of acupuncture for temporomandibular disorders: a randomized clinical trial**

**Contents**

**Study** **Protocol 2**

**Statistical** **Analysis** **Plan**  **76**


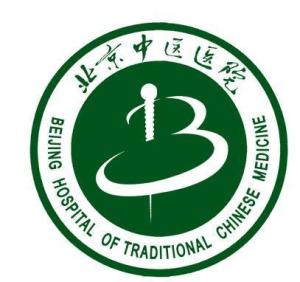


**Effect of acupuncture for temporomandibular disorders: a randomized clinical trial**

**STUDY PROTOCOL**

**Beijing Hospital of Traditional Chinese Medicine, Capital Medical University, Beijing, China**

**Principal Investigator**

**Bin Li, MD, PhD**

**Version: 1.1**

**5 March 2019**

**TABLE OF CONTENTS**

**[LIST OF ABBREVIATIONS 4](#_Toc1951)**

**[1. INTRODUCTION 6](#_Toc6872)**

[1.1 Background 6](#_Toc8325)

[1.1.1 Overview 6](#_Toc7974)

[1.1.2 Clinical Data 7](#_Toc19257)

[1.2 Rationale for the Study 7](#_Toc2639)

**[2. OBJECTIVES AND OUTCOMES 8](#_Toc4490)**

**[3. STUDY DESIGN 10](#_Toc17170)**

[3.1 Overview of the Study Design 10](#_Toc23235)

[3.2 Rationale for the Study Design 13](#_Toc18694)

[3.3 Justification of Study Design 13](#_Toc28587)

[3.4 Randomization and Blinding 13](#_Toc13086)

[3.4.1 Randomization 14](#_Toc24461)

[3.4.2 Blinding/Unblinding 14](#_Toc24342)

[3.5 Withdrawal from Study 14](#_Toc22121)

[3.6 Lost to Follow-up 15](#_Toc16731)

**[4. STUDY POPULATION](#_Toc13752)** [15](#_Toc13752)

[4.1 Participant Recruitment 15](#_Toc307)

[4.2 Inclusion Criteria 15](#_Toc3193)

[4.3 Exclusion Criteria 15](#_Toc11058)

**[5. INTERVENTION](#_Toc12372)** [16](#_Toc12372)

[5.1 Acupuncture group 17](#_Toc21564)

[5.2 Sham acupuncture group 18](#_Toc2320)

**[6. CONCOMITANT MEDICATIONS 19](#_Toc6558)**

**[7. STUDY VISIT PLAN 20](#_Toc26821)**

[7.1 Screening Period 20](#_Toc3855)

[7.2 Baseline Period 20](#_Toc24158)

[7.3 Treatment Period 21](#_Toc29080)

[7.4 Follow-up Period 21](#_Toc7531)

**[8. ASSESSMENT](#_Toc28322)** [22](#_Toc28322)

[8.1 Screening and Baseline Procedures and Assessments 22](#_Toc11608)

[8.1.1 Demographics and Baseline Characteristics 22](#_Toc2258)

[8.2 Efficacy Assessment 22](#_Toc25474)

[8.2.1 Use of Clinical Outcome Assessment 22](#_Toc12368)

[8.2.2 TMD Diary 23](#_Toc1723)

[8.2.3 Facial Disability Index (FDI) 23](#_Toc4775)

[8.2.4 Graded Chronic Pain Scale (GCPS) 24](#_Toc12358)

[8.2.5 Jaw Functional Limitations Scale-20 (JFLS-20) 26](#_Toc1648)

[8.2.6 Depression Anxiety Stress Scales-21 (DASS-21) 27](#_Toc23886)

[8.2.7 Pittsburgh Sleep Quality Index (PSQI) 27](#_Toc22866)

[8.2.8 Pressure Pain Threshold Measurements 29](#_Toc32091)

[8.2.9 Surface Electromyography (sEMG) 30](#_Toc15148)

[8.2.10 Acupuncture Expectancy Scale 31](#_Toc28007)

[8.2.11 Patient’s satisfaction 31](#_Toc5983)

[8.2.12 Blinding assessment 31](#_Toc3181)

[8.3 Order of Assessments 32](#_Toc30097)

[8.4 Treatment Compliance 33](#_Toc15792)

**[9. ADVERSE EVENTS](#_Toc1376)** [33](#_Toc1376)

[9.1 Definitions of Adverse Event 33](#_Toc3604)

[9.1.1 Adverse Event Definitions 34](#_Toc21730)

[9.1.2 Serious Adverse Event Definitions 35](#_Toc7467)

[9.1.3 AE Assessment Definitions 35](#_Toc28641)

[9.2 Recording of Adverse Event 36](#_Toc12282)

[9.3 Reporting of Serious Adverse Event 37](#_Toc22066)

[9.4 Treatment and Follow-up Visits of Adverse Events 38](#_Toc17012)

[9.5 Pregnancy 38](#_Toc27038)

[9.6 Clinical Safety Laboratory Tests 39](#_Toc22079)

[9.7 Vital Signs 40](#_Toc14385)

[9.8 Height and Weight 40](#_Toc28743)

[9.9 Management of Reactions to Treatment 40](#_Toc28147)

**[10. ETHICS](#_Toc1052)** [40](#_Toc1052)

[10.1 Ethical Rationale 40](#_Toc20807)

[10.2 Informed Consent 41](#_Toc17217)

[10.3 Personal Data Protection 42](#_Toc1939)

[10.4 Research Ethics Committees 43](#_Toc14279)

**[11. DATA HANDING AND RECORD KEEPING](#_Toc3039)** [43](#_Toc3039)

[11.1 Data Collection 43](#_Toc3307)

[11.1.1Electronic Case Report Forms (eCRFs) 43](#_Toc20349)

[11.1.2 Patient Binders 43](#_Toc15767)

[11.2 Database Management and Quality Control 44](#_Toc32006)

[11.3 Retention of Study Documents 44](#_Toc24564)

**[12. STATISTICAL METHOLOGY](#_Toc22572)** [45](#_Toc22572)

[12.1 Analysis Sets 45](#_Toc15977)

[12.1.1 Intention-to-Treat Analysis Set 45](#_Toc18740)

[12.1.2 Per-Protocol (PP) Analysis Set 45](#_Toc12734)

[12.1.3 Safety Analysis Set 45](#_Toc22798)

[12.2 Sample Size and Power 45](#_Toc3394)

[12.3 Descriptive Statistics 45](#_Toc19305)

[12.4 Participants Disposition 46](#_Toc13597)

[12.5 Demographics and Baseline Characteristics 46](#_Toc11832)

[12.6 Efficacy analyzes 46](#_Toc30774)

[12.6.1 General Efficacy Analysis Methodology 46](#_Toc12924)

[12.6.2 Primary Analysis of the Primary Outcome 46](#_Toc21083)

[12.6.3 Analysis of the Secondary Outcomes 46](#_Toc88)

[12.6.4 Analysis of the Exploratory Outcomes 47](#_Toc8328)

[12.7 Safety analyzes 47](#_Toc2224)

[12.7.1 Acupuncture Administration 47](#_Toc24703)

[12.7.2 Adverse Events 47](#_Toc10475)

[12.7.3 Clinical Laboratory Tests/ Vital Signs/ Weight 48](#_Toc7698)

**[13. MONITORING PROCEDURES](#_Toc22921)** [48](#_Toc22921)

[13.1 Procedures for Monitoring Participant Compliance 48](#_Toc974)

[13.2 Study Monitoring 48](#_Toc9904)

**[14. STUDY DISCONTINUATION 49](#_Toc29159)**

**[15. STUDY ORGANISATION 49](#_Toc27102)**

[15.1 Steering Committee 49](#_Toc21907)

[15.2 Executive Committee 49](#_Toc13916)

[15.3 Data Monitoring Committee 50](#_Toc3128)

**[16. PUBLICATIONS 50](#_Toc13870)**

**[17. FUNDING 50](#_Toc14620)**

**18. SUMMARY OF CHANGES TO PROTOCOL................................................. [50](#_Toc14620)**

**[APPENDIX 50](#_Toc26996)**

[APPENDIX A. TMD Diary 52](#_Toc11064)

[APPENDIX B. Facial Disability Index scale (FDI) 55](#_Toc517)

[APPENDIX C. Graded Chronic Pain Scale Version 2.0 60](#_Toc25770)

[APPENDIX D. Jaw Functional Limitation Scale-20 62](#_Toc27663)

[APPENDIX E. Depression Anxiety Stress Scale-21 (DASS-21) 63](#_Toc15487)

[APPENDIX F. Pittsburgh Sleep Quality Index (PSQI) 65](#_Toc581)

[APPENDIX G. Pressure pain threshold (PPT) 68](#_Toc2784)

[APPENDIX H. Surface Electromyography (sEMG) 69](#_Toc9655)

[APPENDIX I. Acupuncture Expectancy Scale 70](#_Toc12269)

[APPENDIX J. Participant’s compliance 71](#_Toc22278)

[APPENDIX K. Participants’ satisfaction scale 72](#_Toc20158)

[APPENDIX L. Blinding assessment 73](#_Toc9726)

**LIST OF ABBREVIATIONS**

| **Abbreviation** | **Definition** |
| --- | --- |
| AE | Adverse Event |
| AES | Acupuncture Expectancy Scale |
| BMI | Body Mass Index |
| CIs | Confidence Intervals |
| CRF | Case Report Form |
| CPI | Characteristic Pain Intensity |
| DASS-21 | Depression Anxiety and Stress Scale-21 |
| DC/TMD | Diagnostic Criteria for Temporomandibular disorders |
| eCRF | Electronic Case Report Form |
| FDI | Facial Disability Index |
| GCP | Good Clinical Practice |
| GCPS | Graded Chronic Pain Scale |
| HC | Habitual Chewing |
| ITT | Intention-to-Treat |
| JFLS-20 | Jaw Function Limitation Scale-20 |
| LSM | Least-Squares Mean |
| MVC | Maximum Voluntary Contraction |
| NSAIDs | Nonsteroidal Anti-inflammatory Drugs |
| PP | Per-Protocol |
| PPT | Pressure Pain Threshold |
| PSQI | Pittsburgh Sleep Quality Index |
| RCT | Randomized Controlled Trial |
| RMS | Root Mean Square |
| SAP | Statistical Analysis Plan |
| SAE | Serious Adverse Event |
| SE | Standard Error |
| sEMG | Surface Electromyogram |
| SD | Standard Deviation |
| SOP | Standard Operating Procedures |
| SUSARs | Suspected, Unexpected, Serious, Adverse Reactions |
| TENS | Transcutaneous Electrical Nerve Stimulation |
| TMD | Temporomandibular Disorders |
| VAS | Visual Analogue Scale |
| WHO | World Health Organization |

**1. INTRODUCTION**

**1.1 Background**

**1.1.1 Overview**

Temporomandibular Disorders (TMD) refer to a group of symptoms that may originate from the temporomandibular joint, muscles of mastication, and their associated structures,^1^ affect 5-12% of the population mainly in younger persons.^2^ It is considered the first leading cause of pain and disability among frequently occurring facial pain and the second among musculoskeletal conditions with an annual cost estimated at $4 billion.^3^ The diagnostic criteria of TMD have evolved over the last three decades to achieve the most reliable and valid diagnosis. The recent Diagnostic Criteria classify TMD (DC/TMD) into two groups, one of which is pain-related disorders (including myalgia, arthralgia, and headache attributed to TMD).^2^ Jaw pain is the most common symptom that compels patients to seek treatment, with an average TMD pain intensity rating of 5.0 on a 11-point scale in patients with TMD.^4^ In addition to facial pain, TMD patients frequently report a high level of comorbid idiopathic pain conditions such as headaches, depression, sleep disorders, and fibromyalgia.^5-7^

The main objectives of the management of patients with TMD are reducing pain, increasing temporomandibular joint function, and relieving the reflex masticatory muscle spasm.^8^ Common treatments include conservative treatment (pharmacotherapy and non-pharmacotherapy), minimally invasive surgical procedures, and invasive surgical procedures.^9 10^ Pharmacotherapy is considered to be efficacy for pain reduction in the treatment of TMDs, which mostly includes muscle relaxants, nonsteroidal anti-inflammatorydrugs (NSAIDs), analgesics, tricyclic antidepressants, benzodiazepines, and corticosteroids.^8 9^ However, one review reported that pharmacotherapy for TMD is not commonly used and can be considered as a complementary therapy rather than a treatment itself.^8^ A Cochrane review evaluating TMD medications also found lacking evidence to support or refute the efficacy of any drug for the treatment of TMD pain.^11^ And several reviews are considered its effect on pain relieving but less effect on improving joint function.^11 12^ Moreover, not all patients benefit from this approach and in many patients pain and masticatory dysfunction persist despite a range of treatments.^13^ Non-pharmacotherapy also encompasses cognitive behavioral therapy, physiotherapy, occlusal splint therapy, acupuncture, and even education.^8^ Evidence-based conservative non-pharmacotherapy should be the first-line treatment in TMD patients due to the low risk of side effects and their reversibility.^1 8 14^

Acupuncture, recommended by WHO as a non-pharmacotherapy in multiple pain management,^15^ has long been recognized as a potentially effective non-pharmaceutical therapy for TMD treatment.^16^ Systematic reviews suggested that acupuncture was found to be effective in alleviating pain and masseter muscle tenderness in patients with TMD, though limited by substantial bias.^17 18^ RCTs have suggested that acupuncture is an effective treatment of TMD, particularly for facial pain relieving and mandibular function.^16 19 20^ However, several randomized clinical trials found no differences between real acupuncture and sham acupuncture.^21 22^ The inconsistent findings may be ascribed to variations in placebo control settings and study design.^16 21^ Blinding is challenging in trials of non-drug treatments. An appropriate placebo acupuncture design should be both physiologically inert and indistinguishable from true acupuncture. A validation study has provided evidence of the ‘Park Sham Device’ which enables adequate blinding of the participant while maintaining an identical therapeutic setting to that of real acupuncture.^23^

Since well-designed pragmatic randomized controlled trials with standardized and patient-based outcome measures in TMD are urgently needed.^20^ We designed this randomized, sham-controlled, single-blind clinical trial to determine whether a 4-week course of real acupuncture, compared with sham acupuncture (Park sham device) alleviates the pain intensity or joint functional disability in patients with TMD.

**1.1.2 Clinical Data**

RCTs in China and overseas have paid close attention to the effect of acupuncture in the management of TMD.^16^ And previous studies on the effect of acupuncture on TMD showed that acupuncture was effective in relieving decreasing pain intensity of TMD.^21^ However, the trial had several limitations, the most relevant is the use of an active comparator without sham devices, which could not rule out the possibility of a placebo effect. Therefore, we designed a randomized, single-blind, randomized controlled trial to further confirm the efficacy of acupuncture as TMD.

**1.2 Rationale for the Study**

There is a proportion of participants who do not respond to or cannot tolerate existing treatments and there is a need for treatments which are more effective and better tolerated than the pharmacologic treatment. For this reason, acupuncture offers a new direction for the treatment of TMD. Acupuncture has potential advantages in the following populations: people with contraindications to medications and intolerance to conventional therapeutic drugs; people who do not want to be treated with medications; and special populations, such as pregnant women and adolescents. In addition, acupuncture is less likely to lead to medication overuse and can provide participants with individualized treatment plans. Thus, the current study will evaluate the efficacy and safety of acupuncture in this target population and provide evidence-based treatment guidance for these participants with TMD. The efficacy will be studied over a 4-week treatment period and 4-week follow-up period, which will allow long-term investigation of the efficacy and safety profile of acupuncture.

**2. OBJECTIVES AND OUTCOMES**

| Objectives | Outcomes |
| --- | --- |
| Primary Objectives  •To evaluate the efficacy of acupuncture for the temporomandibular disorder participants | •Primary Outcomes  - Change from baseline in mean weekly pain intensity at week 4  •Secondary Outcomes  - ≥30% reduction in mean weekly pain intensity at week 4  - ≥50% reduction in mean weekly pain intensity at week 4 |
| Secondary Objectives  •To evaluate the physical functioning  •To evaluate the emotional functioning  •To evaluate the additional treatment outcomes | •Secondary Outcomes  - Mean change from baseline in jaw opening and movement at week 4  - Mean change from baseline in Graded Chronic Pain Scale (GCPS) at week 4  - Mean change from baseline in Jaw Functional Limitations Scale-20 (JFLS-20) at week 4  - Mean change from baseline in Depression, Anxiety and Stress Scales-21 (DASS-21) at week 4  - Mean change from baseline in Pittsburgh sleep quality index (PSQI) at week 4  - Mean change from baseline in Pressure Pain Threshold (PPT) at week 4  - Mean change from baseline in surface electromyogram (sEMG) at week 4 |
| Exploratory Objectives  •To evaluate the long-term efficacy of acupuncture | Exploratory Outcomes  - Mean change from baseline in weekly pain intensity at week 8  - ≥30% reduction in pain intensity at week 8  - ≥50% reduction in pain intensity at week 8  - Mean change from baseline in jaw opening and movement at week 8  - Mean change from baseline in Graded Chronic Pain Scale (GCPS) at week 8  - Mean change from baseline in Jaw Functional Limitations Scale-20 (JFLS-20) at week 8  - Mean change from baseline in Depression, Anxiety and Stress Scales-21 (DASS-21) at week 8  - Mean change from baseline in Pittsburgh sleep quality index (PSQI) at week 8 |
| Safety Objectives  •To evaluate the safety of acupuncture | •Safety Outcomes  - Occurrence of adverse events throughout the study  - Abnormal vital signs (systolic and diastolic blood pressure, pulse, and respiratory rate)  - Weight  - Potentially clinically significant abnormalities in clinical laboratory tests result (blood routine test, blood biochemistry test, blood coagulation test, urinalysis test and urine pregnancy test) |

**3. STUDY DESIGN**

**3.1 Overview of the Study Design**

This study is a single-blind, randomized controlled trial in China. The study will comprise a 1-week screening period, a 1-week baseline period, a 4-week treatment period, and a 4-week follow-up period.

An overview of the study is presented in **Figure 1**.


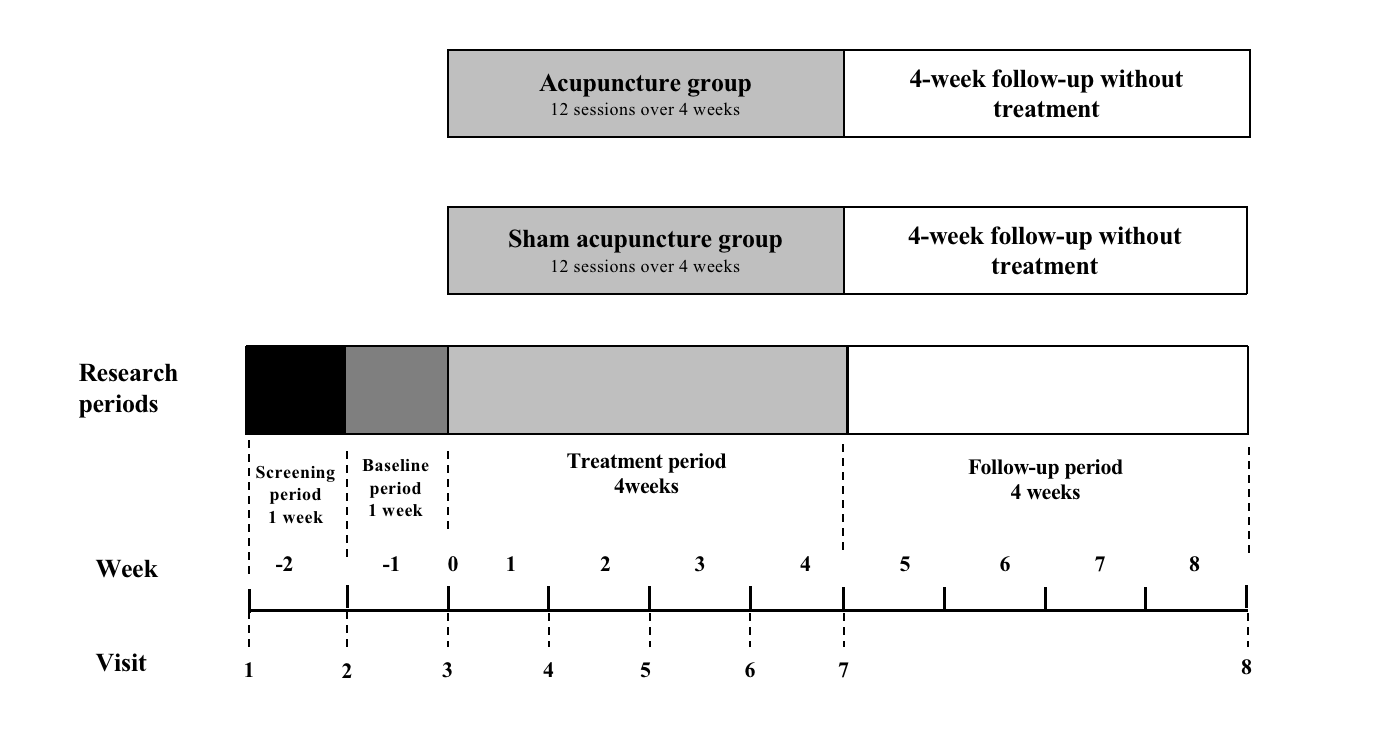


**Figure 1. Study design.** The study for each participant will be divided into 4 periods: a 1-week screening period, a 1-week baseline period, a 4-week treatment period and a 4-week follow-up period.

This study will include female and male participants, aged 18 to 80 years, suffering from temporomandibular disorder for at least three months before screening. Participants meet pain-related TMD and pain diagnosis of the Diagnostic Criteria of Temporomandibular disorders (DC/TMD). See Inclusion Criteria for further details on diagnosis.

The patients were allowed to take acute pain medications (nonsteroidal anti-inflammatory drugs, acetaminophen, or aspirin) prescribed by our dentist. We required that the same acute pain medication be used throughout the baseline period and the duration of the treatment period, which was documented throughout the course of the study. TMD-specific medication (corticosteroids, benzodiazepines, sedative hypnotics, muscle relaxants, opioids, antidepressants, and anticonvulsants) were disallowed throughout the study. Any new prescription medication, injection therapy for pain (e.g., tender or trigger point injections, steroid injections), acupuncture, biofeedback, transcutaneous electrical nerve stimulation (TENS), or oral splint should not be commenced for the management of pain throughout the study.

After completing the informed consent process at the end of screening period, participants will be screened for eligibility. Eligible participants will be randomly assigned in a 1:1 ratio to acupuncture group or sham acupuncture group.

Pain information will be captured daily during the entire study using a TMD diary. Additional assessments of physical functioning (measured by jaw opening and movement, Graded Chronic Pain Scale [GCPS] and Jaw Functional Limitations Scale-20 [JFLS-20]), the emotional functioning (measured by Depression, Anxiety and Stress Scales [DASS-21] and Pittsburgh sleep quality index [PSQI]), and additional treatment outcomes (PPT and sEMG) will be measured at the end of baseline, treatment period, and follow-up period (week 0, week 4, week 8).

The assessments are summarized in **Table 1.** An independent data monitoring committee will oversee the data during the study.

**Table 1. The schedule of enrolment, interventions, and assessments**

| **STUDY PERIOD** | | | | | | | | |
| --- | --- | --- | --- | --- | --- | --- | --- | --- |
|  | **Screening** | **Baseline** | **Allocation** | **Treatment** | | | | **Follow-up** |
| **TIMEPOINT**  **(W, week)** | -2 | -1 | 0 | 1 | 2 | 3 | 4 | 8 |
| **Enrolment** | | | | | | | | |
| Informed consent | **×** |  |  |  |  |  |  |  |
| Eligibility criteria | **×** | **×** |  |  |  |  |  |  |
| Demography Characteristics |  | **×** |  |  |  |  |  |  |
| Disease history of TMD |  | **×** |  |  |  |  |  |  |
| Randomization |  |  | **×** |  |  |  |  |  |
| **Interventions** | | | | | | | | |
| Acupuncture group |  |  |  |  | | | |  |
| Sham acupuncture group |  |  |  |  | | | |  |
| **Assessments** | | | | | | | | |
| TMD diary |  |  | | | | | | |
| VAS |  |  | **×** |  |  |  | **×** | **×** |
| Jaw opening and movement |  |  | **×** |  |  |  | **×** | **×** |
| GCPS |  |  | **×** |  |  |  | **×** | **×** |
| JFLS-20 |  |  | **×** |  |  |  | **×** | **×** |
| DASS-21 |  |  | **×** |  |  |  | **×** | **×** |
| PSQI |  |  | **×** |  |  |  | **×** | **×** |
| PPT |  |  | **×** |  |  |  | **×** |  |
| sEMG |  |  | **×** |  |  |  | **×** |  |
| Laboratory test* |  |  | **×** |  |  |  | **×** |  |
| Acupuncture expectancy scale |  |  | **×** |  |  |  |  |  |
| Participant’s satisfaction scale |  |  |  |  |  |  | **×** |  |
| Participant’s compliance |  |  |  | **×** | **×** | **×** | **×** |  |
| Blinding assessment |  |  | **×** |  |  |  | **×** |  |
| Clinical laboratory tests |  |  | **×** |  |  |  | **×** |  |
| Vital signs |  |  | **×** |  |  |  | **×** | **×** |
| Weight |  |  | **×** |  |  |  | **×** | **×** |
| Adverse events |  |  |  | | | | | |

VAS=visual analogue scale；GCPS=Graded Chronic Pain Scale；JFLS-20=Jaw Function Limitation Scale-20；DASS-21=Depression Anxiety and Stress Scale-21；PSQI=Pittsburgh Sleep Quality Index.

*The laboratory test includes blood and urine samples for the clinical safety.

The overall end of the study is defined as the last protocol-specified contact with the last participant ongoing in the study.

**3.2 Rationale for the Study Design**

The current study is a 8-week (4-week treatment period and 4-week follow-up period) single-blind, randomized controlled trial to investigate whether acupuncture can lead to clinically significant improvement in TMD patients. The primary outcome is change from baseline in mean weekly pain intensity at week 4. Additionally, outcomes will be evaluated at week 8 to investigate if the relative efficacy is sustained. The follow-up period enables further investigation on the long-term efficacy and safety and for further insights into the effectiveness profile of acupuncture. Outcomes evaluating physical functioning and emotional functioning, are included in the study to demonstrate the impact of treatment beyond the relief in TMD facial pain.

The acupuncture prescriptions will be developed based on the information in classical and modern literature^18 24 25^ and experience from the previous study.^16^ Acupoints selection will include bilateral acupoints and affected-side acupoints based on the principle of meridian circulation in the pain region according to the theory of Traditional Chinese Medicine.

The sample size of 60 patients for the primary outcome is based on our pilot study, on the expected change from baseline to week 4 in pain intensity and should provide adequate power for detection of a clinically meaningful treatment effect.

**3.3 Justification of Study Design**

In order to blind the treatment arms between acupuncture treatment, acupuncture and sham acupuncture will conduct using the acupuncture device that look similar in appearance during treatment period.

**3.4 Randomization and Blinding**

**3.4.1 Randomization**

Eligible participants will be randomly assigned (1:1) to either acupuncture group or sham acupuncture group using an interactive web-based response system (Beijing LNKMED Tech Co., Ltd, Beijing, China) The randomization sequence will be computer generated by an independent statistician who do not participate in the trial. After a participant’s eligibility is determined, a dedicated investigator will access the randomization interface and completed randomization form (each participant’s name, sex, and birthday), and then the random number and group assignment will be displayed on the web interface. Afterwards, this dedicated investigator will inform acupuncturists the randomization.

**3.4.2 Blinding/Unblinding**

The participants, outcome assessors, and the statistician will be blinded to treatment assignment. Unmasked personnel will include the dedicated investigator responsible for the randomization module. Because of the nature of the intervention, acupuncturists cannot be blinded, but they will be trained not to communicate with participants or outcome assessors about treatment procedures and responses.

In case of a serious adverse event (SAE) or pregnancy, or in cases when knowledge of the study treatment assignment is needed to make treatment decisions, the participants may unblind the treatment assignment as deemed necessary, mainly in emergency situations. When a blind is broken, the participant will be withdrawn from the study, and the event will be recorded onto the case report forms (CRFs). The circumstances leading to the breaking of the code should be fully documented in the investigator’s study files and in the participant’s source documentation. Treatment assignments should not be recorded in any study documents or source documents.

**3.5 Withdrawal from Study**

Withdrawal of consent occurs when a participant does not want to participate in the study anymore, does not want any form of follow-up, or does not want any further study related contacts. Participants have the right to withdraw from the study at any time without prejudice to their future treatment.

If a participant withdraws consent, the investigator must make every effort to determine the primary reason for this decision. If the withdrawal is due to adverse events (AEs), refer to the **section 9**. Participants who have withdrawn from the study cannot be included again in the study.

**3.6 Lost to Follow-up**

For participants whose status are unclear because they fail to appear for study visits without stating an intention to withdraw, the investigator should make every effort to contact the participant. A participant should not be considered lost to follow-up until the end of the study.

**4. STUDY POPULATION**

**4.1 Participant Recruitment**

Trial participants with temporomandibular disorder will be recruited by investigators from outpatient clinics at the Beijing Hospital of Traditional Chinese Medicine, Capital Medical University. Meanwhile, information flyers introducing the details of the trial will be posted at the outpatient clinics for greater exposure. The investigators will be notified immediately when the recruitment period comes to an end. An examiner-verified will make the diagnosis of TMD on the basis of the DC/TMD.

**4.2 Inclusion Criteria**

Patients were eligible to be included in the study only if they met all of the following criteria at screening:

• be male or female, aged between 18 and 80 years;

• Suffer from pain-related TMD in accordance with DC/TMD, including myalgia (local myalgia, myofascial pain, myofascial pain with referral), arthralgia and headache attributed to TMD.

• Suffer from pain for at least three months;

• Provide a signed and dated informed consent form.

**4.3 Exclusion Criteria**

Patients were excluded from study enrolment if they met any of the following criteria at screening:

**Diagnostics Assessments:**

• Meet the diagnosis of disc displacement with reduction and locking, disc displacement without reduction and limitation of mouth opening, disc displacement without reduction without limitation of mouth opening, degenerative joint disease, and subluxation according to DC/TMD;

**Prior/Concomitant Therapy:**

• Have commenced occlusal splint therapy for the management of facial pain within 1 month prior to the screening period;

• Used any injection therapy (e.g., tender or trigger point injections, steroid injections) for the management of pain within 1 month prior to the screening period

• Used acupuncture, biofeedback, or TENS for the management of pain within 1 month prior to the screening period;

• Used TMD-specific medication (corticosteroids, benzodiazepines, sedative hypnotics, muscle relaxants, opioids, antidepressants, and anticonvulsants) intervention within 1 month prior to the screening and baseline period.

**Medical Conditions:**

• Suffer from pain of dental origin, trauma, sinus pathology, neuropathic origin, inflammatory systemic diseases and cancer;

• Have a history of rheumatoid arthritis;

• Patients with mental illness or substance abuse;

• Be pregnant, lactating, or planning to become pregnant during the study.

**5. INTERVENTION**

We chose the acupuncture prescriptions as a result of the information in classical and modern literature^18 24 25^ and experience from the previous study.^16^ Acupuncture treatment will be performed by two licensed acupuncturists who had at least five years of acupuncture experience. All the acupuncturists will be trained how to locate acupoints, puncture and manipulate needles and acupuncture device before the trial. Participants will be treated in a single treatment room for privacy and to avoid communication. The acupuncturists will be asked to have the least possible communication with the participants to minimize bias. There are two groups in this trial: acupuncture group and sham acupuncture group. The location of acupoints and manipulations of acupuncture and sham acupuncture are shown in **Table 2 and Table 3.**

Participants will receive acupuncture treatment for 4 weeks and be given twelve 30-minute sessions (3 sessions per week, ideally every other weekday).

**Table 2. Location of acupoints**

| **Kinds of acupoints** | **Acupoints** |
| --- | --- |
| Bilateral acupoints | Hegu (LI4), Yanglingquan (GB34) |
| Acupoints in the affected side(s) | Tinggong (SI19), Jiache (ST6), Xiaguan (ST7) |

**Table 3. Similarities and differences among two groups**

|  | **Acupuncture group** | **Sham acupuncture group group** |
| --- | --- | --- |
| Kinds of points stimulated | Acupoints | Acupoints |
| Needle | Real acupuncture needle | Sham acupuncture needle |
| Acupuncture device | Normal guide device | Park sham device |
| Number of acupoints | 7 or 10 | 7 or 10 |
| Depth of penetration, mm | 10-15 | 0 |
| Deqi | Yes | No |
| Needle retention, min | 30 | 30 |
| Number of sessions | 12 | 12 |

**5.1 Acupuncture group**

In the acupuncture group, Hegu (LI4), Yanglingquan (GB34) are selected as bilateral acupoints and Tinggong (SI19), Jiache (ST6), Xiaguan (ST7) are selected as affected-side acupoints. Acupuncturists will use 75% alcohol pads to sterilize the skin around the acupoints. Disposable, single-use real stainless steel needles (DONGBANG Acupuncture Inc, Korea) will be used for acupuncture treatment in this trial. The number of needles will be 7 or 10 in each session for both groups. Needles of 0.35mm in diameter and 70mm in length will be used for acupoints. The real acupuncture needles are applied through a holder (normal guide device) **(Figure 3)** which is held in place on the skin by a self-adhesive pad. All needles will be inserted 10-15mm in depth and twirling lifting, and thrusting (needle manipulation) will be performed for at least 10 seconds and repeated a total of 4 times with an interval of 10 minutes. The guide tube was slid forward within the Park tube, then, twirling, lifting, and thrusting (needle manipulation) were performed for at least 10 s and repeated four times at an interval of 10 min to produce a characteristic sensation known as deqi (a sensation of soreness, numbness, distention, or heaviness that indicates effective needling).


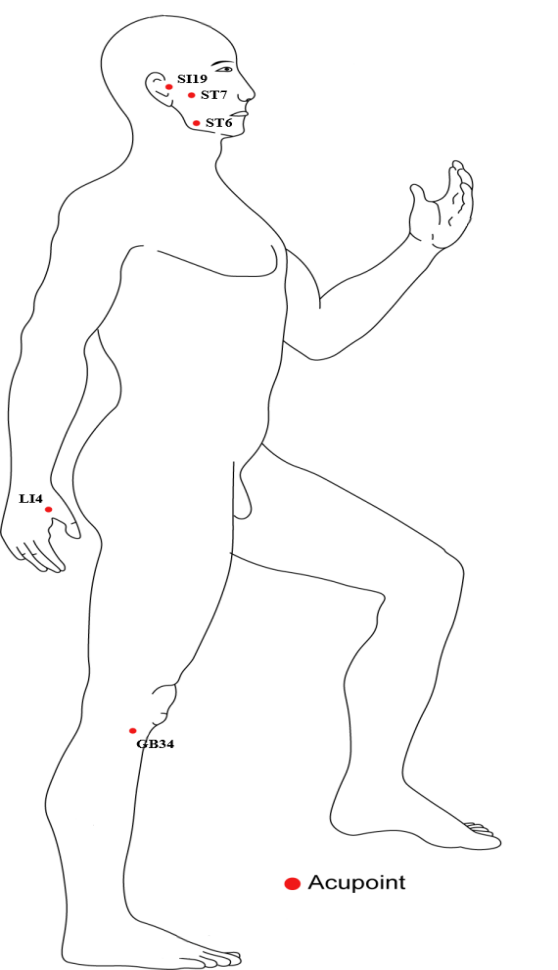


**Figure 2. Location of acupoints**

**5.2 Sham acupuncture group**

In the sham acupuncture group, the acupoints will be conducted as same as acupuncture group. The sham acupuncture needles were used in this group which looks exactly like a real needle, but is blunt and free to slide within its handle so that, when pressed, it telescopes into the handle rather than penetrating the skin. This needle is the same size as the real one (0.35 mm ×70 mm) and it is stainless steel and manufactured by DONGBANG Acupuncture Inc, Korea (**Figure 3**). In order to allow the sham needle to be retained in position at the acupoint for the same duration as a real needle, the Park sham device were used. Both the blunt sham acupuncture needles were applied through a holder (**Figure 3**) which is held in place on the skin by a self-adhesive pad. The number of needles will be 7 or 10 in each session, which is the same as the acupuncture group. The similarities and differences between acupuncture and sham acupuncture are summarized in **Table 3**. Both the blunt sham acupuncture needles were applied through a holder (**Figure 3**) which was held in place on the skin by a self-adhesive pad. The rituals of manipulations were same as real acupuncture without deqi. The acupuncture group received the true penetrative needle with a sharp point. This needle was inserted 5–10 mm into the skin. For the sham acupuncture group, the blunt needle end only touched the pad, and the handle was able to slide down the needle.


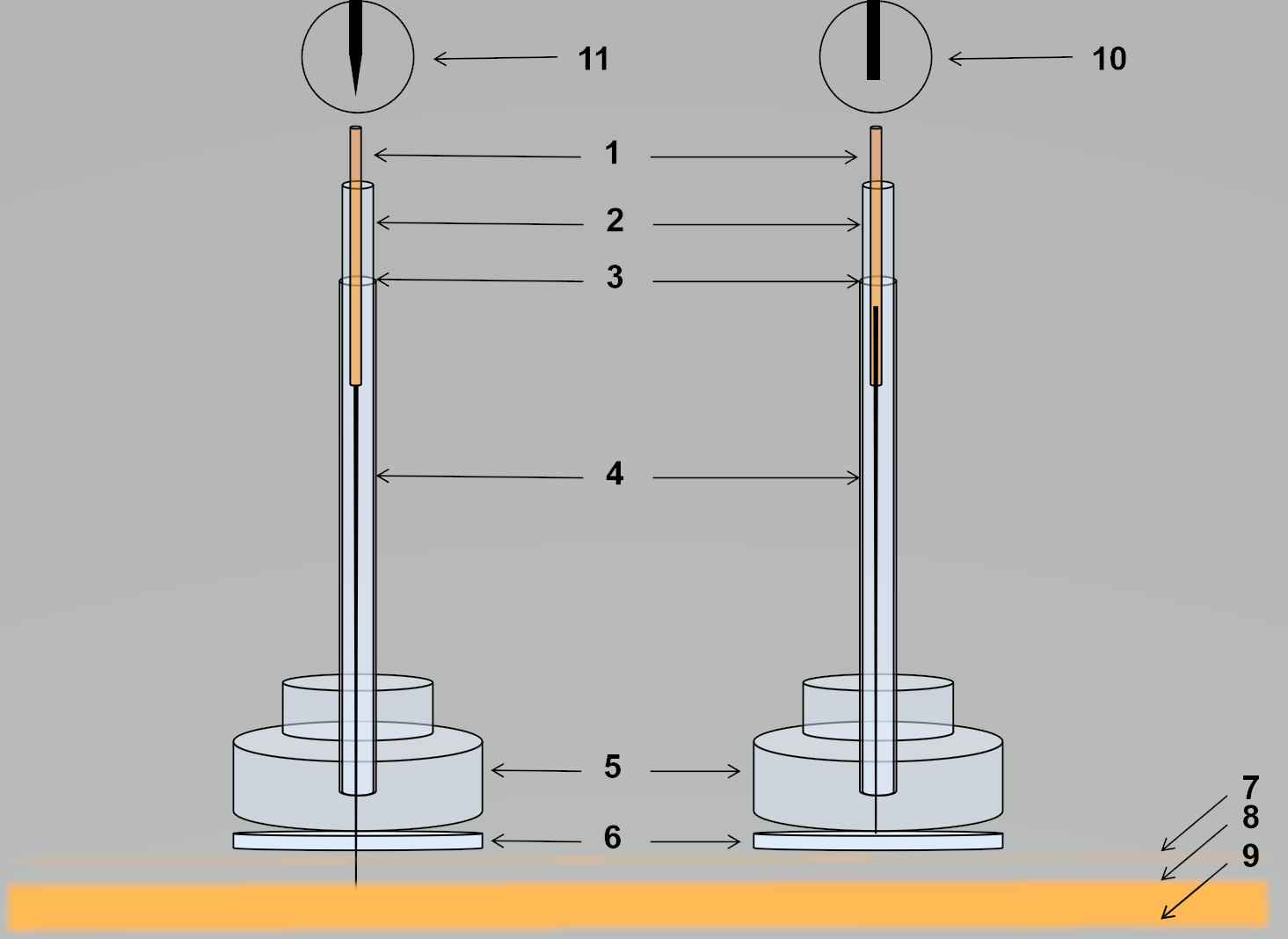


**Figure 3.** Normal guide device and Park sham device unit: 1. Needle handle, 2. Guide tube, 3. Guide o-ring, 4. Park tube, 5. Flange, 6. Adhesive pad, 7. Skin, 8. Dermis, 9. Muscle, 10. Blunt tip of sham needle, 11. Sharp tip of real needle.

In case of unacceptable pain, the patients will be allowed to take acute pain medications (nonsteroidal anti-inflammatory drugs, acetaminophen or aspirin) prescribed by our dentist. We will require that the same acute pain medication be used throughout the baseline period and the duration of the treatment period, which is documented throughout the course of the study. TMD-specific medication (corticosteroids, benzodiazepines, sedative hypnotics, muscle relaxants, opioids, antidepressants, and anticonvulsants) will be disallowed throughout the study.

**6. CONCOMITANT MEDICATIONS**

Prior and concomitant medication information will be collected for all participants for the 30 days prior to the screening and during the study. Information collected will include medication, total daily dose, start date, stop date (if applicable), and primary reason for use. Information about treatment with injection therapy, acupuncture, biofeedback, TENS, occlusal splint therapy, and TMD specific-medication will also be collected for all participants for the 30 days prior to the screening and during the study.

**7. STUDY VISIT PLAN**

The schedule of Events table, which provides an overview of the study phases, timeline, and events by visit, is provided in **Table 1**. In the following section, events are listed by study phase and visit, though the order of events listed within a visit does not imply the order in which the events will occur. A description of the procedures is provided in **Section 8**.

**7.1 Screening Period**

The screening period will be conducted 7 days before the baseline period. Signed informed consent will be obtained at the end of the screening period. Participant will be preliminary screened for inclusion and exclusion criteria during the screening period as well.

**7.2 Baseline Period**

At the beginning of a 7-day baseline period, the demography characteristics (sex, age, BMI, current employment status, education, marital status, and annual income), facial pain, and DC/TMD examination findings should be recorded.

The participant must be assisted with the provisioning and training of the TMD diary at the beginning of the baseline period. The TMD diary will be recorded daily during the baseline period. See **section 8.2.2** for further details on TMD diary. Participants compliance will be checked and at least 85% entries with the TMD diary should be attained. And an inclusion and exclusion criteria review based on TMD diary must be done at the end of baseline.

Each participant will also be provided with a participant identification card that states the participant identification number, the investigator’s name, and an emergency telephone number providing 24-hour service. The participant identification card should be returned to the investigator upon completion of the participant’s participation in the study.

At the allocation, participants will be randomly assigned (1:1) to either acupuncture group or sham acupuncture group. Additional examination of GCPS, JFLS-20, DASS-21, PSQI, PPT, and sEMG will be measured at the end of baseline. Laboratory tests, urine pregnancy test in female participants of childbearing potential vital signs, weight, and acupuncture expectancy scale will also be accessed at the end of baseline.

**7.3 Treatment Period**

Participants will receive acupuncture or sham acupuncture for twelve sessions (3 sessions per week, ideally every other weekday for 4 weeks) during the treatment period.

At the visits during the treatment period, the investigator will review medical history, record the change from baseline in mean weekly pain intensity at week 4 and record concomitant medications, specified concomitant therapies and AEs. Daily TMD diaries will be collected and compliance with diaries will be assessed. When participant has left, assess participant compliance with study therapy usage.

Additional assessments of the GCPS, JFLS-20, DASS-21, PSQI, PPT, and sEMG will also be accessed during treatment period.

Vital signs and weight will be recorded every week during the treatment period. The AEs will be monitored and recorded during the treatment period.

The patient satisfaction will be measured by Participants’ satisfaction scale at the end of treatment period. Patients will rate how they describe the satisfaction since beginning the treatment in this study on a 5-point scale where 1=extremely dissatisfied; 2=dissatisfied; 3=moderately satisfied; 4=satisfied; 5=extremely satisfied. Participants who achieve score 4 or score 5 are considered as responoders. Participants compliance will be checked every week during the treatment period and at least 50% entries with the TMD diary should be attained.

**7.4 Follow-up Period**

The follow-up period will investigate the long-term efficacy and provide safety evaluation. At the end of the follow-up period, GCPS, JFLS-20, DASS-21, PSQI are recorded. TMD Diaries will be recorded daily, and AEs will be monitored and recorded during the follow-up period. Vital signs and weight will also be accessed at the end of the follow-up period.

**8. ASSESSMENT**

**8.1 Screening and Baseline Procedures and Assessments**

**8.1.1 Demographics and Baseline Characteristics**

Prior to enrolling a patient in the study, the investigator must ascertain that the patient meets the selection criteria. The following assessments will be performed after the Informed Consent Form has been signed:

• Demographics (age, sex)

• Prior TMD treatment history

• Other recent medication

• Disease-specific history

• Relevant history (medical, psychiatric, neurological)

• Substance use

• Height and weight without shoes

• Signs and symptoms present at screening and/or baseline (before treatment start)

• Vital signs

• Blood and urine samples for screening and other clinical safety laboratory tests

**8.2 Efficacy Assessment**

**8.2.1 Use of Clinical Outcome Assessment**

The clinical outcome assessment and guidance will be given to the patients on how to complete them. The clinical outcome assessment will be administered in Chinese. All the following questionnaires are compared with the original text, and the Chinese version was confirmed without any addition or subtraction. The expression of each item is simple and easy to understand, and conforms to the expression habits of Chinese culture.

The following clinical outcome assessment will be used:

TMD diary – to assess daily TMDs, pain intensity and use of acute pain medications (**Appendix A** )

Facial Disability Index scale – to assess jaw opening and movement (**Appendix B)**

GCPS – to assess TMD-related pain and disability (**Appendix C)**

JFLS-20 – to assess TMD-related functional limitation (**Appendix D)**

DASS-21 – to measure depression, anxiety and stress (**Appendix E)**

PSQI – to measure sleeping quality (**Appendix F)**

PPT – to assess TMD-related pain threshold (**Appendix G)**

sEMG – to measure the state and function of the measured muscle (**Appendix H)**

Acupuncture Expectancy Scale – to measure the expectation of acupuncture (**Appendix I)**

Participant’s compliance – to measure the patient compliance (**Appendix J)**

Participant’s satisfaction scale – to assess patient satisfaction (**Appendix K)**

Blinding assessment – to test the success of blinding (**Appendix L)**

**8.2.2 TMD Diary**

The patient will be instructed to complete a TMD diary daily from baseline until completion. Eligible patients will receive comprehensive training from investigators on the use of the TMD diary. Investigators will also instruct patients on the requirement for timely and daily completion of the TMD diary.

The day of TMD diary distribution will start the 7-day TMD diary baseline period during which the patient will record daily information regarding pain severity, and intake of pain acute medication. Any patient found to be ineligible for the study during the baseline period or prior to randomization will not be randomized. On each day during the study until the completion/withdrawal, the patient will be asked to record diary data for the previous 24-hour period. For each day, the patient should record if they experienced any pains. Patients will also record any medications (name of drug, number of tablets/capsules, and the dose in milligrams per tablet/capsule) taken on each day. At least 85% compliance of TMD diary is needed during the baseline period and 50% during the treatment and follow-up periods. And the compliance will be assessed every four weeks.

**8.2.3 Facial Disability Index (FDI)**

Evaluation and application of the assessment tools were performed by an examiner. The clinical examination followed all biosafety recommendations, and the entire evaluation lasted approximately 40 minutes in a room exclusively reserved for this purpose.

Pain-free opening and maximum unassisted opening are measured by the interincisal distance between the maxillary and mandibular reference teeth. In certain settings, repeating the test, if the pain-free opening if less than 30 mm, might be appropriate in order to assess for possible low-range outlying values. Ask the patient about any pain produced by measuring maximum unassisted jaw opening.

Maximum assisted jaw opening is examiner-based. The examiner uses moderate pressure, push the mouth open further, measures the interincisal distance between the maxillary and mandibular teeth. The examiner has the option of repeating the procedure if she/he believes the patient prematurely terminated the procedure or responded with resistance. If patient indicates to the examiner that the procedure should be stopped, (Yes) is endorsed. If the patient indicates for the procedure to be terminated simultaneous with the examiner’s percept that the assisted opening has reached its maximum, (No) should be endorsed. For all other situations, (No) should be endorsed.

Lateral excursive measurements are made between the maxillary and mandibular reference midlines, while protrusive excursive measurement is made between the labial surfaces of the maxillary and mandibular reference teeth. If the patient cannot perform a movement, indicate this on the recording form by leaving the section blank. For the lateral excursive movements, if the patient is confused about direction she/he should move his or her jaw, touch the ipsilateral side of the face, lip, or even shoulder, and ask the patient to move towards the indicated side. Ask patient to move the mandible forward. Record any reported pain. Note that if the mandibular incisors cannot be protruded beyond the maxillary incisors, the value will be negative. If the incisors exhibit a Class III (anterior cross-bite situation exists) situation in maximum closure, the horizontal overlap is recorded as a negative value. The protrusive movement measured as the distance from the labial surfaces of the maxillary to mandibular incisors, will be recorded as a positive number.

**8.2.4 Graded Chronic Pain Scale (GCPS)**

This scale captures disability, pain intensity (high and low impact), and whether an individual is able to work due to pain.^26^ GCPS includes, in addition to the 3 items for pain intensity and 4 items for function, one item for number of days of pain. Each item has a score of 0-10. Characteristic Pain Intensity (CPI): compute mean of items 2-4 (pain right now, worst pain, average pain), and multiply by 10. Interference Score: compute mean of items 6-8 (daily activities, social activities, work activities), and multiply by 10. Disability score for number of days with interference: assign points based on below table. Disability score for the interference score: assign points based on **Table 4**.

**Table 4. Rating scale of disability points**

| **Points for Disability Days** | | **Points for Pain-related** | |
| --- | --- | --- | --- |
| 1 month（30 days） | | Interference Score | |
| Days | Points | Interference | Points |
| 0-1 | 0 | 0-29 | 0 |
| 2 | 1 | 30-49 | 1 |
| 3-5 | 2 | 50-69 | 2 |
| 6+ | 3 | 70+ | 3 |

The total Disability Score=Points for Disability Days + Points for Interference Score.

If one or more responses are missing among items 2-4 (pain intensity), the respective subscale should not be scored due to the broad scope that the three items cover. For the function items (6-8), one missing value may not represent the same information loss, and the subscale score could be computed albeit with decreased reliability. Missing data for number of disability days precludes determination of graded chronic pain status.

Determination of graded chronic pain status based on CPI and Disability Points. The Specific details based on **Table 5**.

**Table 5. Determination of chronic pain grade**

| **Grade** | **Label** | **CPI** | **Disability Points** |
| --- | --- | --- | --- |
| 0 | None | 0 | N/A |
| 1 | Low intensity pain, with none-low pain-related disability | <50 | <3 |
| 2 | High intensity pain, with none-low pain-related disability | ≥50 | <3 |
|  | [2a]High intensity pain, without pain-related disability | ≥50 | <3 |
|  | [2b]High intensity pain, with low pain-related disability | ≥50 | <3 |
| 3 | Moderately limiting | N/A | 3-4 |
| 4 | Severely limiting | N/A | 5-6 |

**8.2.5 Jaw Functional Limitations Scale-20 (JFLS-20)**

The JFLS-20 is an organ-specific instrument comprising 3 constructs for assessing functional status of the masticatory system; the 3 scales exhibit properties that are ideal for both research and patient evaluation in patient groups with a range of functional limitations of the jaw.^27^ The 20-item JFLS covers activities involving social aspects such as facial expressions (happy and angry), kissing, singing, frowning, laughing, and other jaw activities such as chewing a hard bread, chewing crackers, eating soft food that requires no chewing, opening the mouth wide to bite an apple or a sandwich, and talking. A response scale used 0 to 10 signifying degree of limitation (0=no limitation and 10=severe limitation); an option for “not applicable” is also available, and is scored as “missing.” The subscales are computed as the mean response for all items in the subscale. Mastication is based on 6 items, vertical jaw mobility is based on 4 items, and verbal and emotional expression is based on 8 items; 2 items are not scored as part of these 3 subscales. A total score is also computed from the 3 subscales when all 3 component scores are available. The JFLS-20 is designed based on item response theory, and consequently identical non-zero responses to all items within a subscale are highly improbable; non-zero responses that are the same within a subscale were regarded as invalid and recoded to “missing.”

Scoring:

From either the short form (all items) or the long form (items 1, 3, 6, 10, 11, 12, 13, and 19), a single global score of “jaw functional limitation” can be computed as the mean of the available items. Subscale scores for each type of functional limitation are computed, as follows:

Mastication: mean of items 1-6.

Vertical jaw mobility: mean of items 7-10.

Verbal and emotional expression: mean of items 13-20.

A second type of global score can be obtained from the long form by computing the mean of the 3 subscale scores, as computed above. Note that all 3 subscale scores must be present in order to compute the global score in this manner.

For the JFLS-20, scores can be computed based on no more than the following number of items with missing response: short form, 2 items missing allowed; mastication, 2 items missing allowed; mobility, 1 item missing allowed; and communication, 2 items missing allowed. Norms have not yet been established for this instrument.

**8.2.6 Depression Anxiety Stress Scales-21 (DASS-21)**

The DASS is a set of three self-report scales designed to measure the negative emotional states of depression, anxiety and stress. The DASS was constructed not merely as another set of scales to measure conventionally defined emotional states, but to further the process of defining, understanding, and measuring the ubiquitous and clinically significant emotional states usually described as depression, anxiety and stress. Each of the three DASS scales contains 7 items, divided into subscales with similar content. The Depression scale (item 3,5,10,13,16,17,21) assesses dysphoria, hopelessness, devaluation of life, self-deprecation, lack of interest/involvement, anhedonia, and inertia. The Anxiety scale (item 2,4,7,9,15,19,20) assesses autonomic arousal, skeletal muscle effects, situational anxiety, and subjective experience of anxious affect. The Stress scale (item 1,6,8,11,12,14,18) is sensitive to levels of chronic non-specific arousal. It assesses difficulty relaxing, nervous arousal, and being easily upset/agitated, irritable/over-reactive and impatient. Subjects are asked to use 4-point severity/frequency scales to rate the extent to which they have experienced each state over the past week. Scores for Depression, Anxiety and Stress are calculated by summing the scores for the relevant items. To calculate comparable scores with full DASS, each seven-item scale was multiplied by two. Participants were asked to rate how many of each of the items (in the form of statements) applied to them over the past week, with 0=did not apply to me at all to 3=applied to me very much, or most of the time. The higher the score, the more severe the emotional distress was.

**8.2.7 Pittsburgh Sleep Quality Index (PSQI)**

PSQI aims to assess a person’s sleep quality with questions relating to their sleeping habits within the past month. The PSQI consists of 18 self-rated questions and five questions raised by the bedpartner or roommate **(Table 6)**. The latter five questions are used for clinical information only, are not tabulated in the scoring of the PSQI. These 19 items are grouped into seven component scores, each weighted equally on a 0-3 scale. Higher scores indicate worse sleep quality. These components are subjective sleep quality, sleep latency, sleep duration, habitual sleep efficiency, sleep disturbances, use of sleeping medications, and daytime dysfunction over the past month. The seven-component scores are added in order to obtain a “global” score ranging from 0 to 21 points, where “0” indicates no difficulty and “21” indicates major difficulties in all areas. The global PSQI score, ranging from 0 to 21, can be collected by summing the seven components after weighting them on a scale ranging from 0 to 3. For each component, as well as the global PSQI score, higher sleep scores show worse sleep quality. The global score < 5 associated with good sleep quality while > 5 associated with poor sleep quality.

**Table 6. Scoring rule of pittsburgh sleep quality index**

| **component1**  **PSQISLPQUAL** | **OVERALL SLEEP QUALITY** | **Q6**  **Minimum Score = 0 (better); Maximum Score = 3 (worse)** |
| --- | --- | --- |
| component2 PSQILATEN | SLEEP LATENCY | **First, recode Q2 into Q2new thusly:**  IF Q2 > 0 and < 15, THEN set value of Q2new to 0  IF Q2 > 15 and < 30, THEN set value of Q2new to 1  IF Q2 > 30 and < 60, THEN set value of Q2new to 2  IF Q2 > 60, THEN set value of Q2new to 3  **Next**  IF Q5a + Q2new = 0, THEN set value to 0  IF Q5a + Q2new > 1 and < 2, THEN set value to 1  IF Q5a + Q2new > 3 and < 4, THEN set value to 2  IF Q5a + Q2new > 5 and < 6, THEN set value to 3  Minimum Score = 0 (better);  Maximum Score = 3 (worse) |
| component3  PSQIDURAT | DURATION OF SLEEP | IF Q4 > 7, THEN set value to 0  IF Q4 < 7 and > 6, THEN set value to 1  IF Q4 < 6 and > 5, THEN set value to 2  IF Q4 < 5, THEN set value to 3  Minimum Score = 0 (better);  Maximum Score = 3 (worse) |
| component4  PSQIHSE | SLEEP EFFICIENCY | Diffsec = Difference in seconds between day and time of day Q1 and day Q3  Diffhour = Absolute value of diffsec / 3600  newtib =IF diffhour > 24, then newtib = diffhour – 24  IF diffhour < 24, THEN newtib = diffhour  (NOTE, THE ABOVE JUST CALCULATES THE HOURS BETWEEN GNT (Q1) AND GMT (Q3))  tmphse = (Q4 / newtib) * 100  IF tmphse > 85, THEN set value to 0  IF tmphse < 85 and > 75, THEN set value to 1  IF tmphse < 75 and > 65, THEN set value to 2  IF tmphse < 65, THEN set value to 3  Minimum Score = 0 (better); Maximum Score = 3 (worse) |
| component5  PSQIDISTB | SLEEP DISTURBANCE | IF Q5b + Q5c + Q5d + Q5e + Q5f + Q5g + Q5h + Q5i + Q5j (IF Q5JCOM is null or Q5j is null, set the value of Q5j to 0) = 0, THEN set value to 0  IF Q5b + Q5c + Q5d + Q5e + Q5f + Q5g + Q5h + Q5i + Q5j (IF Q5JCOM is null or Q5j is null, set the value of Q5j to 0) > 1 and < 9, THEN set value to 1  IF Q5b + Q5c + Q5d + Q5e + Q5f + Q5g + Q5h + Q5i + Q5j (IF Q5JCOM is null or Q5j is null, set the value of Q5j to 0) > 9 and < 18, THEN set value to 2  IF Q5b + Q5c + Q5d + Q5e + Q5f + Q5g + Q5h + Q5i + Q5j (IF Q5JCOM is null or Q5j is null, set the value of Q5j to 0) > 18, THEN set value to 3  Minimum Score = 0 (better);  Maximum Score = 3 (worse) |
| component6  PSQIMEDS | NEED MEDS TO SLEEP | Q7  Minimum Score = 0 (better);  Maximum Score = 3 (worse) |
| component7  PSQIDAYDYS | DAY DYSFUNCTION DUE TO SLEEPINESS | IF Q8 + Q9 = 0, THEN set value to 0  IF Q8 + Q9 > 1 and < 2, THEN set value to 1  IF Q8 + Q9 > 3 and < 4, THEN set value to 2  IF Q8 + Q9 > 5 and < 6, THEN set value to 3  Minimum Score = 0 (better);  Maximum Score = 3 (worse) |
| PSQI | TOTAL | DURAT + DISTB + LATEN + DAYDYS + HSE + SLPQUAL + MEDS  Minimum Score = 0 (better);  Maximum Score = 21 (worse)  Interpretation:  TOTAL < 5 associated with good sleep quality  TOTAL > 5 associated with poor sleep quality |

Note: PSQISLPQUAL=PSQI sleep quality; PSQILATEN=PSQI sleep latency; PSQIDURAT=PSQI duration of sleep; PSQIHSE=PSQI sleep efficiency; Diffsec=difference in seconds; Diffhour=Difference in hours; GNT=gone to bed at night time; GMT=get up in the morning time; tmphse= time of sleep efficiency; PSQIDISTB=PSQI sleep disturbance; PSQIMEDS=PSQI need medicine to sleep; PSQIDAYDYS=PSQI day dysfunction due to sleepiness; DURAT=duration; DISTB=disturbance; LATEN=latency; DAYDYS=day dysfunction; HSE=sleep efficiency; SLPQUAL=sleep quality; MEDS=medicine to sleep.

**8.2.8 Pressure Pain Threshold Measurements**

Pressure pain threshold (PPT) is the only test for deep-pain sensitivity, most probably mediated by muscle C- and A-delta fibers. PPT is defined as the amount of pressure at which the participant first perceived the stimulus to be painful. PPT is one of the commonly used evaluation indicators of TMD pain, which is used for quantitative description.

Trained study staff will perform the PPT measurements. An electronic pressure algometer (Somedic Sales AB, Solna, Stockholm, Sweden) will be used to assess the PPT. This device consists of a grip with a pressure-sensitive strain gauge at the tip and a display unit. The 1 cm^2^ probe tip is covered with a 1 mm thick rubber pad to minimize the risk of irritation of the skin. The algometer is held perpendicular to the skin surface over the muscles and the pressure is increased at a standardized rate of 50 kPa per second. The patient is positioned comfortably in a sitting position, with muscle relaxed. And the patients will be instructed to press a signal button when the sensation of “pressure” changed into “pain”. This was first performed over the soft tissue close to the base of the thumb on the dorsal side of the right hand, in order to accustom the subject to the procedure. The following sites are assessed: bilateral masseter muscles, bilateral anterior temporalis muscle, bilateral sternocleidomastoid, bilateral trapezius muscles and the bilateral temporomandibular joint. The values from the right and left sides will be averaged to obtain a single PPT value per anatomical site, and the average of three trials is considered the final PPT value. There is a two-minute interval between the trials at the same muscle site, and a five-second interval between the measurements of one muscle site and the other.^28^

**8.2.9 Surface Electromyography (sEMG)**

Surface Electromyography (sEMG) is a kind of examination that reflects the state and function of the measured muscle by recording changes in electrical signals during muscle activity. The electrical activity of the anterior part of the temporal and masseter muscles is assessed in accordance with the assumptions of surface electromyography (sEMG) .^29^

During the sEMG recording, the environment is kept quiet, with the subject seated in a comfortable chair with the soles of the feet flat on the ground with their arms resting on their legs. The head is positioned upright, with the patient looking toward the horizon. Prior to electrode placement, the subjects’ skin is cleaned with alcohol 75% solution, with the aim of eliminating any facial oils or pollution that could interrupt sEMG signals. Double surface electrodes of silver chloride, disposable, bipolar, with self-adhesive gel are placed on the masseter and anterior temporalis bilaterally, in the longitudinal direction of the muscle fibers. Disposable circular electrodes with an interelectrode distance of 20 mm and a ground electrode placed above the crease of the wrist.

Surface EMG was recorded using a Miotec^®^, model Miotool 400 4-channel system. The system acquired 14-bit EMG signals with electrical isolation of 3000 volts, high EMG signal representation across all channels (2000 samples/second per channel), rejection of 110 dB common mode and low noise level < 2 LSB (Low Significant Bit); EMG signals are acquired using Miograph software with 2000 Hz sampling frequency, 20-500 Hz bandpass filter with interference eliminated by the Notch Filter.

A muscle function test is performed before placing the electrodes served to identify the center of the muscles to be analyzed. The electrodes are placed parallel to the muscle origin and insertion. Before the exam, subjects are asked to perform a maximum voluntary contraction (MVC), a five-second isometric contraction of the masseter and anterior temporal muscles, in order to conduct normalization of the data, interpreted subsequently with the MATLAB. EMG signal acquisition are captured in the following three tasks: the mandibular resting position (MR), the habitual chewing (HC), and the maximum voluntary contraction (MVC). When measuring HC, Trident^®^ gum is used for 20 chewing cycles.^30 31^ Potential amplitude is expressed in microvolts (μV) and expressed by root mean square (RMS).

**8.2.10 Acupuncture Expectancy Scale**

The acupuncture expectancy scale consists of an item measuring the expectation of improvement of illness due to acupuncture treatment. Patients will be asked to rate from 1 to 5 on a five-point Likert scale, with 1 indicating “Ineffective”, 2 indicating “May be ineffective”, 3 indicating “Unclear”, 4 indicating “May be effective”, 5 indicating “Effective” with the expected improvement as result of acupuncture.

**8.2.11** **Patient’s satisfaction**

The Participants’ satisfaction scale is a validated generic tool for assessment of patient satisfaction. Patients will rate how they describe the satisfaction since beginning the treatment in this study on a 5-point scale where 1=extremely dissatisfied; 2=dissatisfied; 3=moderately satisfied; 4=satisfied; 5=extremely satisfied. Participants who achieve score 4 or score 5 are considered as responoders.

**8.2.12 Blinding assessment**

To test the success of blinding, within 5 minutes after treatment at week 4, participants will be told that there are two kinds of treatment groups: “Acupuncture group” and “Sham acupuncture group”, and they will be randomly assigned to either group at 50% chance respectively. Participants will then be asked to answer the question “Do you think which kind of treatment group you have participated in during the past weeks?”The participants will be able to choose one of the following options as the answer: “Acupuncture group”, “Sham acupuncture group” or “Did not know”.

The Bang blinding indices will be used to assess the success of blinding. The Bang blinding index for each group represents the proportion of participants making a correct treatment guess beyond chance; 0 represents perfect blinding, a positive index indicates a correct guess, and a negative index indicates a guess in the opposite direction.

**8.3 Order of Assessments**

The assessments should preferably be administered in the following order:

No study related activities must be conducted until after the applicable Informed Consent Form is signed and the schedule of assessments is summarized in the **Table 1**.

**Screening period:**

• Informed consent Form will be signed

• Eligibility criteria

**Baseline period:**

• Eligibility criteria

• Demography Characteristics

• Disease history of temporomandibular disorder

• The clinical outcome assessment must be completed at the patient’s convenience before or after assessment of vital signs, weight

• Patients must complete the daily TMD diary entries prior to treatment

• The clinical outcome assessment completed in the clinic must be done before the treatment, including FDI, GCPS, JFLS-20, DASS-21, PSQI, PPT, sEMG, and acupuncture expectancy scale

**Treatment period:**

• Patients must complete the daily TMD diary entries during treatment period

• The clinical outcome assessment completed in the clinic must be done during treatment period, including FDI, GCPS, JFLS-20, DASS-21, and PSQI

• Treatment of each group must be preceded by the assessment of vital signs, weight, concomitant medications. AEs will be checked after treatment

• Patient’s satisfaction will be assessed at week 4

• The assessment of blinding will be conducted at week 4

• Participant’s compliance will be ensured at week 4

**Follow-up period:**

• A compliance check of the TMD diary must be conducted and the patient must be assisted with re-training if necessary. See **section 8.2.2** for further details on the TMD Diary

• The patient will be interviewed at week 8 to collect TMD diary and relevant information (AEs and concomitant medication)

• The assessments of FDI, GCPS, JFLS-20, DASS-21, and PSQI will be conducted at week 8

• The vital signs, weight,will be administrated at week 8

**8.4** **Treatment** **Compliance**

The performance of study treatment by the participants will be monitored by the investigator. Treatment compliance verification should be documented in the CRFs. If compliance is considered poor, the participants should be counseled on the importance of performing the study treatment.

Acupuncture compliance will be assessed based on the percentage of participants who have received at least 10 sessions (12 sessions in total, compliance rates ≥ 80%) of acupuncture. Acupuncture compliance rate will be defined as received sessions ÷ recommended sessions × 100%.

**9. ADVERSE EVENTS**

**9.1 Definitions of Adverse Event**

The study has been designed to minimize risks. Participants will be monitored closely for AEs with frequent visits. Adverse events will be managed by the investigator depending on the nature of the event.

Examination and close follow-up of parameters capturing participants’ overall health will be collected on case report forms. These will be completed at every study visit, and data will be compiled into a prespecified format and reviewed by the investigator.

**9.1.1 Adverse Event Definitions**

AE is any untoward medical occurrence in a patient or clinical study patient administered a medical intervention and which does not necessarily have a causal relationship with this treatment. A new condition or the worsening of a pre-existing condition will be considered an AE. Stable chronic conditions that are present before study entry and do not worsen during this study will not be considered AEs.

An AE can therefore be any unfavorable and unintended sign (including clinically significant out-of-range values from relevant tests, such as vital signs, weight), symptom, or disease temporally associated with the use of a medical intervention, regardless of whether it is considered related to the medical intervention.

Accordingly, an AE can include any of the following:

• All AEs associated with acupuncture include broken needle, needle phobia, needling pain after treatment, numbness, intense pricking, pricking lasting more than half an hour (no matter how intense it is) after acupuncture, subcutaneous hematoma, bleeding, infection, abscess formation at the needling site, other discomfort induced by acupuncture (such as fatigue, drowsiness, nausea, vomiting, palpitation, dizziness, pain, loss of appetite, insomnia, etc.), and aggravation of existing symptoms, etc.

• intercurrent illnesses

• physical injuries

• events possibly related to concomitant medication

• significant worsening (change in nature, severity, or frequency) of the disease under study or other pre-existing conditions (Note: A condition recorded as pre-existing that is intermittently symptomatic and that occurs during this study should be recorded as an AE.)

• events occurring during diagnostic procedures of this study or during any follow-up period of this study

• laboratory or diagnostic test abnormalities that result in the withdrawal of the patient from the study, are associated with clinical signs and symptoms or a SAE, require medical treatment or further diagnostic work-up, or are considered by the investigator to be clinically significant (Note: Abnormal laboratory test results at the baseline period that preclude a patient from entering the study or receiving treatment are not considered AEs.)

• patients with elevated liver function tests

**9.1.2 Serious Adverse Event Definitions**

SAE is any adverse event that:

• results in death

• is life-threatening (this refers to an event in which the patient was at risk of death at the time of the event; it does not refer to an event that hypothetically might have caused death had it been more severe)

• requires inpatient hospitalization or prolongation of existing hospitalization

• results in persistent or significant disability/incapacity

• is a congenital anomaly/birth defect

• is medically important (this refers to an event that may not be immediately life-threatening or result in death or hospitalization, but may jeopardize the patient or may require intervention to prevent any of the SAEs defined above)

An AE that does not meet any of the criteria for seriousness listed above will be regarded as a non-serious AE.

**9.1.3 AE Assessment Definitions**

**Assessment of Intensity**

The investigator must assess the intensity of the AE using the following definitions, and record it on the Adverse Event Form:

• Mild – the AE causes minimal discomfort and does not interfere in a significant manner with the patient’s normal activities.

• Moderate – the AE is sufficiently uncomfortable to produce some impairment of the patient’s normal activities.

• Severe – the AE is incapacitating, preventing the patient from participating in the patient’s normal activities.

**Assessment of Causal Relationship**

The investigator must assess the causal relationship between the AE and the treatment using the following definitions, and record it on the Adverse Event Form and the Serious Adverse Event Form (if applicable):

• Probable – the AE has a strong temporal relationship to the treatment or recurs on rechallenge, and another etiology is unlikely or significantly less likely.

• Possible – the AE has a suggestive temporal relationship to the treatment, and an alternative etiology is equally or less likely.

• Not related – the AE has no temporal relationship to the treatment or is due to underlying/concurrent disorder or effect of another therapy (that is, there is no causal relationship between the treatment and the AE).

An AE is considered causally related to the use of the treatment when the causality assessment is probable or possible.

Assessment of Outcome

The investigator must assess the outcome of the AE using the following definitions, and record it on the Adverse Event Form and the Serious Adverse Event Form (if applicable):

• Recovered – the patient has recovered completely, and no symptoms remain.

• Recovering – the patient’s condition is improving, but symptoms still remain.

• Recovered with sequelae – the patient has recovered, but some symptoms remain (for example, the patient had a stroke and is functioning normally, but has some motor impairment).

• Not recovered – the patient’s condition has not improved, and the symptoms are unchanged (for example, an atrial fibrillation has become chronic).

• Death.

**9.2 Recording of Adverse Event**

AEs must be recorded on an Adverse Event Form through the entire study. The investigator must provide information on the AE, preferably with a diagnosis, or at least with signs and symptoms; start and stop dates (and start and stop time if the AE lasts less than 24 hours); intensity; causal relationship to the treatment; action taken; and outcome. If the AE is not related to the treatment, an alternative aetiology must be recorded, if available. If the intensity changes during the course of the AE, this must be recorded on the Adverse Event Form. At each contact with the patient, the investigator must question the patient about AEs by asking an open-ended question such as, “Have you had any unusual symptoms or medical problems since the last visit? If yes, please describe.”

If the AE is serious, this must be indicated on the Adverse Event Form. Furthermore, the investigator must fill out a Serious Adverse Event Form and report the SAE to the principal investigator immediately (within 24 hours) after becoming aware of it (see **section 9.3**).

If individual AEs are later linked to a specific diagnosis, the diagnosis should be reported and linked to the previously reported AEs.

The investigator does not need to actively monitor patients for AEs once the study has ended. SAEs occurring after the defined study period should be reported to the principal investigator if the investigator becomes aware of them, following the procedures described in **section 9.3**.

**9.3 Reporting of Serious Adverse Event**

The investigator must report SAEs to the principal investigator immediately (within 24 hours) after becoming aware of them by completing a Serious Adverse Event Form. The initial Serious Adverse Event Form must contain as much information as possible and, if more information about the patient’s condition becomes available, the Serious Adverse Event Form must be updated with the additional information.

It is the investigator’s responsibility to be familiar with requirements regarding reporting SAEs to the Research Ethical Committee and to act accordingly. The principal investigator will assume responsibility for reporting SUSARs (suspected, unexpected, serious, adverse reactions) to the authorities in accordance with requirements and Research Ethical Committee. The principal investigator will assess the expectedness of SAEs and inform the investigators about SUSARs in the blinded SUSAR listings.

**9.4 Treatment and Follow-up Visits of Adverse Events**

Each patient is free to withdraw from the study at any time. The investigator can assess the subject's deterioration or AEs and determine whether the withdrawal from the trial is in the subject's best interest. Should a patient decide to withdraw after the administration of treatment, or should the investigator decide to withdraw the patient, all efforts will be made to complete and report all observations up to the time of withdrawal. A complete final evaluation at the time of the patient’s withdrawal should be made and an explanation given as to why the patient is withdrawing or being withdrawn from the study. And patients with AEs must be treated in accordance with usual clinical practice at the discretion of the investigator.

Non-serious AEs must be followed up until resolution or the completion/withdrawal visit, whichever comes first. At the completion/withdrawal visit, information on new AEs, if any, and stop dates for previously reported AEs must be recorded. SAEs that are spontaneously reported by a patient to the investigator after the completion/withdrawal visit must be handled in the same manner as SAEs that occur during the study. These SAEs will be recorded in the CRFs. The investigator must follow up on all SAEs until the event has resolved or stabilized, until the patient is referred to the care of a health care professional, or until a determination of a cause unrelated to the treatment or study procedure is made, and report to the principal investigator all relevant new information using the same procedures and timelines as those for the initial Serious Adverse Event Form.

Patients with a clinically significant out-of-range clinical safety laboratory test value at the completion/withdrawal visit must be followed in accordance with usual clinical practice. If the clinically significant out-of-range clinical safety laboratory test value has not normalized or stabilized or a diagnosis or a reasonable explanation has not been established by the investigator at the completion/withdrawal visit, the investigator must decide whether further follow-up visits are required (this may include an additional medical examination and/or additional blood sampling). If further follow-up visits are made, these must be documented in the patient’s medical records and not in the CRF.

**9.5 Pregnancy**

Although not necessarily considered as an AE, pregnancy in a patient in the study must be recorded on an Adverse Event Form, even if no AE associated with the pregnancy has occurred. Pregnancies must be reported to the principal investigator the same as SAEs. Any female patient becoming pregnant during the study will discontinue the treatment. All patients who become pregnant will be monitored for the outcome of the pregnancy (including spontaneous or voluntary termination).

An uncomplicated pregnancy should not be reported as an SAE; hospitalization for a normal birth should not be reported as an SAE. If, however, the pregnancy is associated with an SAE, the appropriate serious criterion must be indicated on the Serious Adverse Event Form. Examples of pregnancies to be reported as SAEs (medically important) are spontaneous abortions, stillbirths, and malformations.

**9.6 Clinical Safety Laboratory Tests**

The clinical laboratory tests will include a blood routine test, blood biochemistry test, blood coagulation test, urinalysis test, and a urine pregnancy test. The blood sampling and handling procedures are described in the study-specific Laboratory Specification Manual. The blood samples will be analyzed at the laboratory.

The investigator must review (initial and date) the results of the clinical safety laboratory tests as soon as possible after receipt of those results. Out-of-range values must be interpreted by the investigator as “not clinically significant” or “clinically significant” with a comment concerning the planned follow-up. Tests for clinically significant out-of-range values must be repeated, or an appropriate clinical follow-up must be arranged by the investigator and documented on the laboratory report until the value has stabilized or until the value has returned to a clinically acceptable value (regardless of the relationship to treatment). A patient with a value that is out-of-range at the completion or withdrawal visit and considered clinically significant must be followed in accordance with usual clinical practice until the value normalizes or stabilizes or a diagnosis or reasonable explanation has been established. Any out-of-range values followed after the last protocol-specified contact with the patient will be documented in the patient’s medical records.

Any out-of-range clinical safety laboratory test value considered clinically significant by the investigator must be recorded as an AE on an Adverse Event Form.

The laboratory will be notified by the principal investigator when the biological samples may be destroyed.

**9.7 Vital Signs**

Pulse rate, respiratory rate, and blood pressure will be measured by investigator using a standard digital meter. Pulse rate and blood pressure will be measured in the following order: supine, sitting, and standing after the patient has rested in each position for at least three minutes. Pulse rate will be recorded before each blood pressure measurement. Respiratory rate will be measured by the number of breaths in half a minute multiplying the number by 2. For any abnormal vital sign finding, the measurement should be repeated as soon as possible.

Any out-of-range values considered clinically significant by the investigator must be recorded as an AE on an Adverse Event Form.

**9.8 Height and Weight**

The patient’s height will be measured. The patients will be weighed wearing light clothing and no shoes. A similar amount of clothing must be worn on each occasion.

Any weight change considered clinically significant by the investigator must be recorded as an AE on an Adverse Event Form.

**9.9 Management of Reactions to Treatment**

A medical emergency should be treated appropriately by the investigator using proper standard of care and according to the guidelines for that emergency condition. Emergency equipment and medication for the treatment of these potential AEs must be available for immediate use. Should a medical condition arise that the investigator believes is related to the treatment, clinical judgement should be used to provide the appropriate response, including the consideration of discontinuation of treatment.

**10. ETHICS**

**10.1** **Ethical** **Rationale**

The participants will be fully informed about the study, including the risks and benefits of their participation in the study. Based on data from the nonclinical and clinical studies, and in combination with the cautionary measures implemented in the study design, the risks for the participants are considered well controlled and balanced with the potential benefits of the treatment.

The participants may withdraw from the study at any time, for any reason, specified or unspecified and without penalty or loss of benefits to which the participant is otherwise entitled.

In accordance with “Good Clinical Practice” guidelines of the International Conference on Harmonization, qualified medical personnel will be readily available to advise on study-related medical questions. Medical monitoring will be performed throughout the study. Safety data will be reviewed regularly by the Research Ethical Committee of Beijing Hospital of Traditional Chinese Medicine, Capital Medical University to ensure that prompt action is taken, if needed.

In accordance with Good Clinical Practice (GCP), the investigator will be responsible for all study related medical decisions.

**10.2 Informed Consent**

The investigator should fully inform the participant of all pertinent aspects of the study, including the written information. All written and/or oral information about the study will be provided as nontechnical as practical and understood by the participants. The participants should be given ample time and opportunity to inquire about details of the study and to decide whether or not to participate in the study.

Written informed consent will be obtained from each participant before any study-specific procedures or assessments are done and after the aims, methods, anticipated benefits, and potential hazards are explained. The participant’s willingness to participate in the study will be documented in a consent form, which will be signed and personally dated by the participant and by the person who conducted the informed consent discussion. The investigator will keep the original consent forms, and copies will be given to the participants. It will also be explained to the participants that the participant is free to refuse entry into the study and free to withdraw from the study at any time without prejudice to future treatment. As the blood and urine sampling for clinical laboratory tests and analyzes is an integral part of this study, the main Informed Consent Form covers these analyzes.

The investigator must identify vulnerable participants, that is, participants whose willingness to participate in this study might be unduly influenced by the expectation, regardless of whether it is justified, of benefits associated with participation, or of a retaliatory response from senior members of a hierarchy in case of refusal to participate. Participants thus identified must be excluded from participation in the study.

The participants must be informed that persons authorized by the acupuncturist, the clinical research assistant, the statistician, and the principal investigator may view their medical records. The confidentiality of the participants will in all cases be respected.

The consent procedures described above will only be implemented if allowed by law and regulations and will only be initiated after approval by the relevant ethics committees.

**10.3 Personal Data Protection**

The investigator must ensure that the privacy of the participants, including their identity and all personal medical information, will be maintained at all times. In CRFs and other documents submitted to the principal investigator, participants will be identified not by their names, but by an identification code (i.e., identification number).

Personal medical information may be reviewed for the purpose of participants safety and/or verifying data in the source and transcribed onto the CRFs. This review may be conducted by the investigators. Personal medical information will always be treated as confidential.

The investigators are responsible for ensuring the privacy, health, and welfare of the participants during and after the study and must ensure that trained personnel are immediately available in the event of a medical emergency. The investigators must be familiar with the background to, and requirements of, the study and with the contents of the treatments and medications as described in the interventions.

**10.4 Research Ethics Committees**

Before this study starts, the protocol will be submitted to the Research Ethical Committee for review. As required, the study will not start before the Research Ethical Committee to give written approval or a favorable opinion.

**11. DATA HANDING AND RECORD KEEPING**

**11.1 Data Collection**

**11.1.1Electronic** **Case Report Forms (eCRFs)**

CRFs will be used to collect all the data related to the study. The baseline characteristics of patients will be recorded by our investigator during the baseline period in CRFs, and the data manager will review all data. Upon the completion of the treatment and follow-up periods, all patients’ data will be completed and recorded on the original CRFs, following which the data manager will check data sets to ensure accuracy. If any inconsistencies are noted, corrections will be made according to, and marked on, the original CRFs.

The eCRFs use third party database (Beijing LNKMED Tech Company Limited) to capture data via an online system on a computer. When the investigator enters data in the eCRF (ideally during the visit or as soon as possible [<3 days] thereafter), the data will be recorded electronically in the database, and all entries and modifications to the data will be logged in an audit trail. Access to the system will only be granted after appropriate and documented training. Written instructions for using the system will be provided along with the training.

Electronic signatures will be used where signatures are required on pages and/or visits. Automated data entry checks will be implemented where appropriate; other data will be reviewed and evaluated for accuracy by the principal investigator. All entries, corrections, and changes must be made by the investigator.

**11.1.2 Patient Binders**

A Patient Binder will be provided for each patient. The Patient Binder contains different types of source documents, organized by visit and type. A ballpoint pen with waterproof ink must be used to enter information in the Patient Binder. The Patient Binder also contains Serious Adverse Event Fallback Forms. These forms must be used when the eCRF cannot be accessed.

**11.2 Database Management and Quality Control**

Quality control will be applied to ensure that all data are reliable and have been processed correctly, both during and after the trial. After the data is entered in the eCRFs, the principal investigator’s designee must perform source data verification check systematically for accuracy, consistency, completeness, and reliability, including a comparison of the data in eCRFs with source documents. Data should only be included in the final analysis when checks have been satisfactorily completed. If data does not pass validation rules, data queries will be addressed to the investigator to request clarification or correction. The investigator is obliged to respond by confirming or modifying the data questioned.

**11.3 Retention of Study Documents**

**Principal Investigator Responsibilities**

The principal investigator will have final responsibility for the processing and quality control of the data. Data management oversight will be carried out as described in the standard operating procedures (SOPs) for clinical studies. These SOPs will be reviewed by the principal investigator before the start of data management activities. The original CRFs will be archived by the principal investigator.

**Investigator Responsibilities**

The investigator must maintain all written and electronic records, accounts, notes, reports, and data related to the study and any additional records required to be maintained, including, but not limited to, the following: full case histories, signed informed consent forms, patient identification lists, CRFs for each patient on a per-visit basis, data results from other sources (e.g., TMD diary data), safety reports, reports of receipt, use. The investigator will retain all records related to the study until principal investigator sends written notification that records may be destroyed.

All paper research-related documents will be maintained in a closed file cabinet. All research materials, including hard copies and digital data, will be kept on file for at least five years following publication.

Before the study has been completed, the investigator can read and entry to the eCRF. After the study has been completed, all user access to the eCRF will be revoked. And the eCRF cannot be modified again.

At the end of the study, Beijing LNKMED Tech Company Limited will be provided with all data related to the study (including CRF data, queries, and the audit trail) using a secure electronic medium. When confirmation of receipt of the data has been received from Beijing LNKMED Tech Company Limited all user access to the CRF will be revoked. If, for some reason, the data are not readable for the full retention period, the investigator may request that the data be re-sent.

**12. STATISTICAL METHOLOGY**

A general description of the statistical methods is outlined below. A more detailed Statistical Analysis Plan will be provided in a separate document that will be finalized prior to database lock.

**12.1 Analysis Sets**

**12.1.1 Intention-to-Treat Analysis Set**

The Intention-to-Treat (ITT) analysis set will include all randomized participants. In this population, treatment will be assigned based on the treatment to which participants are randomized, regardless of which treatment they actually received.

**12.1.2 Per-Protocol (PP) Analysis Set**

The per-protocol (PP) analysis set was a subset of the ITT analysis set, including only participants who completed the study without any violations of the inclusion/exclusion criteria, or any deviations or omissions in the study treatment plan.

**12.1.3 Safety Analysis Set**

The safety analysis set will include all participants who receive at least one treatment. In this population, treatment will be assigned based upon the treatment participants actually receive, regardless of the treatment to which they are randomized.

**12.2 Sample Size and Power**

Based on our pilot study, the reduced pain intensity of TMD patients after 4 week treatment was 3.4±0.3 in the acupuncture group and 2.9±0.7 in the sham acupuncture group. A sample size of 50 patients gives at least 90% power for the study to succeed at an alpha level of 0.05. Assuming a 10% loss rate of follow-up, 30 patients per treatment group were planned for this study.

**12.3 Descriptive Statistics**

All summary statistics will be computed and displayed by treatment group. In general, summary statistics (count [n], mean, least-squares mean [LSM], standard deviation [SD], standard error [SE], median, lower and upper quartiles, minimum and maximum values) will be presented for continuous variables and counts and, if relevant, percentages will be presented for categorical variables. Graphical data displays may also be used as appropriate.

**12.4** **Participants Disposition**

Data from participants screened, participants screened but not randomized and reason not randomized, participants who are randomized (i.e., in the ITT set), participants randomized but not treated, participants in the safety and other analysis sets, participants who complete the study, and participants who withdraw from the study will be summarized using descriptive statistics. Data from participants who withdraw from the study will also be summarized by reason for withdrawal using descriptive statistics.

**12.5** **Demographics and Baseline Characteristics**

Participant demographics and baseline characteristics (sex, age, BMI, current employment status, education, marital status, and annual income, facial pain, DC/TMD examination findings) and baseline efficacy variables, will be summarized by treatment group using descriptive statistics.

**12.6 Efficacy analyzes**

**12.6.1 General Efficacy Analysis Methodology**

The ITT set (see **section 12.1.1**) will be used for all efficacy analyzes. Summaries will be presented by treatment group.

**12.6.2 Primary Analysis of the Primary Outcome**

The linear regression model will be applied for the primary outcome (change from baseline in mean weekly pain intensity at week 4). The model will include treatment as fixed effect; Sex, age, weekly pain intensity at baseline, TMD-specific medication as covariates. The LSM change from baseline with standard error (SE) was presented for each treatment group, and the LSM with 95% confidence intervals (95% CIs) for the treatment differences, and associated p-values will be provided.

### 12.6.3 Analysis of the Secondary Outcomes

For the proportion of responders of ≥30% and ≥50% reduction in weekly pain intensity, a logistic regression model was implemented adjusted for sex, age, weekly pain intensity at baseline, TMD-specific medication. Mean percentage with SE, and odds ratios with 95% CIs were presented for these efficacy outcomes by 4 weeks.

The analysis of continuous secondary outcomes were performed similarly to the primary efficacy outcome. A linear regression method, which is similar to the primary analysis setup, will be used for the analysis of the mean change from baseline in jaw opening and movement, GCPS, JFLS-20, DASS-21, PSQI, PPT, and sEMG at week 4. The LSM change from baseline with SE was presented for each treatment group, and the LSM with 95% CIs for the treatment differences, and associated p-values will be provided.

### 12.6.4 Analysis of the Exploratory Outcomes

The analysis of change from baseline in weekly pain intensity at week 8 were performed similarly to the primary efficacy outcome, and the proportion of responders of ≥30% and ≥50% reduction in weekly pain intensity, the jaw opening and movement, GCPS, JFLS-20, DASS-21, PSQI at week 8 were performed similarly to the secondary efficacy outcome.

**12.7 Safety analyzes**

The safety population will be used for all safety analyzes. Summaries will be presented by treatment group unless specified otherwise.

### 12.7.1 Acupuncture Administration

Number (%) of patient receiving each session of acupuncture will be summarized using descriptive statistics by treatment group.

### 12.7.2 Adverse Events

The incidence of AE and severity of the AE will be summarized using descriptive statistics. Each patient will be counted only once by using the AEs with the highest severity within each category. Treatment-related AE summaries will include AEs related to acupuncture.

Listings for SAEs, AEs will be presented. All information pertaining to AEs noted during the study will be listed by subject, detailing verbatim given by the investigator, date of onset, date of resolution, severity, and relationship to treatment. The onset of AEs will also be shown relative (in number of days) to the first day of treatment. In addition, AE descriptions, and AE by patient number and treatment group will be presented.

### 12.7.3 Clinical Laboratory Tests/ Vital Signs/ Weight

The incidence of potentially clinically significant abnormal results will be summarized using descriptive statistics. Listings of patients who have potentially clinically significant abnormal data will be presented.

**13. MONITORING PROCEDURES**

**13.1 Procedures for Monitoring Participant Compliance**

The investigator will be responsible for monitoring participant compliance (acupuncture sessions). If the principal investigator determines that the participant is not in compliance with the study protocol, the principal investigator must inform the Research Ethical Committee and they must review, discuss, and document the implications of the deviation.

**13.2 Study Monitoring**

To ensure compliance with GCP guidelines, the study monitor is responsible for ensuring that participants have signed the informed consent form and the study is conducted according to applicable SOPs, the protocol, and other written instructions and regulatory guidelines.

The main responsibilities of the study monitor are to visit the investigator before, during, and after the study to ensure adherence to the protocol, that all data are correctly and completely recorded and reported, and that informed consent is obtained and recorded for all participants before they participate in the study and when changes to the consent form are warranted, in accordance with Research Ethical Committee approvals.

The study monitor will be permitted to check and verify the various records (CRFs and other pertinent source data records, including source documentation) relating to the study to verify adherence to the protocol and to ensure the completeness, consistency, and accuracy of the data being recorded.

The investigator must agree to cooperate with the study monitor to resolve any problems, errors, or possible misunderstandings concerning the findings detected in the course of these monitoring visits and/or provided in follow-up written communication.

1. **STUDY DISCONTINUATION**

The investigators may choose to discontinue study treatment, if they think the continuation would be detrimental to the participants’ well-being. Whenever possible, the participants should be re-challenged with study treatment if their conditions were considered appropriate by the investigator. Participants also have the right to discontinue study treatment at any time for any reason, without prejudice to further treatment. Such participants will always be asked about the primary reason for their decision to discontinue study treatment and the presence of adverse events if any. If the discontinuation was due to adverse events, refer to the **section 9.** It should be evaluated if the discontinuation can be made temporarily, and permanent discontinuation should be the last choice.

It is essential to collect as much data as possible for all participants throughout the study, especially all potential endpoint events. Discontinuation of study treatment does not mean discontinuation of follow-up or termination of study participation. Participants who have discontinued performance of study treatment are expected to, and should be encouraged to, remain in follow-up until the end of study.

**15. STUDY ORGANISATION**

**15.1 Steering Committee**

The steering committee will make ethical, scientific, and strategic decisions regarding the overall conduct of the trial, to ensure the study execution is of the highest quality. It will perform logistical coordination of different committees and will meet regularly to review the study progress. It will also review and approve the reporting and publications of the study.

Steering committee members: Bin Li (Chair), Lu Liu, Qiuyi Chen, Tianli Lyn, Luopeng Zhao, Qiuyu Xia.

**15.2 Executive Committee**

The executive committee will help the steering committee maintain a high level of ethical, scientific, technical, and regulatory quality in all aspects of the trial. It will lead the successful implementation of the protocol according to the decisions made by the steering committee, and will monitor recruitment, compliance, and the adjudication process. It will meet more regularly to provide guidance for the day-to-day operations of the trial.

Executive committee members: Bin Li (Chair), Jing Guo, Huilin Liu, Jingqing Sun, Guiling Wang.

**15.3 Data Monitoring Committee**

The data monitoring committee will meet periodically to monitor the progress of all aspects of the trial and ensure that the trial meets the highest standards of ethics and patient safety. The members may suggest trial amendments regarding the safety of patients or early trial termination, but the final decision rests with the steering committee. Members of the data monitoring committee will not participate in the trial.

Data monitoring committee members: Shaosong Wang, Peng Chen, Xuefei Wang, Xu Ji.

**16. PUBLICATIONS**

Principal investigator has ownership of all data and results collected during this trial. All decisions regarding the use of data and results for public presentations and publications must be approved by principal investigator.

All presentations and publications of the results will be based on clean, checked, and validated data in order to ensure the accuracy of the results. The results of this trial will be published irrespective of whether the results are regarded positive or negative.

**17. FUNDING**

This study was funded by Beijing Traditional Chinese Medicine Science and Technology Project (JJ2018-53).

**18. SUMMARY OF CHANGES TO PROTOCOL**

**Protocol Changes Version 1.0 (Oct. 17, 2018) to 1.1 (Mar. 5, 2019)**

- Exclusion criteria

Patients received concomitant therapy for managing facial pain is added.

- Study design

The screening period is added.

- Secondary Outcomes

30% and 50% reduction in mean weekly pain intensity are added.

The jaw opening and movement is added.

- Exploratory Outcomes

Secondary outcomes during weeks 5-8 are retitled as exploratory outcomes.

**APPENDIX**

**APPENDIX A. TMD Diary**

| **Study Period**  （Please draw √ inside the present period brackets） | **Baseline period**  (-1-0 Week)  ( ) | **Treatment period**  (1-4 Week)  ( ) | **Follow-up period**  (5-8 Week)  ( ) |
| --- | --- | --- | --- |

**Subject Diary Card - TMD diary**

**Random Number：_____________________________**

**Time of This Visit：___________________(yy/mm/dd)**

**[Filling Explanation]**

Your TMD diary is used to record all the symptoms associated with your TMD pain. Please try your best to answer these questions on the day of TMD pain or the day after TMD pain. You need to record all symptoms you observe. Please try your best to ensure accuracy and completeness.

**Your charge doctor:**

**Tel of your charge doctor：**

**Explanation 1:** Please record a Pain diary **at 19:00** every day and recall your TMD pain from 19:00 yesterday to now **without blank**. If you have a TMD pain, please truthfully record it in the diary, and be sure to record all the details. And remember to **bring back** this TMD diary on each subsequent visit. The following are the problems you may encounter when filling. If you have any questions during the filling process, please contact your doctor in charge.

**Explanation 2：VAS score:**

Suppose 0 is no pain and 10 is the worst pain you can imagine in the world.

**VAS score:** please read the severity of your TMD pain in the position:

No pain└──┴──┴──┴──┴──┴──┴──┴──┴──┴──┘the worst pain

0 1 2 3 4 5 6 7 8 9 10

**Explanation 3：**Please fill in the time in **24 hours format**: year, month, day, hour.

**Explanation 4**：The dose and frequency should be filled in the record of medication intake, ? tablet/time, ? time/day.

| **TMD diary table** | | | | | | | | |
| --- | --- | --- | --- | --- | --- | --- | --- | --- |
| **Date**  **Symptom** | |  |  |  |  |  |  |  |
| **TMD pain duration** | **Start time** |  |  |  |  |  |  |  |
|  | **End time** |  |  |  |  |  |  |  |
| **VAS** | **0-10***  **(Explanation 2)** |  |  |  |  |  |  |  |
| **Acute medicine intake** | **Name** |  |  |  |  |  |  |  |
|  | **Dose and frequency** |  |  |  |  |  |  |  |
|  | **TMD pain after taking acute medicine** | □disappear  □remission  □no change | □disappear  □remission  □no change | □disappear  □remission  □no change | □disappear  □remission  □no change | □disappear  □remission  □no change | □disappear  □remission  □no change | □disappear  □remission  □no change |
| **Adverse event** | |  |  |  |  |  |  |  |

**APPENDIX B. Facial Disability Index scale (FDI)**

| **DC/TMD** **Examination** **Form** | | | | | | | | | | | | | | | | | | | | | | | | | | | | | | | | | | | | | | | | | | | | | |  | | Date filled out (mm-dd-yyyy) | | | | | | | | | | | | | | | | | | | | | | | | | | | | | | | | | | | | | | |  |  |
| --- | --- | --- | --- | --- | --- | --- | --- | --- | --- | --- | --- | --- | --- | --- | --- | --- | --- | --- | --- | --- | --- | --- | --- | --- | --- | --- | --- | --- | --- | --- | --- | --- | --- | --- | --- | --- | --- | --- | --- | --- | --- | --- | --- | --- | --- | --- | --- | --- | --- | --- | --- | --- | --- | --- | --- | --- | --- | --- | --- | --- | --- | --- | --- | --- | --- | --- | --- | --- | --- | --- | --- | --- | --- | --- | --- | --- | --- | --- | --- | --- | --- | --- | --- | --- | --- | --- | --- | --- |
| Patient | | | | | | | | | | | | | Examiner | | | | | | | | | | | | | | | | | | | | | | | | | | | | | | | | |  | | \|  \|  \| - \|  \|  \| - \|  \|  \|  \|  \| \| --- \| --- \| --- \| --- \| --- \| --- \| --- \| --- \| --- \| --- \| | | | | | | | | | | | | | | | | | | | | | | | | | | | | | | | | | | | | | | |  |  |
| **1a. Location of Pain: Last 30 days (Select all that apply)** | | | | | | | | | | | | | | | | | | | | | | | | | | | | | | | | | | | | | | | | | | | | | | | | | | | | | | | | | | | | | | | | | | | | | | | | | | | | | | | | | | | | | | | |  |
|  | **RIGHT PAIN** | | | | | | | | | | | | | | | | | | | | | | | | | | | | | | | | | | | | | | | | |  | | | | **LEFT PAIN** | | | | | | | | | | | | | | | | | | | | | | | | | | | | | | | | | | | | | | | | | |  |
|  | ◯None | | | ◯Temporalis  ◯Masseter | | | | | | | | | ◯Other m muscles  ◯TMJ | | | | | | | | | | | | | | | | | ◯Non-mast  structures | | | | | | | | | | | | ◯None | | | | | | | | | | ◯Temporalis  ◯Masseter | | | | | | | | | | | | ◯Other m muscles  ◯TMJ | | | | | | | | | | | | | | | ◯Non-mast  structures | | | | | | | | |  |
| **1b. Location of Headache: Last 30 days (Select all that apply)** | | | | | | | | | | | | | | | | | | | | | | | | | | | | | | | | | | | | | | | | | | | | | | | | | | | | | | | | | | | | | | | | | | | | | | | | | | | | | | | | | | | | | | | |  |
| ◯None | | | | | | | ◯Temporal | | | | | | | | | | | | | | | | | | | | ◯Other | | | | | | | | | | | | ◯None | | | | | | | | | | | | | | | | | | | | | | ◯Temporal | | | | | | | | | | | | | | | | ◯Other | | | | | | | | | | |  |
| **2. Incisal Relationships** | | | | | | | | | | | | **Reference tooth** | | | | | | | | | | | | | | | | | | | ○FDI #11 ○FDI #21 ○Other | | | | | | | | | | | | | | | | | | | | | | | | | | | | | | | | | | | | | | | | | | | | | | | | | | | | | | | | |  |
| Horizontal  Incisal Overjet | | | | ◯If negative | | | | | | | | \|  \|  \| \| --- \| --- \| | | | | | | mm | | | | | | Vertical  Incisal Overlap | | | | | | | | | | | | | | ◯If negative | | | | | | | | | | \|  \|  \| \| --- \| --- \| | | mm | | | | Midline  Deviation | | | | | | | | | | | | | | Right  ◯ | | | | Left  ◯ | | | | | N/A  ◯ | | | | | | \|  \|  \| \| --- \| --- \| | | | mm | |  |
| **3. Opening Pattern (Supplemental; Select all that apply)** | | | | | | | | | | | | | | | | | | | | | | | | | | | | | | | | | | | | | | | | | | | | | | | | Uncorrected Deviation | | | | | | | | | | | | | | | | | | | | | | | | | | | | | | | | | | | | | | | |  |
| ◯Straight | | | | | | | | | | | | | | | | | | | | | ◯Corrected deviation | | | | | | | | | | | | | | | | | | | | | | | | | | | | | | ◯Right | | | | | | | | ◯Left | | | | | | | | | | | | | | | | | | | | | | | | | | | | |  |
| **4.** **Opening** **Movements** | | | | | | | | | | | | | | | | | | | | | | | | | | | | | | | | | | | | | | | | | | | | | | | | | | | | | | | | | | | | | | | | | | | | | | | | | | | | | | | | | | | | | | | |  |
| A.pain Free Opening | | | | | | | | | | | | | |  | | | | | | | | | | | | | | | | | | | | | | | | | | | | | | | | | |  | | | | | | | | | | | | | | | | | | | | | | | | | | | | | | | | | | | | | | | |  |
| \|  \|  \| \| --- \| --- \| | | | | | mm | | | | | | | | | **RIGHT SIDE** | | | | | | | | | | | | | | | | | | | | | | | | | | | | | | | | | | | | |  | | **LEFT SIDE** | | | | | | | | | | | | | | | | | | | | | | | | | | | | | | | | | | |  |
|  |  |  |  |  |  |  |  |  |  |  |  |  |  |  | | | | | | | | | | | | | | Pain | | | | | | | | Familiar  Pain | | | | | | | | | Familiar  Headache | | | | | |  | |  | | | | | | | | | | | | | | | Pain | | | | | | Familiar  Pain | | | | | | | | | | Familiar  Headache | | | |  |
| B.Maximum Unassisted Opening | | | | | | | | | | | | | | Temporalis | | | | | | | | | | | | | | N Y | | | | | | | | N Y | | | | | | | | | N Y | | | | | |  | | Temporalis | | | | | | | | | | | | | | | N Y | | | | | | N Y | | | | | | | | | | N Y | | | |  |
| \|  \|  \| \| --- \| --- \| | | | | | mm | | | | | | | | | Masseter | | | | | | | | | | | | | | N Y | | | | | | | | N Y | | | | | | | | |  | | | | | |  | | Masseter | | | | | | | | | | | | | | | N Y | | | | | | N Y | | | | | | | | | |  | | | |  |
|  |  |  |  |  |  |  |  |  |  |  |  |  |  | TMJ | | | | | | | | | | | | | | N Y | | | | | | | | N Y | | | | | | | | |  | | | | | |  | | TMJ | | | | | | | | | | | | | | | N Y | | | | | | N Y | | | | | | | | | |  | | | |  |
|  | | | | |  | | | | | | | | | Other M Musc | | | | | | | | | | | | | | N Y | | | | | | | | N Y | | | | | | | | |  | | | | | |  | | Other M Musc | | | | | | | | | | | | | | | N Y | | | | | | N Y | | | | | | | | | |  | | | |  |
|  | | | | |  | | | | | | | | | Non-mast | | | | | | | | | | | | | | N Y | | | | | | | | N Y | | | | | | | | |  | | | | | |  | | Non-mast | | | | | | | | | | | | | | N Y | | | | | | | N Y | | | | | | | | | |  | | | |  |
|  | | | | | | | | | | | | | | | | | | | | | | | | | | | | | | | | | | | | | | | | | | | | | | | | | | | | | | | | | | | | | | | | | | | | | | | | | | | | | | | | | | | | | | | |  |
| C.Maximum Assisted Opening | | | | | | | | | | | | | Temporalis | | | | | | | | | | | | | | | N Y | | | | | | | | N Y | | | | | | | | | N Y | | | | | |  | | Temporalis | | | | | | | | | | | | | | N Y | | | | | | | N Y | | | | | | | | | | N Y | | | |  |
| \|  \|  \| \| --- \| --- \| | | | | | mm | | | | | | | | Masseter | | | | | | | | | | | | | | | N Y | | | | | | | | N Y | | | | | | | | |  | | | | | |  | | Masseter | | | | | | | | | | | | | | N Y | | | | | | | N Y | | | | | | | | | |  | | | |  |
|  |  |  |  |  |  |  |  |  |  |  |  |  | TMJ | | | | | | | | | | | | | | | N Y | | | | | | | | N Y | | | | | | | | |  | | | | | |  | | TMJ | | | | | | | | | | | | | | N Y | | | | | | | N Y | | | | | | | | | |  | | | |  |
|  |  |  |  |  |  |  |  |  |  |  |  |  | Other M Musc | | | | | | | | | | | | | | | N Y | | | | | | | | N Y | | | | | | | | |  | | | | | |  | | Other M Musc | | | | | | | | | | | | | | N Y | | | | | | | N Y | | | | | | | | | |  | | | |  |
| D.Terminate? | | | | | N Y | | | | | | | | Non-mast | | | | | | | | | | | | | | | N Y | | | | | | | | N Y | | | | | | | | |  | | | | | |  | | Non-mast | | | | | | | | | | | | | | N Y | | | | | | | N Y | | | | | | | | | |  | | | |  |
| **5. Lateral and Protrusive Movements** | | | | | | | | | | | | | | | | | | | | | | | | | | | | | | | | | | | | | | | | | | | | | | | | | | | | | | | | | | | | | | | | | | | | | | | | | | | | | | | | | | | | | | | |  |
|  | | | | |  | | | | | | | | **RIGHT SIDE** | | | | | | | | | | | | | | | | | | | | | | | | | | | | | | | | | | | | | |  | | **LEFT SIDE** | | | | | | | | | | | | | | | | | | | | | | | | | | | | | | | | | | |  |
|  | | | | |  | | | | | | | |  | | | | | | | | | | | | | | | Pain | | | | | | | | Familiar  Pain | | | | | | | | | Familiar  Headache | | | | | |  | |  | | | | | | | | | | | | | | Pain | | | | | | | Familiar  Pain | | | | | | | | | | Familiar  Headache | | | |  |
| A..Right Lateral | | | | | | | | | | | | | Temporalis | | | | | | | | | | | | | | | N Y | | | | | | | | N Y | | | | | | | | | N Y | | | | | |  | | Temporalis | | | | | | | | | | | | | | N Y | | | | | | | N Y | | | | | | | | | | N Y | | | |  |
| \|  \|  \| \| --- \| --- \| | | | | | mm | | | | | | | | Masseter | | | | | | | | | | | | | | | N Y | | | | | | | | N Y | | | | | | | | |  | | | | | |  | | Masseter | | | | | | | | | | | | | | N Y | | | | | | | N Y | | | | | | | | | |  | | | |  |
|  |  |  |  |  |  |  |  |  |  |  |  |  | TMJ | | | | | | | | | | | | | | | N Y | | | | | | | | N Y | | | | | | | | |  | | | | | |  | | TMJ | | | | | | | | | | | | | | N Y | | | | | | | N Y | | | | | | | | | |  | | | |  |
|  | | | | |  | | | | | | | | Other M Musc | | | | | | | | | | | | | | | N Y | | | | | | | | N Y | | | | | | | | |  | | | | | |  | | Other M Musc | | | | | | | | | | | | | | N Y | | | | | | | N Y | | | | | | | | | |  | | | |  |
|  | | | | |  | | | | | | | | Non-mast | | | | | | | | | | | | | | | N Y | | | | | | | | N Y | | | | | | | | |  | | | | | |  | | Non-mast | | | | | | | | | | | | | | N Y | | | | | | | N Y | | | | | | | | | |  | | | |  |
|  | | | | | | | | | | | | | | | | | | | | | | | | | | | | | | | | | | | | | | | | | | | | | | | | | | | | | | | | | | | | | | | | | | | | | | | | | | | | | | | | | | | | | | | |  |
| B.Left Lateral | | | | | | | | | | | | | Temporalis | | | | | | | | | | | | | | | N Y | | | | | | | | N Y | | | | | | | | | N Y | | | | | |  | | Temporalis | | | | | | | | | | | | | | N Y | | | | | | | N Y | | | | | | | | | | N Y | | | |  |
| \|  \|  \| \| --- \| --- \| | | | | | mm | | | | | | | | Masseter | | | | | | | | | | | | | | | N Y | | | | | | | | N Y | | | | | | | | |  | | | | | |  | | Masseter | | | | | | | | | | | | | | N Y | | | | | | | N Y | | | | | | | | | |  | | | |  |
|  |  |  |  |  |  |  |  |  |  |  |  |  | TMJ | | | | | | | | | | | | | | | N Y | | | | | | | | N Y | | | | | | | | |  | | | | | |  | | TMJ | | | | | | | | | | | | | | N Y | | | | | | | N Y | | | | | | | | | |  | | | |  |
|  | | | | |  | | | | | | | | Other M Musc | | | | | | | | | | | | | | | N Y | | | | | | | | N Y | | | | | | | | |  | | | | | |  | | Other M Musc | | | | | | | | | | | | | | N Y | | | | | | | N Y | | | | | | | | | |  | | | |  |
|  | | | | |  | | | | | | | | Non-mast | | | | | | | | | | | | | | | N Y | | | | | | | | N Y | | | | | | | | |  | | | | | |  | | Non-mast | | | | | | | | | | | | | | N Y | | | | | | | N Y | | | | | | | | | |  | | | |  |
|  | | | | | | | | | | | | | | | | | | | | | | | | | | | | | | | | | | | | | | | | | | | | | | | | | | | | | | | | | | | | | | | | | | | | | | | | | | | | | | | | | | | | | | | |  |
| C.Protrusion | | | | | | | | | | | | | Temporalis | | | | | | | | | | | | | | | N Y | | | | | | | | N Y | | | | | | | | | N Y | | | | | |  | | Temporalis | | | | | | | | | | | | | | N Y | | | | | | | N Y | | | | | | | | | | N Y | | | |  |
| \|  \|  \| \| --- \| --- \| | | | | | mm | | | | | | | | Masseter | | | | | | | | | | | | | | | N Y | | | | | | | | N Y | | | | | | | | |  | | | | | |  | | Masseter | | | | | | | | | | | | | | N Y | | | | | | | N Y | | | | | | | | | |  | | | |  |
|  |  |  |  |  |  |  |  |  |  |  |  |  | TMJ | | | | | | | | | | | | | | | N Y | | | | | | | | N Y | | | | | | | | |  | | | | | |  | | TMJ | | | | | | | | | | | | | | N Y | | | | | | | N Y | | | | | | | | | |  | | | |  |
|  | | | | |  | | | | | | | | Other M Musc | | | | | | | | | | | | | | | N Y | | | | | | | | N Y | | | | | | | | |  | | | | | |  | | Other M Musc | | | | | | | | | | | | | | N Y | | | | | | | N Y | | | | | | | | | |  | | | |  |
| ◯If negative | | | | |  | | | | | | | | Non-mast | | | | | | | | | | | | | | | N Y | | | | | | | | N Y | | | | | | | | |  | | | | | |  | | Non-mast | | | | | | | | | | | | | | N Y | | | | | | | N Y | | | | | | | | | |  | | | |  |
| **6. TMJ Noises During Open & Close Movements** | | | | | | | | | | | | | | | | | | | | | | | | | | | | | | | | | | | | | | | | | | | | | | | | | | | | | | | | | | | | | | | | | | | | | | | | | | | | | | | | | | | | | | | |  |
|  | **RIGHT TMJ** | | | | | | | | | | | | | | | | | | | | | | | | | | | | | | | | | | | | | | |  | | | **LEFT TMJ** | | | | | | | | | | | | | | | | | | | | | | | | | | | | | | | | | | | | | | | | | | | | | |
|  |  | | Examiner | | | | | | | | | | Patient | | | | | | | | | | Pain w/Click | | | | | | | | | Familiar  Pain | | | | | | | |  | | |  | | | | | | Examiner | | | | | | | | | | | | | | | | Patient | | | | | | | | Pain w/Click | | | | | | | | | Familiar  Pain | | | | | | |
|  |  | | Open | | | Close | | | | | | |  |  |  |  |  |  |  |  |  |  |  |  |  |  |  |  |  |  |  |  |  |  |  |  |  |  |  |  | | |  | | | | | | Open | | | | | | Close | | | | | | | | | |  |  |  |  |  |  |  |  |  |  |  |  |  |  |  |  |  |  |  |  |  |  |  |  |
|  | Click | | N Y | | | N Y | | | | | | | N Y | | | | | | | | | | N Y | | | | | | | | | N Y | | | | | | | |  | | | Click | | | | | | N Y | | | | | | N Y | | | | | | | | | | N Y | | | | | | | | N Y | | | | | | | | | N Y | | | | | |  |
|  | Crepitus | | N Y | | | N Y | | | | | | | N Y | | | | | | | | | |  | | | | | | | | |  | | | | | | | |  | | | Crepitus | | | | | | N Y | | | | | | N Y | | | | | | | | | | N Y | | | | | | | |  | | | | | | | | |  | | | | | |  |
| **7. TMJ Noises During Lateral & Protrusive Movements** | | | | | | | | | | | | | | | | | | | | | | | | | | | | | | | | | | | | |  | |  | | | | | | | |  | | | | | | | | | | | | |  | | | | | | | | | |  | | | | | | | | | | |  | | | | | | | |
|  | **RIGHT TMJ** | | | | | | | | | | | | | | | | | | | | | | | | | | | | | | | | | | | | | | |  | | | **LEFT TMJ** | | | | | | | | | | | | | | | | | | | | | | | | | | | | | | | | | | | | | | | | | | | | | |
|  |  | | Examiner | | | | | | | | | | Patient | | | | | | | | | | Pain w/Click | | | | | | | | | Familiar  Pain | | | | | | | |  | | |  | | | | | | Examiner | | | | | | | | | | | | | | | | Patient | | | | | | | | Pain w/Click | | | | | | | | | Familiar  Pain | | | | | | |
|  | Click | | N Y | | | | | | | | | | N Y | | | | | | | | | | N Y | | | | | | | | | N Y | | | | | | | |  | | | Click | | | | | | N Y | | | | | | | | | | | | | | | | N Y | | | | | | | | N Y | | | | | | | | | N Y | | | | | | |
|  | Crepitus | | N Y | | | | | | | | | | N Y | | | | | | | | | |  | | | | | | | | |  | | | | | | | |  | | | Crepitus | | | | | | N Y | | | | | | | | | | | | | | | | N Y | | | | | | | |  | | | | | | | | |  | | | | | | |
| **8. Joint Locking** | | | | | | | | | | | | | | | | | | | | | | | | | | | | | | | | | | | | | | | | | | | | | | | | | | | | | | | | | | | | | | | | | | | | | | | | | | | | | | | | |  |  |  |  |  |  |  |  |
|  | **RIGHT TMJ** | | | | | | | | | | | | | | | | | | | | | | | | | | | | | | | | | | | | | | |  | | | **LEFT TMJ** | | | | | | | | | | | | | | | | | | | | | | | | | | | | | | | | | | | | | | | | | | | | |  |
|  |  | | | | | | | Locking | | | | | | | | Reduction | | | | | | | | | | | | | | | | | |  | | | | | |  | | |  | | | | | | | | | | | | Locking | | | | | | | | | | | | Reduction | | | | | | | | | | | | | | | | | |  | | |  |
|  |  |  |  |  |  |  |  |  |  |  |  |  |  |  |  | Patient | | | | | | | | | Examiner | | | | | | | | |  | | | | | |  | | |  | | | | | | | | | | | |  |  |  |  |  |  |  |  |  |  |  |  | Patient | | | | | | | | | Examiner | | | | | | | | |  | | |  |
|  | While Opening | | | | | | | N Y | | | | | | | | N Y | | | | | | | | | N Y | | | | | | | | |  | | | | | |  | | | While Opening | | | | | | | | | | | | N Y | | | | | | | | | | | | N Y | | | | | | | | | N Y | | | | | | | | |  | | |  |
|  | Wide Open Position | | | | | | | N Y | | | | | | | | N Y | | | | | | | | | N Y | | | | | | | | |  | | | | | |  | | | Wide Open Position | | | | | | | | | | | | N Y | | | | | | | | | | | | N Y | | | | | | | | | N Y | | | | | | | | |  | | |  |
| **9. Muscle & TMJ Pain with Palpation** | | | | | | | | | | | | | | | | | | | | | | | | | | | | | | | | | | | | | | | | | | | | | | | | | | | | | | | | | | | | | | | | | | | | | | | | | | | | | | | | | | | | | | | |  |
|  | **RIGHT TMJ** | | | | | | | | | | | | | | | | | | | | | | | | | | | | | | | | | | | | | | |  | | | **LEFT TMJ** | | | | | | | | | | | | | | | | | | | | | | | | | | | | | | | | | | | | | | | | | | | | |  |
|  | | **(1kg)** | | | | | | | Pain | | | | | | Familiar  Pain | | | | | | | | | | | Familiar  Headache | | | | | | | | | Referred Pain | | | | | |  | | | **(1kg)** | | | | | | | | | | | | | Pain | | | | | | | | | Familiar  Pain | | | | | | | | | Familiar  Headache | | | | | | | | | | Referred Pain | | |  |
|  | | Temporalis (posterior) | | | | | | | N Y | | | | | | N Y | | | | | | | | | | | N Y | | | | | | | | | N Y | | | | | |  | | | Temporalis (posterior) | | | | | | | | | | | | | N Y | | | | | | | | | N Y | | | | | | | | | N Y | | | | | | | | | | N Y | | |  |
|  | | Temporalis (middle) | | | | | | | N Y | | | | | | N Y | | | | | | | | | | | N Y | | | | | | | | | N Y | | | | | |  | | | Temporalis (middle) | | | | | | | | | | | | | N Y | | | | | | | | | N Y | | | | | | | | | N Y | | | | | | | | | | N Y | | |  |
|  | | Temporalis (anterior) | | | | | | | N Y | | | | | | N Y | | | | | | | | | | | N Y | | | | | | | | | N Y | | | | | |  | | | Temporalis (anterior) | | | | | | | | | | | | | N Y | | | | | | | | | N Y | | | | | | | | | N Y | | | | | | | | | | N Y | | |  |
|  | | Masseter (origin) | | | | | | | N Y | | | | | | N Y | | | | | | | | | | |  | | | | | | | | | N Y | | | | | |  | | | Masseter (origin) | | | | | | | | | | | | | N Y | | | | | | | | | N Y | | | | | | | | |  | | | | | | | | | | N Y | | |  |
|  | | Masseter (body) | | | | | | | N Y | | | | | | N Y | | | | | | | | | | |  | | | | | | | | | N Y | | | | | |  | | | Masseter (body) | | | | | | | | | | | | | N Y | | | | | | | | | N Y | | | | | | | | |  | | | | | | | | | | N Y | | |  |
|  | | Masseter (insertion) | | | | | | | N Y | | | | | | N Y | | | | | | | | | | |  | | | | | | | | | N Y | | | | | |  | | | Masseter (insertion) | | | | | | | | | | | | | N Y | | | | | | | | | N Y | | | | | | | | |  | | | | | | | | | | N Y | | |  |
|  | | **TMJ** | | | | | | | | Pain | | | | | | | Familiar Pain | | | | | | | | | | | | Referred Pain | | | | | | | | | | | |  | | | **TMJ** | | | | | | | | | | | | | | | | | | Pain | | | | | | | Familiar Pain | | | | | | | | | Referred Pain | | | | | | | | | | |
|  | | Lateral pole **(0.5 kg)** | | | | | | | | N Y | | | | | | | N Y | | | | | | | | | | | | N Y | | | | | | | | | | | |  | | | Lateral pole **(0.5 kg)** | | | | | | | | | | | | | | | | | | N Y | | | | | | | N Y | | | | | | | | | N Y | | | | | | | | | | |
|  | | Around lateral pole **(1 kg)** | | | | | | | | N Y | | | | | | | N Y | | | | | | | | | | | | N Y | | | | | | | | | | | |  | | | Around lateral pole **(1 kg)** | | | | | | | | | | | | | | | | | | N Y | | | | | | | N Y | | | | | | | | | N Y | | | | | | | | | | |
| **10. Supplemental Muscle Pain with Palpation** | | | | | | | | | | | | | | | | | | | | | | | | | | | | | | | | | | | | | | | | | | | | | | | | | | | | | | | | | | | | | | | | | | | | | | | | | | | | | | | | | | | | | | | |  |
|  | | **RIGHT TMJ** | | | | | | | | | | | | | | | | | | | | | | | | | | | | | | | | | | | | | | |  | | | **LEFT TMJ** | | | | | | | | | | | | | | | | | | | | | | | | | | | | | | | | | | | | | | | | | | | | |
|  | | **(0.5kg)** | | | | | | | | | Pain | | | | | | | | | Familiar Pain | | | | | | | | | | | | | Referred Pain | | | | | | | |  | | |  | | | | | | | | | | | | | | | | | | | Pain | | | | | | | | Familiar Pain | | | | | | | | | Referred Pain | | | | | | | | |
|  | | Posterior mandibular region | | | | | | | | | N Y | | | | | | | | | N Y | | | | | | | | | | | | | N Y | | | | | | | |  | | | Posterior mandibular region | | | | | | | | | | | | | | | | | | | N Y | | | | | | | | N Y | | | | | | | | | N Y | | | | | | | | |
|  | | Submandibular region | | | | | | | | | N Y | | | | | | | | | N Y | | | | | | | | | | | | | N Y | | | | | | | |  | | | Submandibular region | | | | | | | | | | | | | | | | | | | N Y | | | | | | | | N Y | | | | | | | | | N Y | | | | | | | | |
|  | | Lateral pterygoid area | | | | | | | | | N Y | | | | | | | | | N Y | | | | | | | | | | | | | N Y | | | | | | | |  | | | Lateral pterygoid area | | | | | | | | | | | | | | | | | | | N Y | | | | | | | | N Y | | | | | | | | | N Y | | | | | | | | |
|  | | Temporalis tendon | | | | | | | | | N Y | | | | | | | | | N Y | | | | | | | | | | | | | N Y | | | | | | | |  | | | Temporalis tendon | | | | | | | | | | | | | | | | | | | N Y | | | | | | | | N Y | | | | | | | | | N Y | | | | | | | | |
| **11.Diagnoses** | | | | | | | | | | | | | | | | | | | | | | | | | | | | | | | | | | | | | | | | | | | | | | | | | | | | | | | | | | | | | | | | | | | | | | | | | | | | | | | | | | | | | | | |  |
|  | | Pain Disorders | | | | | | | | | | | | | | | | |  | | | Right TMJ Disorders | | | | | | | | | | | | | | | | | | | | | | | | | | | | | | | | | |  | | Left TMJ Disorders | | | | | | | | | | | | | | | | | | | | | | | | | | | | | |  |
|  | | ◯None | | | | | | | | | | | | | | | | |  | | | ◯None | | | | | | | | | | | | | | | | | | | | | | | | | | | | | | | | | |  | | ◯None | | | | | | | | | | | | | | | | | | | | | | | | | | | | | |  |
|  | | ◯Myalgia | | | | | | | | | | | | | | | | |  | | | Disc displacement (select one) | | | | | | | | | | | | | | | | | | | | | | | | | | | | | | | | | |  | | Disc displacement (select one) | | | | | | | | | | | | | | | | | | | | | | | | | | | | | |  |
|  | | ◯Myofascial pain with referral | | | | | | | | | | | | | | | | |  | | | ◯...with reduction | | | | | | | | | | | | | | | | | | | | | | | | | | | | | | | | | |  | | ◯...with reduction | | | | | | | | | | | | | | | | | | | | | | | | | | | | | |  |
|  | |  | | | | | | | | | | | | | | | | |  | | | ◯...with reduction, with intermittent locking | | | | | | | | | | | | | | | | | | | | | | | | | | | | | | | | | |  | | ◯...with reduction, with intermittent locking | | | | | | | | | | | | | | | | | | | | | | | | | | | | | |  |
|  | | ◯Right Arthralgia | | | | | | | | | | | | | | | | |  | | | ◯...without reduction, with limited opening | | | | | | | | | | | | | | | | | | | | | | | | | | | | | | | | | |  | | ◯...without reduction, with limited opening | | | | | | | | | | | | | | | | | | | | | | | | | | | | | |  |
|  | | ◯Left Arthralgia | | | | | | | | | | | | | | | | |  | | | ◯...without reduction, without limited opening | | | | | | | | | | | | | | | | | | | | | | | | | | | | | | | | | |  | | ◯...without reduction, without limited opening | | | | | | | | | | | | | | | | | | | | | | | | | | | | | |  |
|  | |  | | | | | | | | | | | | | | | | |  | | | ◯Degenerative joint disease | | | | | | | | | | | | | | | | | | | | | | | | | | | | | | | | | |  | | ◯Degenerative joint disease | | | | | | | | | | | | | | | | | | | | | | | | | | | | | |  |
|  | | ◯Headache attributed to TMD | | | | | | | | | | | | | | | | |  | | | ◯Subluxation | | | | | | | | | | | | | | | | | | | | | | | | | | | | | | | | | |  | | ◯Subluxation | | | | | | | | | | | | | | | | | | | | | | | | | | | | | |  |
| **12.Comments** | | | | | | | | | | | | | | | | | | | | | | | | | | | | | | | | | | | | | | | | | | | | | | | | | | | | | | | | | | | | | | | | | | | | | | | | | | | | | | | | | | | | | | | |  |
|  | | | | | | | | | | | | | | | | | | | | | | | | | | | | | | | | | | | | | | | | | | | | | | | | | | | | | | | | | | | | | | | | | | | | | | | | | | | | | | | | | | | | | | | |  |

**APPENDIX C. Graded Chronic Pain Scale** **Version 2.0**

1. On how many days in the last 6 months have you had facial pain?______Days
2. How would you rate your facial pain **RIGHT NOW**? Use a scale from 0 to 10, where 0 is "no pain"and 10 is "pain as bad as could be".

Pain as bad

No pain as could be

0 1 2 3 4 5 6 7 8 9 10

1. In the LAST 30 DAYS, how would you rate your **WORST** facial pain? Use the same scale, where 0 is "no pain" and 10 is "pain as bad as could be".

Pain as bad

No pain as could be

0 1 2 3 4 5 6 7 8 9 10

1. In the LAST 30 DAYS, **ON AVERAGE**, how would you rate your facial pain? Use the same scale where 0 is "no pain" and 10 is "pain as bad as could be". [That is, *your usual pain* at times you were in pain.]

Pain as bad

No pain as could be

0 1 2 3 4 5 6 7 8 9 10

1. In the LAST 30 DAYS, how many days did your facial pain keep you from doing your USUAL ACTIVITIES like work, school, or housework? (every day = 30 days)

____Days

1. In the LAST 30 DAYS, how much has facial pain interfered with your DAILY ACTIVITIES? Use a 0-10 scale, where 0 is "no interference" and 10 is "unable to carry on any activities".

Unable to carry

No interference on any activities

0 1 2 3 4 5 6 7 8 9 10

1. In the LAST 30 DAYS, how much has facial pain interfered with your **RECREATIONAL, SOCIAL AND FAMILY ACTIVITIES**? Use the same scale, where 0 is "no interference" and 10 is "unable to carry on any activities".

Unable to carry

No interference on any activities

0 1 2 3 4 5 6 7 8 9 10

1. In the LAST 30 DAYS, how much has facial pain interfered with your **ABILITY TO WORK**, including housework? Use the same scale, where 0 is "no interference" and 10 is "unable to carry on any activities".

Unable to carry

No interference on any activities

0 1 2 3 4 5 6 7 8 9 10

**APPENDIX D. Jaw Functional Limitation Scale-20**

For each of the items below, please indicate the level of limitation **during the last month**. If the activity has been completely avoided because it is too difficult, then circle '10'. If you avoid an activity for reasons other than pain or difficulty, leave the item blank.

|  |  | No  limitation | | | | | Severe  limitation | | | | | |
| --- | --- | --- | --- | --- | --- | --- | --- | --- | --- | --- | --- | --- |
| 1. | Chew tough food | 0 | 1 | 2 | 3 | 4 | 5 | 6 | 7 | 8 | 9 | 10 |
| 2. | Chew hard bread | 0 | 1 | 2 | 3 | 4 | 5 | 6 | 7 | 8 | 9 | 10 |
| 3. | Chew chicken (e.g., prepared in oven) | 0 | 1 | 2 | 3 | 4 | 5 | 6 | 7 | 8 | 9 | 10 |
| 4. | Chew crackers | 0 | 1 | 2 | 3 | 4 | 5 | 6 | 7 | 8 | 9 | 10 |
| 5. | Chew soft food (e.g., macaroni, canned or soft fruits, cooked vegetables, fish) | 0 | 1 | 2 | 3 | 4 | 5 | 6 | 7 | 8 | 9 | 10 |
| 6. | Eat soft food requiring no chewing (e.g.,mashed potatoes, apple sauce, pudding, pureed food) | 0 | 1 | 2 | 3 | 4 | 5 | 6 | 7 | 8 | 9 | 10 |
| 7. | Open wide enough to bite from a whole apple | 0 | 1 | 2 | 3 | 4 | 5 | 6 | 7 | 8 | 9 | 10 |
| 8. | Open wide enough to bite into a  sandwich | 0 | 1 | 2 | 3 | 4 | 5 | 6 | 7 | 8 | 9 | 10 |
| 9. | Open wide enough to talk | 0 | 1 | 2 | 3 | 4 | 5 | 6 | 7 | 8 | 9 | 10 |
| 10. | Open wide enough to drink from a cup | 0 | 1 | 2 | 3 | 4 | 5 | 6 | 7 | 8 | 9 | 10 |
| 11. | Swallow | 0 | 1 | 2 | 3 | 4 | 5 | 6 | 7 | 8 | 9 | 10 |
| 12. | Yawn | 0 | 1 | 2 | 3 | 4 | 5 | 6 | 7 | 8 | 9 | 10 |
| 13. | Talk | 0 | 1 | 2 | 3 | 4 | 5 | 6 | 7 | 8 | 9 | 10 |
| 14. | Sing | 0 | 1 | 2 | 3 | 4 | 5 | 6 | 7 | 8 | 9 | 10 |
| 15. | Putting on a happy face | 0 | 1 | 2 | 3 | 4 | 5 | 6 | 7 | 8 | 9 | 10 |
| 16. | Putting on an angry face | 0 | 1 | 2 | 3 | 4 | 5 | 6 | 7 | 8 | 9 | 10 |
| 17. | Frown | 0 | 1 | 2 | 3 | 4 | 5 | 6 | 7 | 8 | 9 | 10 |
| 18. | Kiss | 0 | 1 | 2 | 3 | 4 | 5 | 6 | 7 | 8 | 9 | 10 |
| 19. | Smile | 0 | 1 | 2 | 3 | 4 | 5 | 6 | 7 | 8 | 9 | 10 |
| 20 | Laugh | 0 | 1 | 2 | 3 | 4 | 5 | 6 | 7 | 8 | 9 | 10 |

**APPENDIX E. Depression Anxiety Stress Scale-21 (DASS-21)**

Please read each statement and circle a number 0, 1, 2 or 3 which indicates how much the statement applied to you over the past week. There are no right or wrong answers. Do not spend too much time on any statement.

The rating scale is as follows:

0 Did not apply to me at all

1 Applied to me to some degree, or some of the time

2 Applied to me to a considerable degree, or a good part of time

3 Applied to me very much, or most of the time

|  |  | Never | Sometimes | Often | Almost Always |
| --- | --- | --- | --- | --- | --- |
| 1 | I found it hard to wind down | 0 | 1 | 2 | 3 |
| 2 | I was aware of dryness of my mouth | 0 | 1 | 2 | 3 |
| 3 | I couldn’t seem to experience any positive feeling at all | 0 | 1 | 2 | 3 |
| 4 | I experienced breathing difficulty (e.g., excessively rapid breathing, breathlessness in the absence of physical exertion) | 0 | 1 | 2 | 3 |
| 5 | I found it difficult to work up the initiative to do things | 0 | 1 | 2 | 3 |
| 6 | I tended to over-react to situations | 0 | 1 | 2 | 3 |
| 7 | I experienced trembling (e.g., in the hands) | 0 | 1 | 2 | 3 |
| 8 | I felt that I was using a lot of nervous energy | 0 | 1 | 2 | 3 |
| 9 | I was worried about situations in which I might panic and make a fool of myself | 0 | 1 | 2 | 3 |
| 10 | I felt that I had nothing to look forward to | 0 | 1 | 2 | 3 |
| 11 | I found myself getting agitated | 0 | 1 | 2 | 3 |
| 12 | I found it difficult to relax | 0 | 1 | 2 | 3 |
| 13 | I felt down-hearted and blue | 0 | 1 | 2 | 3 |
| 14 | I was intolerant of anything that kept me from getting on with what I was doing | 0 | 1 | 2 | 3 |
| 15 | I felt I was close to panic | 0 | 1 | 2 | 3 |
| 16 | I was unable to become enthusiastic about anything | 0 | 1 | 2 | 3 |
| 17 | I felt I wasn’t worth much as a person | 0 | 1 | 2 | 3 |
| 18 | I felt that I was rather touchy | 0 | 1 | 2 | 3 |
| 19 | I was aware of the action of my heart in the absence of physical exertion (e.g.,sense of heart rate increase, heart missing a beat) | 0 | 1 | 2 | 3 |
| 20 | I felt scared without any good reason | 0 | 1 | 2 | 3 |
| 21 | I felt that life was meaningless | 0 | 1 | 2 | 3 |

**APPENDIX F. Pittsburgh Sleep Quality Index (PSQI)**

Instructions: The following questions relate to your usual sleep habits during the past month only. Your answers should indicate the most accurate reply for the majority of days and nights in the past month. Please answer all questions.

1. During the past month, what time have you usually gone to bed at night?

BED TIME ___________

2. During the past month, how long (in minutes) has it usually taken you to fall asleep each night?

NUMBER OF MINUTES___________

1. During the past month, what time have you usually gotten up in the morning?

GETTING UP TIME___________

4.During the past month, how many hours of actual sleep did you get at night? (This may be different than the number of hours you spent in bed.)

HOURS OF SLEEP PER NIGHT___________

**For each of the remaining questions, check the one best response. Please answer all questions.**

5. During the past month, how often have you had trouble sleeping because you.

a) Cannot get to sleep within 30 minutes

□None □Less than once a week

□Once or twice a week □Three or more a week

b) Wake up in the middle of the night or early morning

□None □Less than once a week

□Once or twice a week □Three or more a week

c) Have to get up to use the bathroom

□None □Less than once a week

□Once or twice a week □Three or more a week

d) Cannot breathe comfortably

□None □Less than once a week

□Once or twice a week □Three or more a week

e) Cough or snore loudly

□None □Less than once a week

□Once or twice a week □Three or more a week

f) Feel too cold

□None □Less than once a week

□Once or twice a week □Three or more a week

g) Feel too hot

□None □Less than once a week

□Once or twice a week □Three or more a week

h) Had bad dreams

□None □Less than once a week

□Once or twice a week □Three or more a week

i) Have pain

□None □Less than once a week

□Once or twice a week □Three or more a week

j) Other reason(s), please describe__________________________

□None □Less than once a week

□Once or twice a week □Three or more a week

6. During the past month, how would you rate your sleep quality overall?

□Very good □Fairly good

□Fairly bad □Very bad

7. During the past month, how often have you taken medicine to help you sleep (prescribed or "over the counter")?

□None □Less than once a week

□Once or twice a week □Three or more a week

8. During the past month, how often have you had trouble staying awake while driving, eating meals, or engaging in social activity?

□None □Less than once a week

□Once or twice a week □Three or more a week

1. During the past month, how much of a problem has it been for you to keep up enough enthusiasm to get things done?

□No problem at all □Only a very slight problem

□Somewhat of a problem □A very big problem

1. Do you have a bed partner or room mate?

□No bed partner or room mate □Partner/room mate in other room

□Partner in same room, but not same bed □Partner in same bed

If you have a room mate or bed partner, ask him/her how often in the past month you have had . . .

a) Loud snoring

□None □Less than once a week

□Once or twice a week □Three or more a week

b) Long pauses between breaths while asleep

□None □Less than once a week

□Once or twice a week □Three or more a week

c) Legs twitching or jerking while you sleep

□None □Less than once a week

□Once or twice a week □Three or more a week

d) Episodes of disorientation or confusion during sleep

□None □Less than once a week

□Once or twice a week □Three or more a week

e) Other restlessness while you sleep: please describe ___________________

□None □Less than once a week

□Once or twice a week □Three or more a week

**APPENDIX G. Pressure pain threshold (PPT)**

| Location | the first time | | the second time | | the third time | |
| --- | --- | --- | --- | --- | --- | --- |
|  | Left | Right | Left | Right | Left | Right |
| Masseter PPT, 0-500 kPa |  |  |  |  |  |  |
| Anterior temporalis PPT, 0-500 kPa |  |  |  |  |  |  |
| Sternocleidomastoid PPT, 0-500 kPa |  |  |  |  |  |  |
| Trapezius PPT, 0-500 kPa |  |  |  |  |  |  |
| TMJ PPT, 0-500 kPa |  |  |  |  |  |  |

TMJ=temporomandibular joint

**APPENDIX H. Surface Electromyography (sEMG)**

| Location | the first time (μV) | the second time (μV) | the third time (μV) |
| --- | --- | --- | --- |
| **Mandibular resting position (MR)** |  |  |  |
| RMS, Masseter muscle, left |  |  |  |
| RMS, Masseter muscle, right |  |  |  |
| RMS, Anterior temporalis muscle, left |  |  |  |
| RMS, Anterior temporalis muscle, right |  |  |  |
| **Habitual chewing (HC)** |  |  |  |
| RMS, Masseter muscle, left |  |  |  |
| RMS, Masseter muscle, right |  |  |  |
| RMS, Anterior temporalis muscle, left |  |  |  |
| RMS, Anterior temporalis muscle, right |  |  |  |
| **Maximal voluntary contraction (MVC)** |  |  |  |
| RMS, Masseter muscle, left |  |  |  |
| RMS, Masseter muscle, right |  |  |  |
| RMS, Anterior temporalis muscle, left |  |  |  |
| RMS, Anterior temporalis muscle, right |  |  |  |

RMS=Root Mean Square; MR=Mandibular resting position; HC=Habitual chewing; MVC=Maximal voluntary contraction.

**APPENDIX I. Acupuncture Expectancy Scale**

Every individual may have different expectation for the effects of acupuncture. If we use the following sentence to describe your expectation of acupuncture’s effect on your illness/symptom after the entire course of acupuncture therapy, how much do you agree? please choose the closest answer.

| **Acupuncture Expectancy Scale** | |
| --- | --- |
| My illness will improve a lot? | □Ineffective  □May be ineffective  □Unclear  □May be effective  □Effective |

**APPENDIX J. Participant’s compliance**

|  | Week 1 | Week 2 | Week 3 | Week 4 | Total sessions |
| --- | --- | --- | --- | --- | --- |
| The number of treatment sessions should received | 3 | 3 | 3 | 3 | 12 |
| The actual number of treatment sessions received |  |  |  |  |  |
| The missed number of treatment sessions received |  |  |  |  |  |

**APPENDIX K. Participants’ satisfaction scale**

| **Participants’ satisfaction scale survey** | |
| --- | --- |
| Are you satisfied with your current treatment？ | □Extremely satisfied  □Satisfied  □Moderately satisfied  □Dissatisfied  □Extremely dissatisfied |

**APPENDIX L. Blinding assessment**

| At week 4 | | | |
| --- | --- | --- | --- |
| What kind of treatment do you think you received | □Think in Acupuncture group | □Think in Sham acupuncture group | □Did not know |


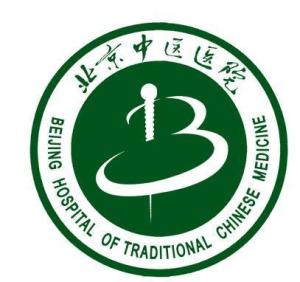


**Effect of acupuncture for temporomandibular disorders: a randomized clinical trial**

**STATISTICAL ANALYSIS PLAN**

**Beijing Hospital of Traditional Chinese Medicine, Capital Medical University, Beijing, China**

**Version: 1.1**

**9 May 2022**

**TABLE OF CONTENTS**

**[LIST OF ABBREVIATIONS 3](#_Toc14714)**

**[1. INTRODUCTION 4](#_Toc23131)**

**[2. STUDY OBJECTIVES AND OUTCOMES 4](#_Toc27724)**

**[3. STUDY DESIGN 6](#_Toc16430)**

[3.1 General Design 6](#_Toc12566)

[3.2 Sample Size and Power Considerations 8](#_Toc15274)

[3.3 Randomization and Blinding 8](#_Toc23353)

**[4. ANALYSIS SETS](#_Toc31000)** [9](#_Toc31000)

[4.1 Intention-to-treat Analysis Set 9](#_Toc14981)

[4.2 Per-protocol Analysis Set 9](#_Toc13071)

[4.3 Safety Analysis Set 9](#_Toc10545)

**[5. GENERAL ISSUES FOR DATA ANALYSIS](#_Toc9466)** [9](#_Toc9466)

[5.1 General 9](#_Toc24259)

[5.2 Specification of Baseline Value 9](#_Toc4841)

[5.3 Multiple Comparisons and Multiplicity 10](#_Toc22311)

**[6. STUDY POPULATION SUMMARY](#_Toc6345)** [10](#_Toc6345)

[6.1 Participant Disposition 10](#_Toc22401)

[6.2 Withdrawals 10](#_Toc17524)

[6.3 Demographics and Baseline Characteristics 11](#_Toc9849)

[6.4 Diary Compliance 11](#_Toc22848)

**[7. PROTOCOL DEVIATIONS 11](#_Toc31771)**

**[8. EFFICACY ANALYSIS 11](#_Toc20717)**

[8.1 General 11](#_Toc3453)

[8.2 Primary Efficacy Variable and Analysis 12](#_Toc3366)

[8.2.1 Primary Efficacy Analysis 12](#_Toc31434)

[8.3 Secondary Efficacy Variables and Analysis 12](#_Toc1526)

[8.3.1 Variable Definition 12](#_Toc23133)

[8.3.2 Secondary Efficacy Analysis 21](#_Toc8101)

[8.4 Exploratory Efficacy Variables and Analysis 21](#_Toc24873)

[8.4.1 Variable Definition 21](#_Toc4211)

[8.4.2. Questionnaires During Follow-up Period 21](#_Toc31875)

[8.4.3. Exploratory Efficacy Analysis 21](#_Toc2622)

**[9. SAFETY ANALYSIS](#_Toc27706)** [22](#_Toc27706)

[9.1 General 22](#_Toc25068)

[9.2 Adverse Events 22](#_Toc13906)

[9.3 Clinical Laboratory Tests/ Vital Signs/ Weight 22](#_Toc16226)

**[10.REFERENCES](#_Toc23897)** [23](#_Toc23897)

**[APPENDIX](#_Toc13353)** [24](#_Toc13353)

[APPENDIX A. Graded Chronic Pain Scale Version 2.0 (GCPS 2.0) 24](#_Toc18703)

[APPENDIX B. Jaw Functional Limitation Scale-20 (JFLS-20) 26](#_Toc31760)

[APPENDIX C. Depression Anxiety Stress Scale-21 (DASS-21) 27](#_Toc5342)

[APPENDIX D. Pittsburgh Sleep Quality Index (PSQI) 29](#_Toc24627)

**LIST OF ABBREVIATIONS**

| **Abbreviation** | **Definition** |
| --- | --- |
| AE | Adverse Event |
| CIs | Confidence Intervals |
| CPI | Characteristic Pain Intensity |
| DASS-21 | Depression Anxiety and Stress Scale-21 |
| DC/TMD | Diagnostic Criteria for Temporomandibular disorders |
| GCPS | Graded Chronic Pain Scale |
| HC | Habitual Chewing |
| ITT | Intention-to-Treat |
| JFLS-20 | Jaw Function Limitation Scale-20 |
| LSM | Least-Squares Mean |
| MVC | Maximum Voluntary Contraction |
| PP | Per-Protocol |
| PPT | Pressure Pain Threshold |
| PSQI | Pittsburgh Sleep Quality Index |
| RMS | Root Mean Square |
| SAP | Statistical Analysis Plan |
| SAE | Serious Adverse Event |
| SE | Standard Error |
| sEMG | Surface Electromyogram |
| SD | Standard Deviation |
| TMD | Temporomandibular Disorders |
| VAS | Visual Analogue Scale |

# INTRODUCTION

This Statistical Analysis Plan (SAP) describes the planned analysis and reporting based on the protocol for the clinical trial entitled: Effect of acupuncture for temporomandibular disorders: a randomized clinical trial. Study protocol version 1.1 was reviewed in preparation of this SAP.

The purpose of this SAP is to outline the planned analyses to be completed to support the completion of the trial reports. The planned analyses identified in this SAP will be included in future manuscripts.

# STUDY OBJECTIVES AND OUTCOMES

| Objectives | Outcomes |
| --- | --- |
| Primary Objectives  •To evaluate the efficacy of acupuncture for the temporomandibular disorder participants | •Primary Outcomes  - Change from baseline in mean weekly pain intensity at week 4  •Secondary Outcomes  - ≥30% reduction in mean weekly pain intensity at week 4  - ≥50% reduction in mean weekly pain intensity at week 4 |
| Secondary Objectives  •To evaluate the physical functioning  •To evaluate the emotional functioning  •To evaluate the additional treatment outcomes | •Secondary Outcomes  - Mean change from baseline in jaw opening and movement at week 4  - Mean change from baseline in Graded Chronic Pain Scale (GCPS) at week 4  - Mean change from baseline in Jaw Functional Limitations Scale-20 (JFLS-20) at week 4  - Mean change from baseline in Depression, Anxiety and Stress Scales-21 (DASS-21) at week 4  - Mean change from baseline in Pittsburgh sleep quality index (PSQI) at week 4  - Mean change from baseline in Pressure Pain Threshold (PPT) at week 4  - Mean change from baseline in surface electromyogram (sEMG) at week 4 |
| Exploratory Objectives  •To evaluate the long-term efficacy of acupuncture | Exploratory Outcomes  - Mean change from baseline in weekly pain intensity at week 8  - ≥30% reduction in pain intensity at week 8  - ≥50% reduction in pain intensity at week 8  - Mean change from baseline in jaw opening and movement at week 8  - Mean change from baseline in Graded Chronic Pain Scale (GCPS) at week 8  - Mean change from baseline in Jaw Functional Limitations Scale-20 (JFLS-20) at week 8  - Mean change from baseline in Depression, Anxiety and Stress Scales-21 (DASS-21) at week 8  - Mean change from baseline in Pittsburgh sleep quality index (PSQI) at week 8 |
| Safety Objectives  •To evaluate the safety of acupuncture | •Safety Outcomes  - Occurrence of adverse events throughout the study  - Abnormal vital signs (systolic and diastolic blood pressure, pulse, and respiratory rate)  - Weight  - Potentially clinically significant abnormalities in clinical laboratory tests result (blood routine test, blood biochemistry test, blood coagulation test, urinalysis test and urine pregnancy test) |

# STUDY DESIGN

## 3.1 General Design

This study is a single-blind randomized controlled trial in China to evaluate the efficacy and safety of acupuncture for the temporomandibular disorders (TMD) participants. It will comprise a 1-week screening period, a 1-week baseline period, a 4-week treatment period, and a 4-week follow-up period. This study will include female and male participants, aged 18 to 80 years, suffering from TMD for at least three months before screening, meeting the Diagnostic Criteria for pain-related TMD in accordance with DC/TMD Temporomandibular Disorders (DC/TMD).^2^

It is planned that 60 patients will be randomly allocated to one of two treatment groups: acupuncture group or sham acupuncture group, in a ratio of 1:1. The patient will receive treatment for 4 weeks. Patients assigned to acupuncture group will receive a 12-session acupuncture treatment (3 sessions a week, ideally every other day). Patients assigned to the sham acupuncture group will receive sham acupuncture intervention with Park sham device. An overview of the study is presented in **Figure 1** and the scheduled study procedures and assessments are summarized in **Table 1**.


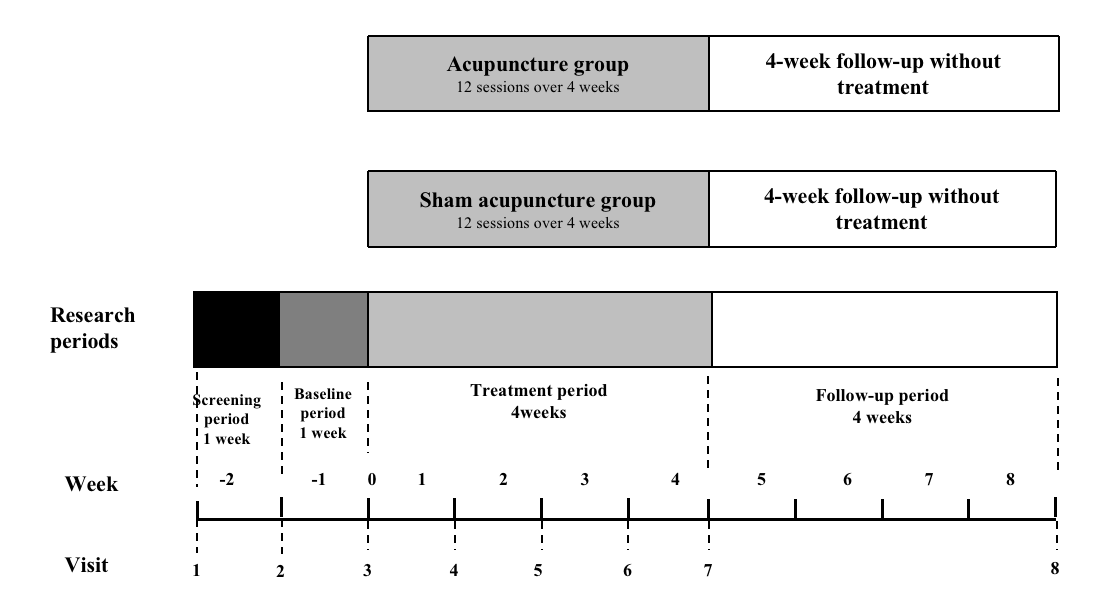


**Figure 1. Study design.** The study for each participant will be divided into 4 periods: a 1-week screening period, a 1-week baseline period, a 4-week treatment period, and a 4-week follow-up period.

**Table 1. The schedule of enrolment, interventions, and assessments**

| **STUDY PERIOD** | | | | | | | | |
| --- | --- | --- | --- | --- | --- | --- | --- | --- |
|  | **Screening** | **Baseline** | **Allocation** | **Treatment** | | | | **Follow-up** |
| **TIMEPOINT**  **(W, week)** | -2 | -1 | 0 | 1 | 2 | 3 | 4 | 8 |
| **Enrolment** | | | | | | | | |
| Informed consent | **×** |  |  |  |  |  |  |  |
| Eligibility criteria | **×** | **×** |  |  |  |  |  |  |
| Demography Characteristics |  | **×** |  |  |  |  |  |  |
| Disease history of TMD |  | **×** |  |  |  |  |  |  |
| Randomization |  |  | **×** |  |  |  |  |  |
| **Interventions** | | | | | | | | |
| Acupuncture group |  |  |  |  | | | |  |
| Sham acupuncture group |  |  |  |  | | | |  |
| **Assessments** | | | | | | | | |
| TMD diary |  |  | | | | | | |
| VAS |  |  | **×** |  |  |  | **×** | **×** |
| Jaw opening and movement |  |  | **×** |  |  |  | **×** | **×** |
| GCPS |  |  | **×** |  |  |  | **×** | **×** |
| JFLS-20 |  |  | **×** |  |  |  | **×** | **×** |
| DASS-21 |  |  | **×** |  |  |  | **×** | **×** |
| PSQI |  |  | **×** |  |  |  | **×** | **×** |
| PPT |  |  | **×** |  |  |  | **×** |  |
| sEMG |  |  | **×** |  |  |  | **×** |  |
| Laboratory test* |  |  | **×** |  |  |  | **×** |  |
| Acupuncture expectancy scale |  |  | **×** |  |  |  |  |  |
| Participant’s satisfaction scale |  |  |  |  |  |  | **×** |  |
| Participant’s compliance |  |  |  | **×** | **×** | **×** | **×** |  |
| Blinding assessment |  |  | **×** |  |  |  | **×** |  |
| Clinical laboratory tests |  |  | **×** |  |  |  | **×** |  |
| Vital signs |  |  | **×** |  |  |  | **×** | **×** |
| Weight |  |  | **×** |  |  |  | **×** | **×** |
| Adverse events |  |  |  | | | | | |

VAS=visual analogue scale；GCPS=Graded Chronic Pain Scale；JFLS-20=Jaw Function Limitation Scale-20；DASS-21=Depression Anxiety and Stress Scale-21；PSQI=Pittsburgh Sleep Quality Index.

*The laboratory test includes blood and urine samples for the clinical safety.

## 3.2 Sample Size and Power Considerations

Based on our pilot study, the reduced pain intensity (VAS score) of TMD patients after 4-week treatment was 3.4±0.3 in the acupuncture group and 2.9±0.7 in the sham acupuncture group. A sample size of 50 patients gives at least 90% power for the study to succeed at an alpha level of 0.05. Assuming a 10% loss rate of follow-up, 30 patients per treatment group were planned for randomization.

## 3.3 Randomization and Blinding

Eligible participants will be randomly assigned (1:1) to either acupuncture group or sham acupuncture group using an interactive web-based response system (Beijing LNKMED Tech Co., Ltd, Beijing, China) The randomization sequence will be computer generated by an independent statistician who do not participate in the trial. After a participant’s eligibility is determined, a dedicated investigator will access the randomization interface and completed randomization form (each participant’s name, sex, and birthday), and then the random number and group assignment will be displayed on the web interface. Afterwards, this dedicated investigator will inform acupuncturists the randomization.

The participants, outcome assessors, and the statistician will be blinded to treatment assignment. Unmasked personnel will include the dedicated investigator responsible for the randomization module. Because of the nature of the intervention, acupuncturists cannot be blinded, but they will be trained not to communicate with participants or outcome assessors about treatment procedures and responses.

# 4. ANALYSIS SETS

## 4.1 Intention-to-treat Analysis Set

The Intention-to-Treat (ITT) analysis set will include all randomized participants. In this population, treatment will be assigned based on the treatment to which participants are randomized, regardless of which treatment they actually received.

## 4.2 Per-protocol Analysis Set

The per-protocol (PP) analysis set was a subset of the ITT analysis set, including only participants who completed the study without any violations of the inclusion/exclusion criteria, or any deviations or omissions in the study treatment plan.

## 4.3 Safety Analysis Set

The safety analysis set will include all participants who receive at least one treatment. In this population, treatment will be assigned based upon the treatment participants actually receive, regardless of the treatment to which they are randomized.

# 5. GENERAL ISSUES FOR DATA ANALYSIS

## 5.1 General

All summary statistics will be computed and displayed by treatment group. In general, summary statistics (count [n], mean, least-squares mean [LSM], standard deviation [SD], standard error [SE], median, lower and upper quartiles, minimum and maximum values) will be presented for continuous variables and counts and, if relevant, percentages will be presented for categorical variables. Graphical data displays may also be used as appropriate.

## 5.2 Specification of Baseline Value

The baseline efficacy variables that will be summarized will be split into baseline TMD characteristics and other efficacy variables.

The efficacy baseline values at baseline derived from baseline measurement include:

1. disease duration, years

2. pain intensity (VAS) at baseline

The other baseline efficacy variables that will be summarized include：

1. physical functioning

- measurement of pain-free jaw opening at baseline

- measurement of maximum unassisted jaw opening at baseline

- measurement of maximum assisted jaw opening at baseline

- measurement of protrusion movement at baseline

- measurement of lateral movement at baseline (left/right)

- GCPS total score at baseline

- JFLS-20 total score at baseline

2. emotional functioning

- DASS-21score at baseline

- PSQI score at baseline

3. additional treatment outcomes

- Pressure Pain Threshold (PPT) at baseline

- Surface electromyogram (sEMG) at baseline

## 5.3 Multiple Comparisons and Multiplicity

Since only one primary outcome is defined, no adjustments to the significance level will be required to account for multiple testing.

For the analysis of the secondary and safety outcomes, no adjustment for multiple comparisons will be made.

# 6. STUDY POPULATION SUMMARY

## 6.1 Participant Disposition

Data from participants screened, participants screened but not randomized and reason not randomized, participants who are randomized (i.e., in the ITT set), participants randomized but not treated, participants in the safety and other analysis sets, participants who complete the study, and participants who withdraw from the study will be summarized using descriptive statistics. Data from participants who withdraw from the study will also be summarized by reason for withdrawal using descriptive statistics.

## 6.2 Withdrawals

For each period, the number of participants who withdraw from treatment, which also implies that they withdraw from the study, will be summarized by group, and primary reason for withdrawal, and by treatment group and all reasons for withdrawal.

Participants who withdraw will be listed for each period. The listings will include the number of days in the study until withdrawal, the date of first treatment, the date of the latest treatment prior to withdrawal, the number of days since latest treatment, the primary reason for withdrawal, all reasons for withdrawal.

## 6.3 Demographics and Baseline Characteristics

Participant’s demographics and baseline characteristics, including age, gender, body-mass index, current employment status, education, marital status, annual income, and facial pain will be summarized for the ITT population. The baseline diary efficacy variables listed in **section 8.1**, jaw opening and movement measurement, GCPS total score, JFLS-20 total score, DASS-21 total score, PSQI total score, PPT, sEMG, and expectation assessment, will be summarized by treatment group for the ITT set.

## 6.4 Diary Compliance

At least 85% compliance of TMD diary is needed during the baseline period and 50% during the treatment and follow-up periods. And the compliance will be assessed every four weeks.

# 7. PROTOCOL DEVIATIONS

Protocol violations include enrolling participants in violation of key eligibility criteria designed to ensure a specific subject population, failing to collect data necessary to interpret outcomes, noncompliance to study treatment administration, or any other deviations that may have an impact on the processes put in place for the care and safety of the participants or compromise the scientific value of the trial.

# 8. EFFICACY ANALYSIS

**8.1 General**

The efficacy data for this study consist of TMD pain related responses (e.g., facial pain intensity) collected after a 4-week treatment or follow-up.

In addition, the following questionnaires will be used for the assessments of efficacy, physical functioning, emotional functioning and additional treatment outcomes during the study.

- Pain related index measured by pain intensity (VAS score) and ≥30%, ≥50% reduction in pain intensity.

- Physical functioning measured by GCPS (**Appendix A**) and JFLS-20 (**Appendix B**).

- Emotional functioning measured by DASS-21 (**Appendix C**) and PSQI (**Appendix D**).

- Additional treatment outcomes measured by PPT and sEMG

The primary analysis will be performed on the ITT set, and the sensitivity analysis will be performed on the PP set.

## 8.2 Primary Efficacy Variable and Analysis

For the purpose of this study, the change of pain intensity will be defined as the reduced score of weekly pain intensity (reported on a 0-10 VAS) from baseline to the last week of treatment period.

The primary statistical analysis to evaluate the primary hypothesis will use all data from the ITT analytical sample in a linear regression model. Mixed models will be used to analyze the treatment effect at Week 4.

The weekly pain intensity was calculated if a participant completed at least 4 of the 7 daily reports of pain intensity. For patients with missing days and fewer than 4 days of TMD diary data for 1 week, the weekly pain intensity was considered missing before the multiple imputation procedure. The statistics were based on 10 sets of imputed data, where the mean is the average of the means from the 10 data sets and the standard error (SE) of the mean is adjusted based on the imputation variance estimates.

### 8.2.1 Primary Efficacy Analysis

The hypothesis testing for the primary analysis is:

*H_0_: δ_1_=δ_2_*

*H_1_: δ_1_≠δ_2_*

where δ1 and δ2 are the estimates of weekly pain intensity change from baseline to the last week of treatment for the acupuncture group and the sham acupuncture group respectively.

The primary efficacy endpoint was the change from baseline in mean weekly pain intensity at week 4, the main analysis was performed using an analysis of linear regression model adjusted for sex, age, weekly pain intensity at baseline, TMD-specific medication. The LSM change from baseline with SE was presented for each treatment group, and the LSM with 95% confidence intervals (95% CIs) for the treatment differences, and associated p-values will be provided. A corresponding sensitivity analysis will be performed on the PP set using a linear regression model as specified for the primary efficacy outcome on the ITT set.

## 8.3 Secondary Efficacy Variables and Analysis

### 8.3.1 Variable Definition

#### 8.3.1.1 TMD Related Data During Treatment Period

##### 8.3.1.1.1 Responder Rates

The following responder rates will be derived: 30% and 50%. A responder is a participant, who achieves a ≥30% reduction or ≥50% reduction in pain intensity at week 4, compared to baseline pain intensity. The population-level summary is the odds ratio of a successful response between two groups.

##### 8.3.1.1.2 Jaw Opening and Movement

In addition to TMD classification, these examination findings are presented as secondary outcomes: pain-free opening, maximum unassisted jaw opening, maximum assisted jaw opening, forward extension and lateral movement (left/right).

Pain-free opening and maximum unassisted opening are measured by the interincisal distance between the maxillary and mandibular reference teeth. In certain settings, repeating the test, if the pain-free opening is less than 30 mm, might be appropriate in order to assess for possible low-range outlying values. Ask the patient about any pain produced by measuring maximum unassisted jaw opening.

Maximum assisted jaw opening is examiner-based. The examiner uses moderate pressure, push the mouth open further, measures the interincisal distance between the maxillary and mandibular teeth.

Lateral excursive measurements are made between the maxillary and mandibular reference midlines, while protrusive excursive measurement is made between the labial surfaces of the maxillary and mandibular reference teeth. For the lateral excursive movements, if the patient is confused about direction s/he should move his or her jaw, touch the ipsilateral side of the face, lip, or even shoulder, and ask the patient to move towards the indicated side. Note that if the mandibular incisors cannot be protruded beyond the maxillary incisors, the value will be negative. If anterior cross-bite situation exists, the field “if negative” on the examination form will be marked. The protrusive movement measured as the distance from the labial surfaces of the maxillary to mandibular incisors, will be recorded as a positive number.

#### 8.3.1.2. Questionnaires During Treatment Period

##### 8.3.1.2.1 Graded Chronic Pain Scale

This scale captures disability, pain intensity (high and low impact), and whether an individual is able to work due to pain.^26^ GCPS includes, in addition to the 3 items for pain intensity and 4 items for function, one item for number of days of pain. Each item has a score of 0-10. Characteristic Pain Intensity (CPI): compute mean of items 2-4 (pain right now, worst pain, average pain), and multiply by 10. Interference Score: compute mean of items 6-8 (daily activities, social activities, work activities), and multiply by 10. Disability score for number of days with interference: assign points based on below table. Disability score for the interference score: assign points based on **Table 2**.

**Table 2. Rating scale of disability points**

| **Points for Disability Days** | | **Points for Pain-related** | |
| --- | --- | --- | --- |
| 1 month（30 days） | | Interference Score | |
| Days | Points | Interference | Points |
| 0-1 | 0 | 0-29 | 0 |
| 2 | 1 | 30-49 | 1 |
| 3-5 | 2 | 50-69 | 2 |
| 6+ | 3 | 70+ | 3 |

The total Disability Score=Points for Disability Days + Points for Interference Score.

Determination of graded chronic pain status based on CPI and Disability Points. The Specific details based on **Table 3**.

If one or more responses are missing among items 2-4 (pain intensity), the respective subscale should not be scored due to the broad scope that the three items cover. For the function items (6-8), one missing value may not represent the same information loss, and the subscale score could be computed albeit with decreased reliability. Missing data for number of disability days precludes determination of graded chronic pain status.

**Table 3. Determination of chronic pain grade**

| **Grade** | **Label** | **CPI** | **Disability Points** |
| --- | --- | --- | --- |
| 0 | None | 0 | N/A |
| 1 | Low intensity pain, with none-low pain-related disability | <50 | <3 |
| 2 | High intensity pain, with none-low pain-related disability | ≥50 | <3 |
|  | [2a]High intensity pain, without pain-related disability | ≥50 | <3 |
|  | [2b]High intensity pain, with low pain-related disability | ≥50 | <3 |
| 3 | Moderately limiting | N/A | 3-4 |
| 4 | Severely limiting | N/A | 5-6 |

##### 8.3.1.2.2. JFLS-20

The JFLS-20 is an organ-specific instrument comprising 3 constructs for assessing functional status of the masticatory system; the 3 scales exhibit properties that are ideal for both research and patient evaluation in patient groups with a range of functional limitations of the jaw.^27^ The 20-item JFLS covers activities involving social aspects such as facial expressions (happy and angry), kissing, singing, frowning, laughing, and other jaw activities such as chewing a hard bread, chewing crackers, eating soft food that requires no chewing, opening the mouth wide to bite an apple or a sandwich, and talking. A response scale used 0 to 10 signifying degree of limitation (0=no limitation and 10=severe limitation); an option for “not applicable” is also available, and is scored as “missing.” The subscales are computed as the mean response for all items in the subscale. Mastication is based on 6 items, vertical jaw mobility is based on 4 items, and verbal and emotional expression is based on 8 items; 2 items are not scored as part of these 3 subscales. A total score is also computed from the 3 subscales when all 3 component scores are available. The JFLS-20 is designed based on item response theory, and consequently identical non-zero responses to all items within a subscale are highly improbable; non-zero responses that are the same within a subscale were regarded as invalid and recoded to “missing.”

Scoring:

From either the short form (all items) or the long form (items 1, 3, 6, 10, 11, 12, 13, and 19), a single global score of “jaw functional limitation” can be computed as the mean of the available items. Subscale scores for each type of functional limitation are computed, as follows:

Mastication: mean of items 1-6.

Vertical jaw mobility: mean of items 7-10.

Verbal and emotional expression: mean of items 13-20.

A second type of global score can be obtained from the long form by computing the mean of the 3 subscale scores, as computed above. Note that all 3 subscale scores must be present in order to compute the global score in this manner.

For the JFLS-20, scores can be computed based on no more than the following number of items with missing response: short form, 2 items missing allowed; mastication, 2 items missing allowed; mobility, 1 item missing allowed; and communication, 2 items missing allowed. Norms have not yet been established for this instrument.

##### 8.3.1.2.3. Depression Anxiety Stress Scales 21 (DASS-21)

The DASS is a set of three self-report scales designed to measure the negative emotional states of depression, anxiety and stress. The DASS was constructed not merely as another set of scales to measure conventionally defined emotional states, but to further the process of defining, understanding, and measuring the ubiquitous and clinically significant emotional states usually described as depression, anxiety and stress. Each of the three DASS scales contains 7 items, divided into subscales with similar content. The Depression scale (item 3,5,10,13,16,17,21) assesses dysphoria, hopelessness, devaluation of life, self-deprecation, lack of interest/involvement, anhedonia, and inertia. The Anxiety scale (item 2,4,7,9,15,19,20) assesses autonomic arousal, skeletal muscle effects, situational anxiety, and subjective experience of anxious affect. The Stress scale (item 1,6,8,11,12,14,18) is sensitive to levels of chronic non-specific arousal. It assesses difficulty relaxing, nervous arousal, and being easily upset/agitated, irritable/over-reactive and impatient. Subjects are asked to use 4-point severity/frequency scales to rate the extent to which they have experienced each state over the past week. Scores for Depression, Anxiety and Stress are calculated by summing the scores for the relevant items. To calculate comparable scores with full DASS, each seven-item scale was multiplied by two. Participants were asked to rate how many of each of the items (in the form of statements) applied to them over the past week, with 0=did not apply to me at all to 3=applied to me very much, or most of the time. The higher the score, the more severe the emotional distress was.

##### 8.3.1.2.4. Pittsburgh Sleep Quality Index (PSQI)

PSQI aims to assess a person’s sleep quality with questions relating to their sleeping habits within the past month. The PSQI consists of 18 self-rated questions and five questions raised by the bedpartner or roommate **(Table 4)**. The latter five questions are used for clinical information only, are not tabulated in the scoring of the PSQI. These 19 items are grouped into seven component scores, each weighted equally on a 0-3 scale. Higher scores indicate worse sleep quality. These components are subjective sleep quality, sleep latency, sleep duration, habitual sleep efficiency, sleep disturbances, use of sleeping medications, and daytime dysfunction over the past month. The seven-component scores are added in order to obtain a “global” score ranging from 0 to 21 points, where “0” indicates no difficulty and “21” indicates major difficulties in all areas. The global PSQI score, ranging from 0 to 21, can be collected by summing the seven components after weighting them on a scale ranging from 0 to 3. For each component, as well as the global PSQI score, higher sleep scores show worse sleep quality. The global score < 5 associated with good sleep quality while > 5 associated with poor sleep quality.

**Table 4. Scoring rule of pittsburgh sleep quality index**

| **component1**  **PSQISLPQUAL** | **OVERALL SLEEP QUALITY** | **Q6**  **Minimum Score = 0 (better); Maximum Score = 3 (worse)** |
| --- | --- | --- |
| component2 PSQILATEN | SLEEP LATENCY | **First, recode Q2 into Q2new thusly:**  IF Q2 > 0 and < 15, THEN set value of Q2new to 0  IF Q2 > 15 and < 30, THEN set value of Q2new to 1  IF Q2 > 30 and < 60, THEN set value of Q2new to 2  IF Q2 > 60, THEN set value of Q2new to 3  **Next**  IF Q5a + Q2new = 0, THEN set value to 0  IF Q5a + Q2new > 1 and < 2, THEN set value to 1  IF Q5a + Q2new > 3 and < 4, THEN set value to 2  IF Q5a + Q2new > 5 and < 6, THEN set value to 3  Minimum Score = 0 (better);  Maximum Score = 3 (worse) |
| component3  PSQIDURAT | DURATION OF SLEEP | IF Q4 > 7, THEN set value to 0  IF Q4 < 7 and > 6, THEN set value to 1  IF Q4 < 6 and > 5, THEN set value to 2  IF Q4 < 5, THEN set value to 3  Minimum Score = 0 (better);  Maximum Score = 3 (worse) |
| component4  PSQIHSE | SLEEP EFFICIENCY | Diffsec = Difference in seconds between day and time of day Q1 and day Q3  Diffhour = Absolute value of diffsec / 3600  newtib =IF diffhour > 24, then newtib = diffhour – 24  IF diffhour < 24, THEN newtib = diffhour  (NOTE, THE ABOVE JUST CALCULATES THE HOURS BETWEEN GNT (Q1) AND GMT (Q3))  tmphse = (Q4 / newtib) * 100  IF tmphse > 85, THEN set value to 0  IF tmphse < 85 and > 75, THEN set value to 1  IF tmphse < 75 and > 65, THEN set value to 2  IF tmphse < 65, THEN set value to 3  Minimum Score = 0 (better); Maximum Score = 3 (worse) |
| component5  PSQIDISTB | SLEEP DISTURBANCE | IF Q5b + Q5c + Q5d + Q5e + Q5f + Q5g + Q5h + Q5i + Q5j (IF Q5JCOM is null or Q5j is null, set the value of Q5j to 0) = 0, THEN set value to 0  IF Q5b + Q5c + Q5d + Q5e + Q5f + Q5g + Q5h + Q5i + Q5j (IF Q5JCOM is null or Q5j is null, set the value of Q5j to 0) > 1 and < 9, THEN set value to 1  IF Q5b + Q5c + Q5d + Q5e + Q5f + Q5g + Q5h + Q5i + Q5j (IF Q5JCOM is null or Q5j is null, set the value of Q5j to 0) > 9 and < 18, THEN set value to 2  IF Q5b + Q5c + Q5d + Q5e + Q5f + Q5g + Q5h + Q5i + Q5j (IF Q5JCOM is null or Q5j is null, set the value of Q5j to 0) > 18, THEN set value to 3  Minimum Score = 0 (better);  Maximum Score = 3 (worse) |
| component6  PSQIMEDS | NEED MEDS TO SLEEP | Q7  Minimum Score = 0 (better);  Maximum Score = 3 (worse) |
| component7  PSQIDAYDYS | DAY DYSFUNCTION DUE TO SLEEPINESS | IF Q8 + Q9 = 0, THEN set value to 0  IF Q8 + Q9 > 1 and < 2, THEN set value to 1  IF Q8 + Q9 > 3 and < 4, THEN set value to 2  IF Q8 + Q9 > 5 and < 6, THEN set value to 3  Minimum Score = 0 (better);  Maximum Score = 3 (worse) |
| PSQI | TOTAL | DURAT + DISTB + LATEN + DAYDYS + HSE + SLPQUAL + MEDS  Minimum Score = 0 (better);  Maximum Score = 21 (worse)  Interpretation:  TOTAL < 5 associated with good sleep quality  TOTAL > 5 associated with poor sleep quality |

Note: PSQISLPQUAL=PSQI sleep quality; PSQILATEN=PSQI sleep latency; PSQIDURAT=PSQI duration of sleep; PSQIHSE=PSQI sleep efficiency; Diffsec=difference in seconds; Diffhour=Difference in hours; GNT=gone to bed at night time; GMT=get up in the morning time; tmphse= time of sleep efficiency; PSQIDISTB=PSQI sleep disturbance; PSQIMEDS=PSQI need medicine to sleep; PSQIDAYDYS=PSQI day dysfunction due to sleepiness; DURAT=duration; DISTB=disturbance; LATEN=latency; DAYDYS=day dysfunction; HSE=sleep efficiency; SLPQUAL=sleep quality; MEDS=medicine to sleep.

##### 8.3.1.2.5. (PPT)

Pressure pain threshold (PPT) is the only test for deep-pain sensitivity, most probably mediated by muscle C- and A-delta fibers. PPT is defined as the amount of pressure at which the participant first perceived the stimulus to be painful. PPT is one of the commonly used evaluation indicators of TMD pain, which is used for quantitative description.

Trained study staff will perform the PPT measurements. An electronic pressure algometer (Somedic Sales AB, Solna, Stockholm, Sweden) will be used to assess the PPT. This device consists of a grip with a pressure-sensitive strain gauge at the tip and a display unit. The 1 cm^2^ probe tip is covered with a 1 mm thick rubber pad to minimize the risk of irritation of the skin. The algometer is held perpendicular to the skin surface over the muscles and the pressure is increased at a standardized rate of 50 kPa per second. The patient is positioned comfortably in a sitting position, with muscle relaxed. And the patients will be instructed to press a signal button when the sensation of “pressure” changed into “pain”. This was first performed over the soft tissue close to the base of the thumb on the dorsal side of the right hand, in order to accustom the subject to the procedure. The following sites are assessed: bilateral masseter muscles, bilateral anterior temporalis muscle, bilateral sternocleidomastoid, bilateral trapezius muscles and the bilateral temporomandibular joint. The values from the right and left sides will be averaged to obtain a single PPT value per anatomical site, and the average of three trials is considered the final PPT value. There is a two-minute interval between the trials at the same muscle site, and a five-second interval between the measurements of one muscle site and the other.^28^

##### 8.3.1.2.6. (sEMG)

Surface Electromyography (sEMG) is a kind of examination that reflects the state and function of the measured muscle by recording changes in electrical signals during muscle activity. The electrical activity of the anterior part of the temporal and masseter muscles is assessed in accordance with the assumptions of surface electromyography (sEMG).^29^

During the sEMG recording, the environment is kept quiet, with the subject seated in a comfortable chair with the soles of the feet flat on the ground with their arms resting on their legs. The head is positioned upright, with the patient looking toward the horizon. Prior to electrode placement, the subjects’ skin is cleaned with alcohol 75% solution, with the aim of eliminating any facial oils or pollution that could interrupt sEMG signals. Double surface electrodes of silver chloride, disposable, bipolar, with self-adhesive gel are placed on the masseter and anterior temporalis bilaterally, in the longitudinal direction of the muscle fibers. Disposable circular electrodes with an interelectrode distance of 20 mm and a ground electrode placed above the crease of the wrist.

Surface EMG was recorded using a Miotec^®^, model Miotool 400 4-channel system. The system acquired 14-bit EMG signals with electrical isolation of 3000 volts, high EMG signal representation across all channels (2000 samples/second per channel), rejection of 110 dB common mode and low noise level < 2 LSB (Low Significant Bit); EMG signals are acquired using Miograph software with 2000 Hz sampling frequency, 20-500 Hz bandpass filter with interference eliminated by the Notch Filter.

A muscle function test is performed before placing the electrodes served to identify the center of the muscles to be analyzed. The electrodes are placed parallel to the muscle origin and insertion. Before the exam, subjects are asked to perform a maximum voluntary contraction (MVC), a five-second isometric contraction of the masseter and anterior temporal muscles, in order to conduct normalization of the data, interpreted subsequently with the MATLAB. EMG signal acquisition are captured in the following three tasks: the mandibular resting position (MR), the habitual chewing (HC), and the maximum voluntary contraction (MVC). When measuring HC, Trident^®^ gum is used for 20 chewing cycles.^30 31^ Potential amplitude is expressed in microvolts (μV) and expressed by root mean square (RMS).

##### 8.3.1.2.7 ****Acupuncture Expectancy Scale****

The acupuncture expectancy scale consists of an item measuring the expectation of improvement of illness due to acupuncture treatment. Patients will be asked to rate from 1 to 5 on a five-point Likert scale, with 1 indicating “Ineffective”, 2 indicating “May be ineffective”, 3 indicating “Unclear”, 4 indicating “May be effective”, 5 indicating “Effective” with the expected improvement as result of acupuncture.

##### 8.3.1.2.8 ****Patient’s satisfaction****

The Participants’ satisfaction scale is a validated generic tool for assessment of patient satisfaction. Patients will rate how they describe the satisfaction since beginning the treatment in this study on a 5-point scale where 1=extremely dissatisfied; 2=dissatisfied; 3=moderately satisfied; 4=satisfied; 5=extremely satisfied. Participants who achieve score 4 or score 5 are considered as responders.

##### 8.3.1.2.9 Blinding assessment

To test the success of blinding, within 5 minutes after treatment at week 4, participants will be told that there are two kinds of treatment groups: “Acupuncture group” and “Sham acupuncture group”, and they will be randomly assigned to either group at 50% chance respectively. Participants will then be asked to answer the question “Do you think which kind of treatment group you have participated in during the past weeks?”The participants will be able to choose one of the following options as the answer: “Acupuncture group”, “Sham acupuncture group” or “Did not know”.

The Bang blinding indices will be used to assess the success of blinding. The Bang blinding index for each group represents the proportion of participants making a correct treatment guess beyond chance; 0 represents perfect blinding, a positive index indicates a correct guess, and a negative index indicates a guess in the opposite direction.

### 8.3.2 Secondary Efficacy Analysis

For the proportion of responders of ≥30%, ≥50% reduction in pain intensity, a logistic regression model was implemented adjusted for sex, age, weekly pain intensity (VAS) at baseline, TMD-specific medication. Mean percentage with SE, and odds ratios with 95% CIs were presented for the efficacy outcomes by 4 weeks.

The analysis of continuous secondary outcomes were performed similarly to the primary efficacy outcome. A linear regression method, which is similar to the primary analysis setup, will be used for the analysis of secondary efficacy variables change from baseline to week 4: jaw opening and movement, GCPS score, JFLS-20 score, DASS-21 score, PSQI score, PPT, sEMG. The LSM change from baseline with SE was presented for each treatment group, and the LSM with 95% CIs for the treatment differences, and associated p-values will be provided.

A corresponding sensitivity analysis will be performed on the PP set using a linear regression model or logistic regression model as specified on the ITT set.

## 8.4 Exploratory Efficacy Variables and Analysis

### 8.4.1 Variable Definition

Change from baseline in mean weekly pain intensity at week 8, jaw opening and movement, the specific contents of the data are described in **section 8.3.1.1.2**

### 8.4.2. Questionnaires During Follow-up Period

Change from baseline in GCPS score, JFLS-20 score, DASS-21 score, PSQI score at week 8. The specific contents of questionnaire are described in **section 9.3.1.2.4**

### 8.4.3. Exploratory Efficacy Analysis

The analysis of change from baseline in mean weekly pain intensity, jaw opening and movement at week 8 are performed similarly to the primary efficacy outcomes. The change from baseline in GCPS score, JFLS-20 score, DASS-21 score, and PSQI score at week 8 will be analyzed using the linear regression method as described in **section 8.3.2**.

A corresponding sensitivity analysis will be performed on the PP set using a linear regression model or logistic regression model as specified on the ITT set.

# 9. SAFETY ANALYSIS

## 9.1 General

The safety population will be used for all safety analyses. Summaries will be presented by treatment group unless specified otherwise.

## 9.2 Adverse Events

Summaries by treatment group will be presented for AEs (overall and by severity), AEs determined by the investigator to be treatment-related AEs (overall and by severity) or SAEs.

The incidence of AE and severity of the AE will be summarized using descriptive statistics. Each patient will be counted only once by using the AEs with the highest severity within each category. Treatment-related AE summaries will include AEs related to acupuncture and sham acupuncture.

Listings for SAEs, AEs will be presented. All information pertaining to AEs noted during the study will be listed by subject, detailing verbatim given by the investigator, date of onset, date of resolution, severity, and relationship to treatment. The onset of AEs will also be shown relative (in number of days) to the first day of treatment. In addition, AE descriptions, and AE by patient number and treatment group will be presented.

## 9.3 Clinical Laboratory Tests/ Vital Signs/ Weight

Any out-of-range values considered clinically significant by the investigator must be recorded as an AE on an Adverse Event Form. The incidence of potentially clinically significant abnormal results will be summarized using descriptive statistics. Listings of participants who have potentially clinically significant abnormal data will be presented.

# 10.REFERENCES

1. Greene CS. Managing the care of patients with temporomandibular disorders: a new guideline for care. *J Am Dent Assoc* 2010;141(9):1086-8. doi: 10.14219/jada.archive.2010.0337 [published Online First: 2010/09/03]

2. Schiffman E, Ohrbach R, Truelove E, et al. Diagnostic Criteria for Temporomandibular Disorders (DC/TMD) for Clinical and Research Applications: recommendations of the International RDC/TMD Consortium Network* and Orofacial Pain Special Interest Group†. *J Oral Facial Pain Headache* 2014;28(1):6-27. doi: 10.11607/jop.1151 [published Online First: 2014/02/01]

3. Research NIoDaC. Prevalence of TMJD and its signs and symptoms https://www.nidcr.nih.gov/research/data-statistics/facial-pain/prevalence2014 [updated 2018 June.

4. Von Korff M, Ormel J, Keefe FJ, et al. Grading the severity of chronic pain. *Pain* 1992;50(2):133-49. doi: 10.1016/0304-3959(92)90154-4 [published Online First: 1992/08/01]

5. Chen H, Slade G, Lim PF, et al. Relationship between temporomandibular disorders, widespread palpation tenderness, and multiple pain conditions: a case-control study. *J Pain* 2012;13(10):1016-27. doi: 10.1016/j.jpain.2012.07.011 [published Online First: 2012/10/04]

6. De La Torre Canales G, Câmara-Souza MB, Muñoz Lora VRM, et al. Prevalence of psychosocial impairment in temporomandibular disorder patients: A systematic review. *J Oral Rehabil* 2018;45(11):881-89. doi: 10.1111/joor.12685 [published Online First: 2018/07/05]

7. Almoznino G, Benoliel R, Sharav Y, et al. Sleep disorders and chronic craniofacial pain: Characteristics and management possibilities. *Sleep Med Rev* 2017;33:39-50. doi: 10.1016/j.smrv.2016.04.005 [published Online First: 2016/06/21]

8. Durham J, Newton-John TR, Zakrzewska JM. Temporomandibular disorders. *Bmj* 2015;350:h1154. doi: 10.1136/bmj.h1154 [published Online First: 2015/03/15]

9. Wieckiewicz M, Boening K, Wiland P, et al. Reported concepts for the treatment modalities and pain management of temporomandibular disorders. *J Headache Pain* 2015;16:106. doi: 10.1186/s10194-015-0586-5 [published Online First: 2015/12/09]

10. de Souza RF, Lovato da Silva CH, Nasser M, et al. Interventions for the management of temporomandibular joint osteoarthritis. *Cochrane Database Syst Rev* 2012;2012(4):Cd007261. doi: 10.1002/14651858.CD007261.pub2 [published Online First: 2012/04/20]

11. Mujakperuo HR, Watson M, Morrison R, et al. Pharmacological interventions for pain in patients with temporomandibular disorders. *Cochrane Database Syst Rev* 2010(10):Cd004715. doi: 10.1002/14651858.CD004715.pub2 [published Online First: 2010/10/12]

12. De Andrade ED, Rizzatti-Barbosa CM, Pinheiro MLP. Pharmacological guidelines for managing temporomandibular disorders. *Brazilian Journal of Oral Sciences* 2004;3(10):503-05.

13. Lipton JA, Ship JA, Larach-Robinson D. Estimated prevalence and distribution of reported orofacial pain in the United States. *J Am Dent Assoc* 1993;124(10):115-21. doi: 10.14219/jada.archive.1993.0200 [published Online First: 1993/10/01]

14. Scrivani SJ, Keith DA, Kaban LB. Temporomandibular disorders. *N Engl J Med* 2008;359(25):2693-705. doi: 10.1056/NEJMra0802472 [published Online First: 2008/12/19]

15. Organization WH. Acupuncture: review and analysis of reports on controlled clinical trials. 2002.

16. Smith P, Mosscrop D, Davies S, et al. The efficacy of acupuncture in the treatment of temporomandibular joint myofascial pain: a randomised controlled trial. *J Dent* 2007;35(3):259-67. doi: 10.1016/j.jdent.2006.09.004 [published Online First: 2006/11/11]

17. Jung A, Shin BC, Lee MS, et al. Acupuncture for treating temporomandibular joint disorders: a systematic review and meta-analysis of randomized, sham-controlled trials. *J Dent* 2011;39(5):341-50. doi: 10.1016/j.jdent.2011.02.006 [published Online First: 2011/03/01]

18. Fernandes AC, Duarte Moura DM, Da Silva LGD, et al. Acupuncture in Temporomandibular Disorder Myofascial Pain Treatment: A Systematic Review. *J Oral Facial Pain Headache* 2017;31(3):225-32. doi: 10.11607/ofph.1719 [published Online First: 2017/07/25]

19. Simma I, Gleditsch JM, Simma L, et al. Immediate effects of microsystem acupuncture in patients with oromyofacial pain and craniomandibular disorders (CMD): a double-blind, placebo-controlled trial. *Br Dent J* 2009;207(12):E26. doi: 10.1038/sj.bdj.2009.959 [published Online First: 2009/10/31]

20. Itoh K, Asai S, Ohyabu H, et al. Effects of trigger point acupuncture treatment on temporomandibular disorders: a preliminary randomized clinical trial. *J Acupunct Meridian Stud* 2012;5(2):57-62. doi: 10.1016/j.jams.2012.01.013 [published Online First: 2012/04/10]

21. Goddard G, Karibe H, McNeill C, et al. Acupuncture and sham acupuncture reduce muscle pain in myofascial pain patients. *J Orofac Pain* 2002;16(1):71-6. [published Online First: 2002/03/14]

22. Shen YF, Goddard G. The short-term effects of acupuncture on myofascial pain patients after clenching. *Pain Pract* 2007;7(3):256-64. doi: 10.1111/j.1533-2500.2007.00140.x [published Online First: 2007/08/24]

23. Park J, White A, Stevinson C, et al. Validating a new non-penetrating sham acupuncture device: two randomised controlled trials. *Acupunct Med* 2002;20(4):168-74. doi: 10.1136/aim.20.4.168 [published Online First: 2003/01/07]

24. Yang JZ. Compendium of acupuncture and Moxibustion. Beijing: People’s Medical Publishing House 1995.

25. Deng LY, Huang LX. Chinese acupuncture and Moxibustion Zheng Zhi Tong Jian. Qingdao, China: Qingdao Press 2004.

26. Chantaracherd P, John MT, Hodges JS, et al. Temporomandibular joint disorders' impact on pain, function, and disability. *J Dent Res* 2015;94(3 Suppl):79s-86s. doi: 10.1177/0022034514565793 [published Online First: 2015/01/13]

27. Ohrbach R, Larsson P, List T. The jaw functional limitation scale: development, reliability, and validity of 8-item and 20-item versions. *J Orofac Pain* 2008;22(3):219-30. [published Online First: 2008/09/11]

28. Christidis N, Omrani S, Fredriksson L, et al. Repeated tender point injections of granisetron alleviate chronic myofascial pain--a randomized, controlled, double-blinded trial. *J Headache Pain* 2015;16:104. doi: 10.1186/s10194-015-0588-3 [published Online First: 2015/12/05]

29. Berni KC, Dibai-Filho AV, Pires PF, et al. Accuracy of the surface electromyography RMS processing for the diagnosis of myogenous temporomandibular disorder. *J Electromyogr Kinesiol* 2015;25(4):596-602. doi: 10.1016/j.jelekin.2015.05.004 [published Online First: 2015/06/10]

30. Botelho AL, Silva BC, Gentil FH, et al. Immediate effect of the resilient splint evaluated using surface electromyography in patients with TMD. *Cranio* 2010;28(4):266-73. doi: 10.1179/crn.2010.034 [published Online First: 2010/11/03]

31. Ferreira AP, Costa DR, Oliveira AI, et al. Short-term transcutaneous electrical nerve stimulation reduces pain and improves the masticatory muscle activity in temporomandibular disorder patients: a randomized controlled trial. *J Appl Oral Sci* 2017;25(2):112-20. doi: 10.1590/1678-77572016-0173 [published Online First: 2017/04/14]

# APPENDIX

**APPENDIX A. Graded Chronic Pain Scale Version 2.0（GCPS 2.0）**

1. On how many days in the last 6 months have you had facial pain?______Days
2. How would you rate your facial pain **RIGHT NOW**? Use a scale from 0 to 10, where 0 is "no pain"and 10 is "pain as bad as could be".

Pain as bad

No pain as could be

0 1 2 3 4 5 6 7 8 9 10

1. In the LAST 30 DAYS, how would you rate your **WORST** facial pain? Use the same scale, where 0 is "no pain" and 10 is "pain as bad as could be".

Pain as bad

No pain as could be

0 1 2 3 4 5 6 7 8 9 10

1. In the LAST 30 DAYS, **ON AVERAGE**, how would you rate your facial pain? Use the same scale where 0 is "no pain" and 10 is "pain as bad as could be". [That is, *your usual pain* at times you were in pain.]

Pain as bad

No pain as could be

0 1 2 3 4 5 6 7 8 9 10

1. In the LAST 30 DAYS, how many days did your facial pain keep you from doing your USUAL ACTIVITIES like work, school, or housework? (every day = 30 days)

____Days

1. In the LAST 30 DAYS, how much has facial pain interfered with your DAILY ACTIVITIES? Use a 0-10 scale, where 0 is "no interference" and 10 is "unable to carry on any activities".

Unable to carry

No interference on any activities

0 1 2 3 4 5 6 7 8 9 10

1. In the LAST 30 DAYS, how much has facial pain interfered with your **RECREATIONAL, SOCIAL AND FAMILY ACTIVITIES**? Use the same scale, where 0 is "no interference" and 10 is "unable to carry on any activities".

Unable to carry

No interference on any activities

0 1 2 3 4 5 6 7 8 9 10

1. In the LAST 30 DAYS, how much has facial pain interfered with your **ABILITY TO WORK**, including housework? Use the same scale, where 0 is "no interference" and 10 is "unable to carry on any activities".

Unable to carry

No interference on any activities

0 1 2 3 4 5 6 7 8 9 10

**APPENDIX B. Jaw Functional Limitation Scale-20（JFLS-20）**

For each of the items below, please indicate the level of limitation **during the last month**. If the activity has been completely avoided because it is too difficult, then circle '10'. If you avoid an activity for reasons other than pain or difficulty, leave the item blank.

|  |  | No  limitation | | | | | Severe  limitation | | | | | |
| --- | --- | --- | --- | --- | --- | --- | --- | --- | --- | --- | --- | --- |
| 1. | Chew tough food | 0 | 1 | 2 | 3 | 4 | 5 | 6 | 7 | 8 | 9 | 10 |
| 2. | Chew hard bread | 0 | 1 | 2 | 3 | 4 | 5 | 6 | 7 | 8 | 9 | 10 |
| 3. | Chew chicken (e.g., prepared in oven) | 0 | 1 | 2 | 3 | 4 | 5 | 6 | 7 | 8 | 9 | 10 |
| 4. | Chew crackers | 0 | 1 | 2 | 3 | 4 | 5 | 6 | 7 | 8 | 9 | 10 |
| 5. | Chew soft food (e.g., macaroni, canned or soft fruits, cooked vegetables, fish) | 0 | 1 | 2 | 3 | 4 | 5 | 6 | 7 | 8 | 9 | 10 |
| 6. | Eat soft food requiring no chewing (e.g.,mashed potatoes, apple sauce, pudding, pureed food) | 0 | 1 | 2 | 3 | 4 | 5 | 6 | 7 | 8 | 9 | 10 |
| 7. | Open wide enough to bite from a whole apple | 0 | 1 | 2 | 3 | 4 | 5 | 6 | 7 | 8 | 9 | 10 |
| 8. | Open wide enough to bite into a  sandwich | 0 | 1 | 2 | 3 | 4 | 5 | 6 | 7 | 8 | 9 | 10 |
| 9. | Open wide enough to talk | 0 | 1 | 2 | 3 | 4 | 5 | 6 | 7 | 8 | 9 | 10 |
| 10. | Open wide enough to drink from a cup | 0 | 1 | 2 | 3 | 4 | 5 | 6 | 7 | 8 | 9 | 10 |
| 11. | Swallow | 0 | 1 | 2 | 3 | 4 | 5 | 6 | 7 | 8 | 9 | 10 |
| 12. | Yawn | 0 | 1 | 2 | 3 | 4 | 5 | 6 | 7 | 8 | 9 | 10 |
| 13. | Talk | 0 | 1 | 2 | 3 | 4 | 5 | 6 | 7 | 8 | 9 | 10 |
| 14. | Sing | 0 | 1 | 2 | 3 | 4 | 5 | 6 | 7 | 8 | 9 | 10 |
| 15. | Putting on a happy face | 0 | 1 | 2 | 3 | 4 | 5 | 6 | 7 | 8 | 9 | 10 |
| 16. | Putting on an angry face | 0 | 1 | 2 | 3 | 4 | 5 | 6 | 7 | 8 | 9 | 10 |
| 17. | Frown | 0 | 1 | 2 | 3 | 4 | 5 | 6 | 7 | 8 | 9 | 10 |
| 18. | Kiss | 0 | 1 | 2 | 3 | 4 | 5 | 6 | 7 | 8 | 9 | 10 |
| 19. | Smile | 0 | 1 | 2 | 3 | 4 | 5 | 6 | 7 | 8 | 9 | 10 |
| 20 | Laugh | 0 | 1 | 2 | 3 | 4 | 5 | 6 | 7 | 8 | 9 | 10 |

**APPENDIX C. Depression Anxiety Stress Scale-21 (DASS-21)**

Please read each statement and circle a number 0, 1, 2 or 3 which indicates how much the statement applied to you over the past week. There are no right or wrong answers. Do not spend too much time on any statement.

The rating scale is as follows:

0 Did not apply to me at all

1 Applied to me to some degree, or some of the time

2 Applied to me to a considerable degree, or a good part of time

3 Applied to me very much, or most of the time

|  |  | Never | Sometimes | Often | Almost Always |
| --- | --- | --- | --- | --- | --- |
| 1 | I found it hard to wind down | 0 | 1 | 2 | 3 |
| 2 | I was aware of dryness of my mouth | 0 | 1 | 2 | 3 |
| 3 | I couldn’t seem to experience any positive feeling at all | 0 | 1 | 2 | 3 |
| 4 | I experienced breathing difficulty (e.g., excessively rapid breathing, breathlessness in the absence of physical exertion) | 0 | 1 | 2 | 3 |
| 5 | I found it difficult to work up the initiative to do things | 0 | 1 | 2 | 3 |
| 6 | I tended to over-react to situations | 0 | 1 | 2 | 3 |
| 7 | I experienced trembling (e.g., in the hands) | 0 | 1 | 2 | 3 |
| 8 | I felt that I was using a lot of nervous energy | 0 | 1 | 2 | 3 |
| 9 | I was worried about situations in which I might panic and make a fool of myself | 0 | 1 | 2 | 3 |
| 10 | I felt that I had nothing to look forward to | 0 | 1 | 2 | 3 |
| 11 | I found myself getting agitated | 0 | 1 | 2 | 3 |
| 12 | I found it difficult to relax | 0 | 1 | 2 | 3 |
| 13 | I felt down-hearted and blue | 0 | 1 | 2 | 3 |
| 14 | I was intolerant of anything that kept me from getting on with what I was doing | 0 | 1 | 2 | 3 |
| 15 | I felt I was close to panic | 0 | 1 | 2 | 3 |
| 16 | I was unable to become enthusiastic about anything | 0 | 1 | 2 | 3 |
| 17 | I felt I wasn’t worth much as a person | 0 | 1 | 2 | 3 |
| 18 | I felt that I was rather touchy | 0 | 1 | 2 | 3 |
| 19 | I was aware of the action of my heart in the absence of physical exertion (e.g.,sense of heart rate increase, heart missing a beat) | 0 | 1 | 2 | 3 |
| 20 | I felt scared without any good reason | 0 | 1 | 2 | 3 |
| 21 | I felt that life was meaningless | 0 | 1 | 2 | 3 |

**APPENDIX D. Pittsburgh Sleep Quality Index (PSQI)**

Instructions: The following questions relate to your usual sleep habits during the past month only. Your answers should indicate the most accurate reply for the majority of days and nights in the past month. Please answer all questions.

1. During the past month, what time have you usually gone to bed at night?

BED TIME ___________

2. During the past month, how long (in minutes) has it usually taken you to fall asleep each night?

NUMBER OF MINUTES___________

1. During the past month, what time have you usually gotten up in the morning?

GETTING UP TIME___________

4.During the past month, how many hours of actual sleep did you get at night? (This may be different than the number of hours you spent in bed.)

HOURS OF SLEEP PER NIGHT___________

**For each of the remaining questions, check the one best response. Please answer all questions.**

5. During the past month, how often have you had trouble sleeping because you.

a) Cannot get to sleep within 30 minutes

□None □Less than once a week

□Once or twice a week □Three or more a week

b) Wake up in the middle of the night or early morning

□None □Less than once a week

□Once or twice a week □Three or more a week

c) Have to get up to use the bathroom

□None □Less than once a week

□Once or twice a week □Three or more a week

d) Cannot breathe comfortably

□None □Less than once a week

□Once or twice a week □Three or more a week

e) Cough or snore loudly

□None □Less than once a week

□Once or twice a week □Three or more a week

f) Feel too cold

□None □Less than once a week

□Once or twice a week □Three or more a week

g) Feel too hot

□None □Less than once a week

□Once or twice a week □Three or more a week

h) Had bad dreams

□None □Less than once a week

□Once or twice a week □Three or more a week

i) Have pain

□None □Less than once a week

□Once or twice a week □Three or more a week

j) Other reason(s), please describe__________________________

□None □Less than once a week

□Once or twice a week □Three or more a week

6. During the past month, how would you rate your sleep quality overall?

□Very good □Fairly good

□Fairly bad □Very bad

7. During the past month, how often have you taken medicine to help you sleep (prescribed or "over the counter")?

□None □Less than once a week

□Once or twice a week □Three or more a week

8. During the past month, how often have you had trouble staying awake while driving, eating meals, or engaging in social activity?

□None □Less than once a week

□Once or twice a week □Three or more a week

1. During the past month, how much of a problem has it been for you to keep up enough enthusiasm to get things done?

□No problem at all □Only a very slight problem

□Somewhat of a problem □A very big problem

1. Do you have a bed partner or roommate?

□No bed partner or roommate □Partner/roommate in other room

□Partner in same room, but not same bed □Partner in same bed

If you have a roommate or bed partner, ask him/her how often in the past month you have had . . .

a) Loud snoring

□None □Less than once a week

□Once or twice a week □Three or more a week

b) Long pauses between breaths while asleep

□None □Less than once a week

□Once or twice a week □Three or more a week

c) Legs twitching or jerking while you sleep

□None □Less than once a week

□Once or twice a week □Three or more a week

d) Episodes of disorientation or confusion during sleep

□None □Less than once a week

□Once or twice a week □Three or more a week

e) Other restlessness while you sleep: please describe ___________________

□None □Less than once a week

□Once or twice a week □Three or more a week
