# Supplementary material for: Effect of acupuncture for temporomandibular disorders: a randomized clinical trial
Source: QJM. 2024 May 6;117(9):647–56. doi: 10.1093/qjmed/hcae094 (PMC11537310; doi:10.1093/qjmed/hcae094)

**SUPPLEMENT 2: SUPPLEMENTARY MATERIAL**

**Effect of acupuncture for temporomandibular disorders: A randomized clinical trial**

**Table of Contents**

[Summary of Protocol Amendments 2](#_Toc20261)

[Inclusion and Exclusion Criteria 3](#_Toc20261)

[Diagnostic Criteria of Temporomandibular disorders (DC/TMD) 5](#_Toc27667)

[Definitions 8](#_Toc26692)

[Acupuncture Ritual 9](#_Toc31986)

[Table S1. Location of acupoints 11](#_Toc23706)

[Table S2. Similarities and differences between the two groups 12](#_Toc30781)

[Table S3. The schedule of enrolment, interventions, and assessments 13](#_Toc31344)

[Table S4. Reasons for excluding 56 participants before randomization 14](#_Toc9655)

[Table S5. Demographic and baseline clinical characteristics in the pre-protocol population. 15](#_Toc15023)

[Table S6. Primary, secondary, and exploratory clinical efficacy outcomes during the treatment and follow-up periods in the per-protocol population 18](#_Toc29195)

[Table S7. Participants' satisfaction at week 4 23](#_Toc31006)

[Table S8. Acupuncture Expectancy Scale Score at baseline. 24](#_Toc27809)

[Table S9. Compliance data at week 4 25](#_Toc1873)

[Table S10. Participant-Blinding Assessment Results 25](#_Toc1873)

[Table S11. Potentially clinically significant abnormalities in clinical laboratory outcomes 27](#_Toc26950)

[Table S12. Abnormalities for vital signs at any time 28](#_Toc14406)

[Figure S1. Study design. 29](#_Toc3036)

[Figure S2. Location of acupoints 30](#_Toc12745)

[Figure S3. Normal guide device and Park sham device 31](#_Toc30373)

[Figure S4. The sites of PPT measurement 32](#_Toc31251)

[Figure S5. The sites of sEMG measurement 33](#_Toc22517)

[Figure S6. Proportion of patients with ≥30% reduction in weakly pain intensity during treatment and follow-up periods 34](#_Toc11070)

[Figure S7. Proportion of patients with ≥50% reduction in weakly pain intensity during treatment and follow-up periods 35](#_Toc14914)

# Summary of Protocol Amendments

**Protocol Changes Version 1.0 (Oct. 17, 2018) to 1.1 (Mar. 5, 2019)**

- Exclusion criteria

Patients received concomitant therapy for managing facial pain is added.

- Study design

The screening period is added.

- Secondary Outcomes

≥30% and ≥50% reduction in mean weekly pain intensity are added.

The jaw opening and movement is added.

- Exploratory Outcomes

Secondary outcomes during weeks 5-8 are retitled as exploratory outcomes.

# Inclusion and Exclusion Criteria

**Inclusion Criteria**

Patients were eligible to be included in the study only if they met all of the following criteria at screening:

• be male or female, aged between 18 and 80 years;

• Suffer from pain-related TMD in accordance with DC/TMD, including myalgia (local myalgia, myofascial pain, myofascial pain with referral), arthralgia and pain attributed to TMD;

• Suffer from pain for at least three months;

• Provide a signed and dated informed consent form.

**Exclusion Criteria**

Patients were excluded from study enrolment if they met any of the following criteria at screening:

**Diagnostics Assessments:**

• Meet the diagnosis of disc displacement with reduction and locking, disc displacement without reduction and limitation of mouth opening, disc displacement without reduction without limitation of mouth opening, degenerative joint disease, and subluxation according to DC/TMD;

**Prior/Concomitant Therapy:**

• Have commenced occlusal splint therapy for the management of facial pain within 1 month prior to the screening period;

• Used any injection therapy (e.g., tender or trigger point injections, steroid injections) for the management of pain within 1 month prior to the screening period;

• Used acupuncture, biofeedback, or TENS for the management of pain within 1 month prior to the screening period;

• Used TMD-specific medicine (corticosteroids, benzodiazepines, muscle relaxants, opioids, antidepressants, and anticonvulsants) intervention within 1 month prior to the screening and baseline period.

**Medical Conditions:**

• Suffer from pain of dental origin, trauma, sinus pathology, neuropathic origin, inflammatory systemic diseases and cancer;

• Have a history of rheumatoid arthritis;

• Patients with mental illness or substance abuse;

• Be pregnant, lactating, or planning to become pregnant during the study.

# Diagnostic Criteria of Temporomandibular disorders (DC/TMD)

For further details, refer to DC/TMD diagnostic criteria.

**1.1 Myalgia (ICD-9729.1; ICD-10M79.1)**

Pain of muscle origin that is affected by jaw movement , function, or parafunction, and replication of this pain occurs with provocation testing of the masticatory muscles.

1. History: Positive for both of the following:
2. Pain in the jaw, temple, in the ear, or in front of ear; AND
3. Pain modified with jaw movement, function or parafunction.
4. Exam: Positive for both of the following:

1. Confirmation of pain location(s) in the temporalis or masseter muscle(s); AND

2. Report of familiar pain in the temporalis or masseter muscle(s) with at least one of the following provocation tests:

a. Palpation of the temporalis or masseter muscle(s); OR

b. Maximum unassisted or assisted opening movement(s).

**1.1.1 Local myalgia (ICD-9729.1; ICD-10M79.1)**

Pain of muscle origin as described for myalgia with localization of pain only at the site of palpation when using the myofascial examination protocol.

1. History: Positive for both of the following:
2. Pain in the jaw, temple, in the ear, or in front of ear; AND
3. Pain modified with jaw movement, function or parafunction.
4. Exam: Positive for all of the following:
5. Confirmation of pain location(s) in the temporalis or masseter muscle(s); AND
6. Report of familiar pain with palpation of the temporalis or masseter muscle(s); AND
7. Report of pain localized to the site of palpation.

**1.1.2 Myofascial pain (ICD-9729.1; ICD-10M79.1)**

Pain of muscle origin as described for myalgia with pain spreading beyond the site of palpation but within the boundary of the muscle when using the myofascial examination protocol.

1. History: Positive for both of the following:
2. Pain in the jaw, temple, in the ear, or in front of ear; AND
3. Pain modified with jaw movement, function or parafunction.
4. Exam: Positive for all of the following:
5. Confirmation of pain location(s) in the temporalis or masseter muscle(s); AND
6. Report of familiar pain with palpation of the temporalis or masseter muscle(s); AND
7. Report of pain spreading beyond the site of palpation but within the boundary of the muscle.

**1.1.3 Myofascial pain with referral (ICD-9729.1)**

Pain of muscle origin as described for myalgia with referral of pain beyond the boundary of the muscle being palpated when using the myofascial examination protocol. Spreading pain may also be present.

1. History: Positive for both of the following:
2. Pai in the jaw, temple, in the ear, or in front of ear; AND
3. Pain modified with jaw movement, function or parafunction.
4. Exam: Positive for all of the following:
5. Confirmation of pain location(s) in the temporalis or masseter muscle(s); AND
6. Report of familiar pain with palpation of the temporalis or masseter muscle(s); AND
7. Report of pain at a site beyond the boundary of the muscle being palpated.

**1.2 Arthralgia (ICD-9524.62; ICD-10M26.62)**

Pain of joint origin that is affected by jaw movement, function, or parafunction, and replication of this pain occurs with provocation testing of the TMJ.

1. History: Positive for both of the following:
2. Pain in the jaw, temple, in the ear, or in front of ear; AND
3. Pain modified with jaw movement, function or parafunction.
4. Exam: Positive for both of the following:

1. Confirmation of pain location in the area of the TMJ(s); AND

2. Report of familiar pain in the TMJ with at least one of the following provocation tests:

a. Palpation of the lateral pole or around the lateral pole; OR

b. Maximum unassisted or assisted opening, right or left lateral movements, or protrusive movement(s).

**1.3 Headache attributed to TMD (ICD-9339.89 and 748.0; ICD-10G44.89)**

Headache in the temple area secondary to pain-related TMD (see note) that is affected by jaw movement, function, or parafunction, and replication of this headache occurs with provocation testing of the masticatory system.

1. History: Positive for both of the following:
2. Headache of any type in the temple; AND
3. Headache modified with jaw movement, function or parafunction.
4. Exam: Positive for both of the following:
5. Confirmation of headache location in the area of the temporalis muscle (s); AND
6. Report of familiar headache in the temple area with at least one of the following provocation tests:
7. Palpation of the temporalis muscle(s); OR
8. Maximum unassisted or assisted opening, right or left lateral, or protrusive movement(s).

# Definitions

1. **Response Rates**

Proportion of patients with ≥50% and ≥30% reduction from baseline in weakly pain intensity in each period.

1. **Pain-free jaw opening**

Pain-free opening are measured by the interincisal distance between the maxillary and mandibular reference teeth.

1. **Maximum unassisted jaw opening**

Maximum unassisted opening are measured by the interincisal distance between the maxillary and mandibular reference teeth. Ask the patient about any pain produced by measuring maximum unassisted jaw opening.

1. **Maximum assisted jaw opening**

The examiner uses moderate pressure, push the mouth open further, measures the interincisal distance between the maxillary and mandibular teeth.

1. **Lateral movement**

Lateral excursive measurements are made between the maxillary and mandibular reference midlines, while protrusive excursive measurement is made between the labial surfaces of the maxillary and mandibular reference teeth.

1. **Protrusion movement**

Ask patient to move the mandible forward. Record any reported pain. Note that if the mandibular incisors cannot be protruded beyond the maxillary incisors, the value will be negative. If the incisors exhibit a Class III (anterior cross-bite situation exists) situation in maximum closure, the horizontal overlap is recorded as a negative value. The protrusive movement measured as the distance from the labial surfaces of the maxillary to mandibular incisors, will be recorded as a positive number.

# Acupuncture Ritual

We set up standardized ritual operations as follows:

**Step 1**: The patient is guided to a separate, quiet room. An acupuncturist meets the patient and informs him/her to receive a 30-minute duration of acupuncture treatment.

“Hello! I will give you a 30-minute duration of acupuncture treatment. Here are the acupuncture needles I am going to use. The acupuncture device will be used to determine the location of acupoints. Now you can rest for a while.”

**Acupuncture group**

Place the normal guide device at the acupoints and attach it to the skin with the self-adhesive pad.

**Sham acupuncture group**

Place the Park sham device at the acupoints and attach it to the skin with the self-adhesive pad.

**Step 2**: After the patient is completely relaxed, the acupuncturist asks the patient to stay in a fixed position.

“Please lie flat on the bed. The acupoints are distributed in the face, and upper and lower extremity.”

**Step 3**: Start the acupuncture treatment and insert the needle.

“Now I’m going to start the acupuncture treatment. Please relax yourself.”


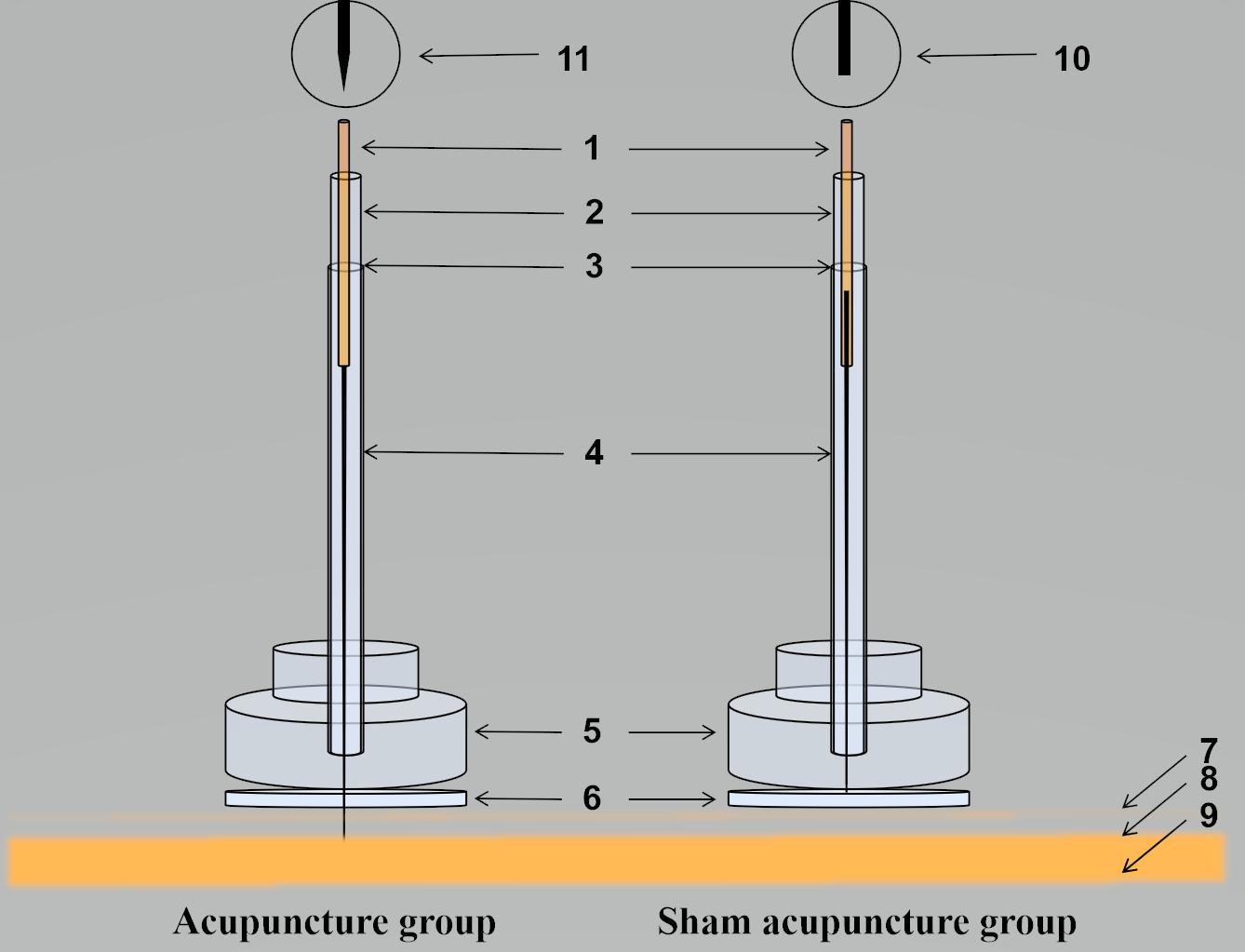


1. Needle handle, 2. Guide tube, 3. Guide o-ring, 4. Park tube, 5. Flange, 6. Adhesive pad, 7. Skin, 8. Dermis, 9. Muscle, 10. Blunt tip of sham needle, 11. Sharp tip of real needle.

After sterilization, sham needles with blunt tip were used. When they were fixed on the adhesive pad through Park sham device, patients felt a dull sensation. However, instead of penetrating the skin, the needles slide within its handle when they were pressed against the skin. We formulated and followed standardized step-by-step instructions and operations to use the same rituals in the manual acupuncture and sham acupuncture groups as far as possible.

**Step 4**: Start the first manipulation after all the needles are inserted into the appropriate place.


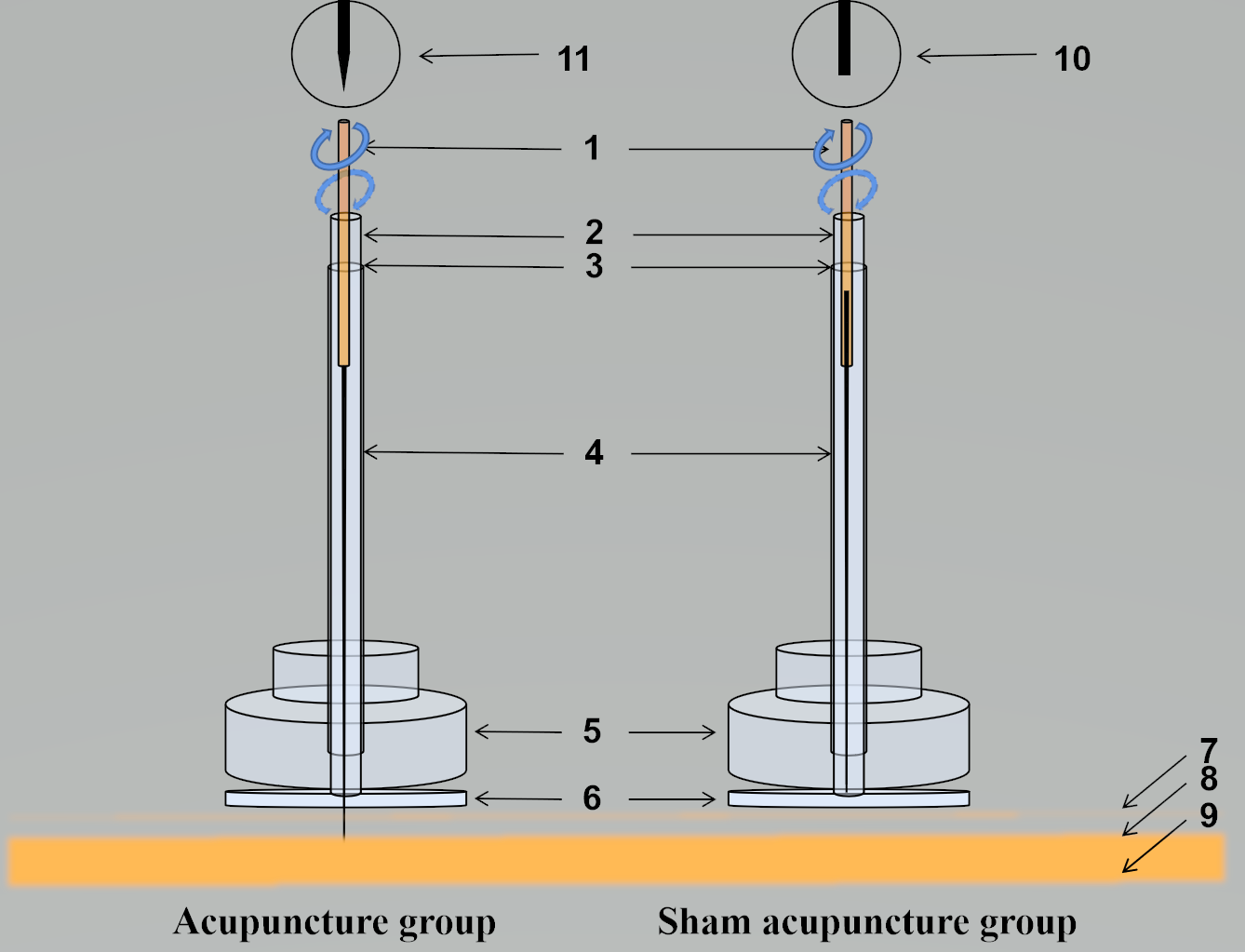


“The needle has been inserted smoothly. Now I will rotate each needle for a while. This is called “acupuncture manipulation”, which is a routine in traditional acupuncture treatment. A total of four times during the treatment will be performed. Now it’s the first time of acupuncture manipulation.”

The manipulation of each needle lasts 10 seconds and is repeated a total of 4 times with an interval of 10 minutes.

**Step 5**: Start the second manipulation.

“Now it’s the second time of acupuncture manipulation.”

**Step 6**: Start the third manipulation.

“Now it’s the third time of acupuncture manipulation.”

**Step 7**: Start the fourth manipulation and then finish the treatment.

“Now it’s the fourth time of acupuncture manipulation. Then the acupuncture treatment is over and I would pull the needle out. (After the needle out) Okay, now you can get up. Do you have any discomfort?”

| **Table S1. Location of acupoints** | |
| --- | --- |
| **Kinds of acupoint** | **Acupoints** |
| Bilateral acupoints | Hegu (LI4)，Yanglingquan (GB34) |
| Acupoints in the affected side(s) | Tinggong (SI19)，Jiache (ST6)，Xiaguan (ST7) |

| **Table S2. Similarities and differences between the two groups** | | |
| --- | --- | --- |
|  | **Acupuncture group** | **Sham acupuncture group** |
| Kinds of points stimulated | Acupoints | Acupoints |
| Needle | Real acupuncture needle | Sham acupuncture needle |
| Acupuncture device | Normal guide device | Park sham device |
| Number of acupoints | 7 or 10 | 7 or 10 |
| Depth of penetration, mm | 10-15 | 0 |
| Deqi | Yes | No |
| Needle retention, min | 30 | 30 |
| Number of sessions | 12 | 12 |

| **Table S3. The schedule of enrolment, interventions, and assessments** | | | | | | | | |
| --- | --- | --- | --- | --- | --- | --- | --- | --- |
| **STUDY PERIOD** | | | | | | | | |
|  | **Screening** | **Baseline** | **Allocation** | **Treatment** | | | | **Follow-up** |
| **TIMEPOINT**  **(W, week)** | -2 | -1 | 0 | 1 | 2 | 3 | 4 | 8 |
| **Enrolment** | | | | | | | | |
| Informed consent | **×** |  |  |  |  |  |  |  |
| Eligibility criteria | **×** | **×** |  |  |  |  |  |  |
| Demography Characteristics |  | **×** |  |  |  |  |  |  |
| Disease history of TMD |  | **×** |  |  |  |  |  |  |
| Randomization |  |  | **×** |  |  |  |  |  |
| **Interventions** | | | | | | | | |
| Acupuncture group |  |  |  |  | | | |  |
| Sham acupuncture group |  |  |  |  | | | |  |
| **Assessments** | | | | | | | | |
| TMD diary |  |  | | | | | | |
| VAS |  |  | **×** |  |  |  | **×** | **×** |
| Jaw opening and movement |  |  | **×** |  |  |  | **×** | **×** |
| GCPS |  |  | **×** |  |  |  | **×** | **×** |
| JFLS-20 |  |  | **×** |  |  |  | **×** | **×** |
| DASS-21 |  |  | **×** |  |  |  | **×** | **×** |
| PSQI |  |  | **×** |  |  |  | **×** | **×** |
| PPT |  |  | **×** |  |  |  | **×** |  |
| sEMG |  |  | **×** |  |  |  | **×** |  |
| Laboratory test* |  |  | **×** |  |  |  | **×** |  |
| Acupuncture expectancy scale |  |  | **×** |  |  |  |  |  |
| Participant’s satisfaction scale |  |  |  |  |  |  | **×** |  |
| Participant’s compliance |  |  |  | **×** | **×** | **×** | **×** |  |
| Blinding assessment |  |  | **×** |  |  |  | **×** |  |
| clinical laboratory tests |  |  | **×** |  |  |  | **×** |  |
| Vital signs |  |  | **×** |  |  |  | **×** | **×** |
| Weight |  |  | **×** |  |  |  | **×** | **×** |
| Adverse events |  |  |  | | | | | |
| VAS: visual analogue scale; GCPS: Graded Chronic Pain Scale; JFLS-20: Jaw Function Limitation Scale; DASS-21: Depression Anxiety and Stress Scale-21; PSQI: Pittsburgh Sleep Quality Index.  *The laboratory test includes blood and urine samples for the clinical safety. | | | | | | | | |

| **Table S4. Reasons for excluding 56 participants before randomization** | |
| --- | --- |
| **Reasons for exclusion** | **Numbers** |
| **Did not meet eligible criteria**^*^ | 47 |
| Diagnosis based on DC/TMD | 19 |
| Aged between 18 and 80 years | 3 |
| Suffering from pain for at least 3 months before screening | 7 |
| Had commenced occlusal splint therapy within 1 month prior to the screening period | 2 |
| Had used any injection therapy within 1 month prior to the screening period | 3 |
| Had used acupuncture, biofeedback, or TENS within 1 month prior to the screening period | 5 |
| Had used TMD-specific medication within 1 month prior to the screening and baseline period | 8 |
| Suffer from pain of dental origin, trauma, sinus pathology, neuropathic origin, inflammatory systemic diseases, cancer | 2 |
| Had other medical conditions | 6 |
| Pregnant, or lactating, or planning to become pregnant during the study | 1 |
| **Declined to participate** | 5 |
| **Withdrew by participants during baseline period** | 3 |
| **Lost to follow-up** | 1 |

^*^Some participants had >1 reason for exclusion.

| **Table S5. Demographic and baseline clinical characteristics in the pre-protocol population.** | | |
| --- | --- | --- |
| **Characteristics** | **Acupuncture group (n=29)** | **Sham acupuncture** group **(n=28)** |
| **Demographics** |  |  |
| **Age, mean (SD), years** | 42.8 (15.6) | 45.8 (17.7) |
| **Sex, no. (%)** |  |  |
| Female | 25 (86.2) | 25 (89.3) |
| Male | 4 (13.8) | 3 (10.7) |
| **Body-mass index, kg/m^2^** | 22.2 (3.3) | 21.9 (2.5) |
| **Current employment status** |  |  |
| Employed | 18 (62.1) | 18 (64.3) |
| Student | 5 (17.2) | 3 (10.7) |
| Unemployed, retired | 6 (20.7) | 7 (25.0) |
| **Education** |  |  |
| ≤High school | 6 (20.7) | 3 (10.7) |
| College | 19 (65.5) | 20 (71.4) |
| Graduate degree | 4 (13.8) | 5 (17.9) |
| **Marital status** |  |  |
| Never married | 8 (27.6) | 10 (35.7) |
| Married | 19 (65.5) | 17 (60.7) |
| Divorced | 2 (6.9) | 1 (3.6) |
| **Annual income (CNY)** |  |  |
| ¥100,000 or less | 6 (20.7) | 12 (42.9) |
| ¥100,000 – ¥200,000 | 10 (34.5) | 7 (25.0) |
| ¥200,000 – ¥500,000 | 12 (41.4) | 9 (32.1) |
| >¥500,000 | 1 (3.4) | 0 (0.0) |
| **Disease characteristics during the baseline** |  |  |
| **Facial pain** |  |  |
| Time since onset, years | 2.2 (2.8) | 2.0 (2.7) |
| Weekly pain intensity, mean (SD) | 5.3 (1.9) | 5.8 (2.1) |
| Painful days in the last 30 days | 16.3 (11.5) | 19.5 (11.4) |
| TMD-specific medication, n (%) | 6 (20.7) | 7 (25.0) |
| **DC/TMD examination findings** |  |  |
| TMD myalgia, n (%) | 22 (75.9) | 21 (75.0) |
| TMD arthralgia, n (%) | 27 (93.1) | 24 (85.7) |
| TMD headache, n (%) | 7 (24.1) | 6 (21.4) |
| **Jaw opening and movement** |  |  |
| Pain-free jaw opening, mm | 22.1 (10.1) | 24.5 (9.0) |
| Maximum unassisted jaw opening, mm | 28.8 (9.3) | 32.4 (6.8) |
| Maximum assisted jaw opening, mm | 32.1 (9.3) | 35.9 (6.0) |
| Protrusion movement, mm | 3.2 (2.1) | 3.7 (1.9) |
| Left lateral movement, mm | 5.1 (2.4) | 5.8 (1.9) |
| Right lateral movement, mm | 5.2 (2.5) | 5.9 (2.1) |
| **Graded Chronic Pain Scale (GCPS)** |  |  |
| Grades I-IIa, n (%) | 8 (27.6) | 9 (32.1) |
| Grades IIb-IV, n (%) | 21 (72.4) | 19 (67.9) |
| Characteristic pain intensity, 0–100 scale | 56.3 (21.5) | 58.7 (17.0) |
| Disability score, 0–100 scale | 34.5 (26.5) | 35.1 (27.8) |
| **Jaw Functional Limitations Scale-20 (JFLS-20)** |  |  |
| Mastication, 0-10 scale | 4.9 (3.5) | 4.8 (2.6) |
| Vertical jaw mobility, 0-10 scale | 4.3 (3.5) | 4.7 (2.2) |
| Verbal and emotional expression, 0-10 scale | 2.9 (4.1) | 2.5 (2.4) |
| Overall, 0-10 scale | 4.0 (3.5) | 4.0 (2.1) |
| **Depression, Anxiety and Stress Scales (DASS-21)** |  |  |
| Depression | 4.6 (2.6) | 4.9 (2.4) |
| Anxiety | 4.0 (2.9) | 4.8 (3.1) |
| Stress | 5.2 (3.7) | 5.1 (4.0) |
| Overall | 13.8 (7.2) | 14.9 (8.1) |
| **Pittsburgh sleep quality index (PSQI)** |  |  |
| Subjective sleep quality | 1.2 (0.8) | 1.3 (0.8) |
| Sleep latency | 1.3 (1.2) | 1.5 (0.9) |
| Sleep duration | 1.1 (0.9) | 1.1 (0.9) |
| Habitual sleep efficiency | 0.7 (1.0) | 0.7 (1.0) |
| Sleep disturbances | 1.2 (0.6) | 1.5 (0.7) |
| Use of sleep medication | 0.3 (0.8) | 0.7 (1.2) |
| Daytime dysfunction | 1.5 (1.0) | 1.8 (1.1) |
| Overall | 7.3 (3.8) | 8.6 (4.2) |
| **Quantitative sensory testing** |  |  |
| **Pressure pain thresholds (PPTs)** |  |  |
| Masseter PPT, 0-500 kPa | 123.7 (49.1) | 130.3 (43.7) |
| Anterior temporalis PPT, 0-500 kPa | 140.0 (62.5) | 143.6 (44.5) |
| Sternocleidomastoid PPT, 0-500 kPa | 114.6 (40.9) | 111.5 (38.2) |
| Trapezius PPT, 0-500 kPa | 161.3 (66.5) | 178.7 (62.7) |
| TMJ PPT, 0-500 kPa | 118.9 (44.6) | 129.2 (42.5) |
| **sEMG** |  |  |
| **Mandibular resting position (MR)** |  |  |
| RMS, Masseter muscle, left, μV | 10.6 (15.9) | 14.9 (28.6) |
| RMS, Masseter muscle, right, μV | 13.3 (19.9) | 15.5 (23.7) |
| RMS, Anterior temporalis muscle, left, μV | 15.5 (14.2) | 17.9 (25.0) |
| RMS, Anterior temporalis muscle, right, μV | 8.7 (5.6) | 11.6 (11.6) |
| **Habitual chewing (HC)** |  |  |
| RMS, Masseter muscle, left, μV | 97.4 (92.3) | 66.3 (46.4) |
| RMS, Masseter muscle, right, μV | 100.7 (118.3) | 70.9 (49.4) |
| RMS, Anterior temporalis muscle, left, μV | 103.5 (68.7) | 79.7 (59.3) |
| RMS, Anterior temporalis muscle, right, μV | 103.9 (63.0) | 83.9 (47.2) |
| **Maximal voluntary contraction (MVC)** |  |  |
| RMS, Masseter muscle, left, μV | 69.6 (45.6) | 60.1 (43.4) |
| RMS, Masseter muscle, right, μV | 78.4 (78.0) | 54.9 (28.1) |
| RMS, Anterior temporalis muscle, left, μV | 57.0 (36.2) | 54.1 (38.3) |
| RMS, Anterior temporalis muscle, right, μV | 56.1 (32.9) | 58.5 (31.3) |
| Data are mean (SD) or n (%). SD=standard deviation; CNY=China Yuan; DC/TMD=diagnostic criteria for temporomandibular disorder; GCPS=Graded Chronic Pain Scale; JFLS=Jaw Functional Limitation Scale; DASS=Depression, Anxiety and Stress Scales; PSQI=Pittsburgh Sleep Quality Index; PPT=pressure pain threshold; TMJ=temporomandibular joint; sEMG=Surface electromyography; RMS=Root Mean Square; MR=Mandibular resting position; HC=Habitual chewing; MVC=Maximal voluntary contraction. | | |

| **Table S6. Primary, secondary, and exploratory clinical efficacy outcomes during the treatment and follow-up periods in the per-protocol population** | | | | | | |
| --- | --- | --- | --- | --- | --- | --- |
|  | **At week 4** | | | **At week 8 (Exploratory outcomes)** | | |
|  | **Acupuncture group (n=29)** | **Sham acupuncture** group **(n=28)** | Difference or odds ratio (95% CI) | **Acupuncture group (n=29)** | **Sham acupuncture** group **(n=28)** | Difference or odds ratio (95% CI) |
| **Primary outcome** |  |  |  |  |  |  |
| Change from baseline in mean weekly pain intensity | -3.0 (0.3) | -1.5 (0.4) | -1.62 (-2.48 to -0.76) ^*^ | -3.8 (0.3) | -2.7 (0.4) | -1.43 (-2.25 to -0.62) ^*^ |
| **Secondary outcomes** |  |  |  |  |  |  |
| ≥30% reduction in mean weekly pain intensity | 86.2 (6.5) | 39.3 (9.4) | 12.44 (3.19 to 61.54) ^*^ | 96.6 (3.4) | 75.0 (8.3) | 10.85 (1.52 to 62.18) ^*^ |
| ≥50% reduction in mean weekly pain intensity | 55.2 (9.4) | 17.9 (7.4) | 8.61 (2.21 to 43.93) ^*^ | 86.2 (6.5) | 57.1 (9.5) | 5.37 (1.43 to 24.42) ^*^ |
| **Jaw opening and movement** |  |  |  |  |  |  |
| Change from baseline in pain-free jaw opening, mm | 5.6 (0.7) | 2.3 (0.4) | 3.24 (1.67 to 4.81) ^*^ | 5.1 (0.4) | 2.0 (0.3) | 2.98 (1.87 to 4.08) ^*^ |
| Change from baseline in maximum unassisted jaw opening, mm | 3.4 (0.5) | 1.6 (0.3) | 1.99 (0.97 to 3.01) ^*^ | 3.0 (0.4) | 1.5 (0.3) | 1.51 (0.67 to 2.34) ^*^ |
| Change from baseline in maximum assisted jaw opening, mm | 2.8 (0.4) | 1.3 (0.2) | 1.60 (0.67 to 2.52) ^*^ | 3.1 (0.4) | 1.2 (0.2) | 2.03 (1.17 to 2.90) ^*^ |
| Change from baseline in protrusion movement, mm | 1.3 (0.2) | 0.4 (0.1) | 0.95 (0.52, to 1.38) ^*^ | 1.6 (0.2) | 0.2 (0.1) | 1.32 (0.81 to 1.83) ^*^ |
| Change from baseline in left lateral movement, mm | 1.2 (0.2) | 0.5 (0.1) | 0.77 (0.35 to 1.18) ^*^ | 1.1 (0.2) | 0.1 (0.1) | 1.01 (0.63 to 1.39) ^*^ |
| Change from baseline in right lateral movement, mm | 1.4 (0.2) | 0.2 (0.1) | 1.12 (0.65 to 1.59) ^*^ | 1.1 (0.2) | 0.2 (0.1) | 0.87 (0.43 to 1.30) ^*^ |
| **Graded Chronic Pain Scale (GCPS)** |  |  |  |  |  |  |
| Change from baseline in characteristic pain intensity, 0–100 scale | -27.2 (4.2) | -15.5 (1.9) | -11.83 (-21.38 to -2.27) ^*^ | -24.3 (3.1) | -10.9 (2.7) | -13.60 (-22.32 to -4.87) ^*^ |
| Change from baseline in disability score, 0–100 scale | -22.6 (4.4) | -8.5 (3.5) | -15.20 (-26.79 to -3.62) ^*^ | -20.4 (4.7) | -7.8 (1.7) | -12.90 (-23.65 to -2.15) ^*^ |
| **Jaw Functional Limitations Scale-20 (JFLS-20)** |  |  |  |  |  |  |
| Change from baseline in mastication, 0-10 scale | -2.4 (0.3) | -0.9 (0.2) | -1.37 (-2.10 to -0.64) ^*^ | -2.4 (0.3) | -1.1 (0.2) | -1.29 (-1.94 to -0.64) ^*^ |
| Change from baseline in vertical jaw mobility, 0-10 scale | -2.3 (0.4) | -0.9 (0.2) | -1.44 (-2.38 to -0.50) ^*^ | -2.3 (0.3) | -1.0 (0.2) | -1.33 (-2.14 to -0.52) ^*^ |
| Change from baseline in verbal and emotional expression, 0-10 scale | -1.7 (0.3) | -0.7 (0.1) | -1.04 (-1.61 to -0.48) ^*^ | -1.8 (0.2) | -0.9 (0.2) | -0.87 (-1.47 to -0.27) ^*^ |
| Change from baseline in overall, 0-10 scale | -2.1 (0.3) | -0.8 (0.1) | -1.28 (-1.88 to -0.69) ^*^ | -2.2 (0.2) | -1.0 (0.1) | -1.16 (-1.64 to -0.68) ^*^ |
| **Depression, Anxiety and Stress Scales-21 (DASS-21)** |  |  |  |  |  |  |
| Change from baseline in depression | -2.2 (0.3) | -1.2 (0.3) | -0.94 (-1.74 to -0.15) ^*^ | -1.8 (0.3) | -0.9 (0.3) | -0.98 (-1.81 to -0.15) ^*^ |
| Change from baseline in anxiety | -1.8 (0.3) | -0.5 (0.3) | -1.16 (-1.99 to -0.34) ^*^ | -1.7 (0.4) | -0.2 (0.2) | -1.29 (-2.15 to -0.43) ^*^ |
| Change from baseline in stress | -2.1 (0.3) | -0.6 (0.4) | -1.47 (-2.60 to -0.35) ^*^ | -2.0 (0.4) | -0.4 (0.3) | -1.54 (-2.44 to -0.64) ^*^ |
| Change from baseline in overall | -6.0 (0.6) | -2.4 (0.6) | -3.58 (-5.47 to -1.69) ^*^ | -5.5 (0.8) | -1.5 (0.5) | -3.81 (-5.69 to -1.94) ^*^ |
| **Pittsburgh sleep quality index (PSQI)** |  |  |  |  |  |  |
| Change from baseline in subjective sleep quality | -0.2 (0.1) | 0.1 (0.1) | -0.35 (-0.68 to -0.02) ^*^ | -0.2 (0.1) | 0.0 (0.1) | -0.21 (-0.58 to 0.16) |
| Change from baseline in sleep latency | -0.4 (0.2) | 0.2 (0.2) | -0.72 (-1.19 to -0.26) ^*^ | -0.5 (0.2) | 0.0 (0.2) | -0.65 (-1.12 to -0.17) ^*^ |
| Change from baseline in sleep duration | -0.1 (0.2) | 0.0 (0.1) | -0.17 (-0.55 to 0.22) | -0.1 (0.2) | 0.0 (0.1) | -0.14 (-0.55 to 0.27) |
| Change from baseline in habitual sleep efficiency | -0.1 (0.2) | -0.1 (0.2) | -0.10 (-0.57 to 0.36) | -0.2 (0.2) | -0.0 (0.2) | -0.21 (-0.70 to 0.29) |
| Change from baseline in sleep disturbances | -0.2 (0.1) | 0.0 (0.1) | -0.21 (-0.60 to 0.17) | -0.3 (0.2) | -0.2 (0.1) | -0.13 (-0.59 to 0.33) |
| Change from baseline in use of sleep medication | -0.2 (0.1) | -0.2 (0.2) | 0.01 (-0.36 to 0.37) | -0.2 (0.1) | -0.0 (0.1) | -0.13 (-0.43 to 0.17) |
| Change from baseline in daytime dysfunction | -0.4 (0.1) | 0.1 (0.1) | -0.44 (-0.82 to -0.06) ^*^ | -0.6 (0.1) | 0.0 (0.2) | -0.49 (-0.90 to -0.08) ^*^ |
| Change from baseline in overall | -1.7 (0.5) | 0.2 (0.5) | -1.96 (-3.43 to -0.50) ^*^ | -2.0 (0.6) | -0.1 (0.5) | -1.93 (-3.37 to -0.49) ^*^ |
| **Quantitative sensory testing** |  |  |  |  |  |  |
| **Pressure pain thresholds (PPTs)** |  |  |  |  |  |  |
| Change from baseline in masseter PPT, 0-500 kPa | 3.5 (6.5) | 0.6 (4.6) | 4.03 (-12.55 to 20.60) | - | - | - |
| Change from baseline in anterior temporalis PPT, 0-500 kPa | 4.9 (8.0) | 0.2 (4.3) | 4.61 (-14.73 to 23.94) | - | - | - |
| Change from baseline in sternocleidomastoid PPT, 0-500 kPa | 3.2 (6.0) | 2.6 (7.1) | 0.62 (-19.08 to 20.32) | - | - | - |
| Change from baseline in trapezius PPT, 0-500 kPa | 27.8 (11.0) | 13.1 (6.1) | 16.17 (-8.71 to 41.05) | - | - | - |
| Change from baseline in TMJ PPT, 0-500 kPa | 26.1 (7.3) | 12.8 (6.0) | 12.75 (-6.60 to 32.10) | - | - | - |
| **sEMG** |  |  |  |  |  |  |
| **Mandibular resting position (MR)** |  |  |  |  |  |  |
| RMS, Change from baseline in masseter muscle, left, μV | -1.6 (3.6) | -3.0 (5.5) | 0.66 (-12.05 to 13.38) | - | - | - |
| RMS, Change from baseline in masseter muscle, right, μV | -3.8 (3.8) | -6.6 (4.7) | 1.25 (-10.97 to 13.47) | - | - | - |
| RMS, Change from baseline in anterior temporalis muscle, left, μV | -4.1 (2.9) | -8.5 (4.8) | 3.07 (-8.28 to 14.42) | - | - | - |
| RMS, Change from baseline in anterior temporalis muscle, right, μV | -0.5 (1.0) | -1.2 (2.9) | 0.13 (-5.99 to 6.25) | - | - | - |
| **Habitual chewing (HC)** |  |  |  |  |  |  |
| RMS, Change from baseline in masseter muscle, left, μV | -14.7 (12.6) | -12.3 (8.4) | -3.99 (-35.73 to 27.76) | - | - | - |
| RMS, Change from baseline in masseter muscle, right, μV | -12.7 (22.6) | -19.4 (9.3) | 7.22 (-42.82 to 57.27) | - | - | - |
| RMS, Change from baseline in anterior temporalis muscle, left, μV | -7.1 (11.3) | -10.6 (11.1) | 3.94 (-29.17 to 37.04) | - | - | - |
| RMS, Change from baseline in anterior temporalis muscle, right, μV | -3.1 (9.7) | -20.5 (9.1) | 18.51 (-9.42 to 46.43) | - | - | - |
| **Maximal voluntary contraction (MVC)** |  |  |  |  |  |  |
| RMS, Change from baseline in masseter muscle, left, μV | -14.8 (7.9) | 10.9 (11.9) | -24.72 (-52.58 to 3.13) | - | - | - |
| RMS, Change from baseline in masseter muscle, right, μV | -17.7 (15.4) | 3.5 (7.7) | -20.39 (-56.19 to 15.41) | - | - | - |
| RMS, Change from baseline in anterior temporalis muscle, left, μV | -9.4 (6.5) | -9.4 (7.1) | -1.23 (-20.63 to 18.16) | - | - | - |
| RMS, Change from baseline in anterior temporalis muscle, right, μV | 3.3 (8.4) | -16.8 (5.5) | 19.44 (-1.77 to 40.64) | - | - | - |
| Data are least squares mean (SE), mean difference (95% CI), mean percentage (SE), or odds ratio (95% CI). GCPS=Graded Chronic Pain Scale; JFLS=Jaw Functional Limitation Scale; DASS=Depression, Anxiety and Stress Scales; PSQI=Pittsburgh Sleep Quality Index; PPT=pressure pain threshold; TMJ=temporomandibular joint; sEMG=Surface electromyography; RMS=Root Mean Square; MR=Mandibular resting position; HC=Habitual chewing; MVC=Maximal voluntary contraction.  ^*^ P < 0.05 | | | | | | |

| **Table S**7. Participants’ satisfaction at week 4. Values are numbers (percentages) unless stated otherwise | | | |
| --- | --- | --- | --- |
| **At week 4** | | | |
|  | **Acupuncture group (n=30)** | Sham acupuncture group **(n=30)** | **P value^*^** |
| 5=extremely satisfied | 14 (46.7) | 9 (30.0) |  |
| 4=satisfied | 15 (50.0) | 11 (36.7) |  |
| 3=moderately satisfied | 1 (3.3) | 9 (30.0) |  |
| 2=dissatisfied | 0 (0) | 1 (3.3) |  |
| 1=extremely dissatisfied | 0 (0) | 0 (0) |  |
| Responder (score of 4-5) | 29 (96.7) | 20 (66.7) | 0.008 |
| ^*^Using χ^2^ test. | | | |

| **Table S8. Acupuncture Expectancy Scale Score at baseline.** Values are numbers (percentages) unless stated otherwise | | |
| --- | --- | --- |
|  | **Acupuncture group (n=30)** | Sham acupuncture group **(n=30)** |
| **Acupuncture expectation of improvement^*^** |  |  |
| Ineffective, n (%) | 0 (0) | 0 (0) |
| May be ineffective, n (%) | 1 (3.3) | 1 (3.3) |
| Unclear, n (%) | 2 (6.7) | 2 (6.7) |
| May be effective, n (%) | 12 (40.0) | 16 (53.3) |
| Effective, n (%) | 15 (50.0) | 11 (36.7) |

**^*^**The Fisher’s exact test was used to compare the expectations of treatment’s effect between the two groups (P=0.779)

| **Table S9. Compliance data at week 4.** Values are numbers (percentages) unless stated otherwise | | |
| --- | --- | --- |
|  | **Acupuncture group (n=30)** | Sham acupuncture group **(n=30)** |
| The number of treatment sessions received | 11.7 (0.7) | 11.3 (1.5) |
| Participants received at least 10 sessions of treatment (12 sessions in total, compliance rates ≥ 80%)^*^ | 29 (96.7) | 28 (93.3) |
| ^*^There was no significant difference between groups in the compliance of acupuncture (P=0.336). | | |

| **Table S10. Participant-Blinding Assessment Results. Values are numbers (percentages) unless stated otherwise** | | | | | |
| --- | --- | --- | --- | --- | --- |
|  | **Think in Acupuncture group (n=29)** | Think in Sham acupuncture group (n=28) | Did not know | P value^*^ | Bang blinding index (95%CI)† |
| **At week 4‡** |  |  |  |  |  |
| Acupuncture group (n=29) | 8 (27.6) | 2 (6.9) | 19 (65.5) | 0.871 | 0.21 (0.01, 0.41) |
| Sham acupuncture group (n=28) | 7 (25.0) | 3 (10.7) | 18 (64.3) |  | -0.14 (-0.36, 0.07) |

^*^Using χ^2^ test. †The Bang blinding index for each group represents the proportion of participants making a correct treatment guess beyond chance: 0 represents perfect blinding; a positive index indicates a correct guess, and a negative index indicates a guess in the opposite direction.‡Three participants (1 in the acupuncture group and 2 in the sham acupuncture group) did not complete the 4-week treatment due to various reasons (Details in Figure 1).

| **Table S11. Potentially clinically significant abnormalities in clinical laboratory outcomes.** Values are numbers (percentages) unless stated otherwise | | | |
| --- | --- | --- | --- |
| **Variable (unit)** | **Significance criteria** | **Acupuncture group (n=30)** | **Sham acupuncture group (n=30)** |
| ALT | ≥3×ULN | 0 (0) | 0 (0) |
| AST | ≥3×ULN | 0 (0) | 0 (0) |
| ALP | ≥2×ULN | 0 (0) | 0 (0) |
| TBIL | ≥2×ULN | 0 (0) | 0 (0) |
| BUN (mmol/L) | ≥10.71 | 0 (0) | 0 (0) |
| Cr | ≥1.5×ULN | 0 (0) | 0 (0) |
| ALT=alanine aminotransferase; AST=aspartate aminotransferase; ALP=alkaline phosphatase; TBIL=total bilirubin; BUN=blood urea nitrogen; Cr=creatinine; ULN=upper limit of normal. | | | |

| **Table S12. Abnormalities for vital signs at any time. Values are numbers (percentages) unless stated otherwise** | | | |
| --- | --- | --- | --- |
| **Variable (unit)** | **Significance criteria** | **Acupuncture group (n=30)** | Sham acupuncture group **(n=30)** |
| Pulse rate（bpm） | ≤50 and decrease ≥15 | 0 (0) | 0 (0) |
| Systolic BP (mmHg) | ≤90 and decrease ≥20 | 0 (0) | 0 (0) |
|  | ≥180 and increase ≥20 | 0 (0) | 0 (0) |
| Diastolic BP (mmHg) | ≤50 and decrease ≥15 | 0 (0) | 0 (0) |
|  | ≥105 and increase ≥15 | 0 (0) | 0 (0) |
| Respiratory rate  (breaths/min) | <10 | 0 (0) | 0 (0) |
| Weight (kg) | decrease ≥7% | 0 (0) | 0 (0) |
|  | increase ≥7% | 0 (0) | 0 (0) |
| bpm=beats per minute; BP=blood pressure. | | | |

**Figure S1. Study design.** The study for each participant will be divided into 4 periods: a 1-week screening period, a 4-week treatment period, and a 4-week follow-up period.


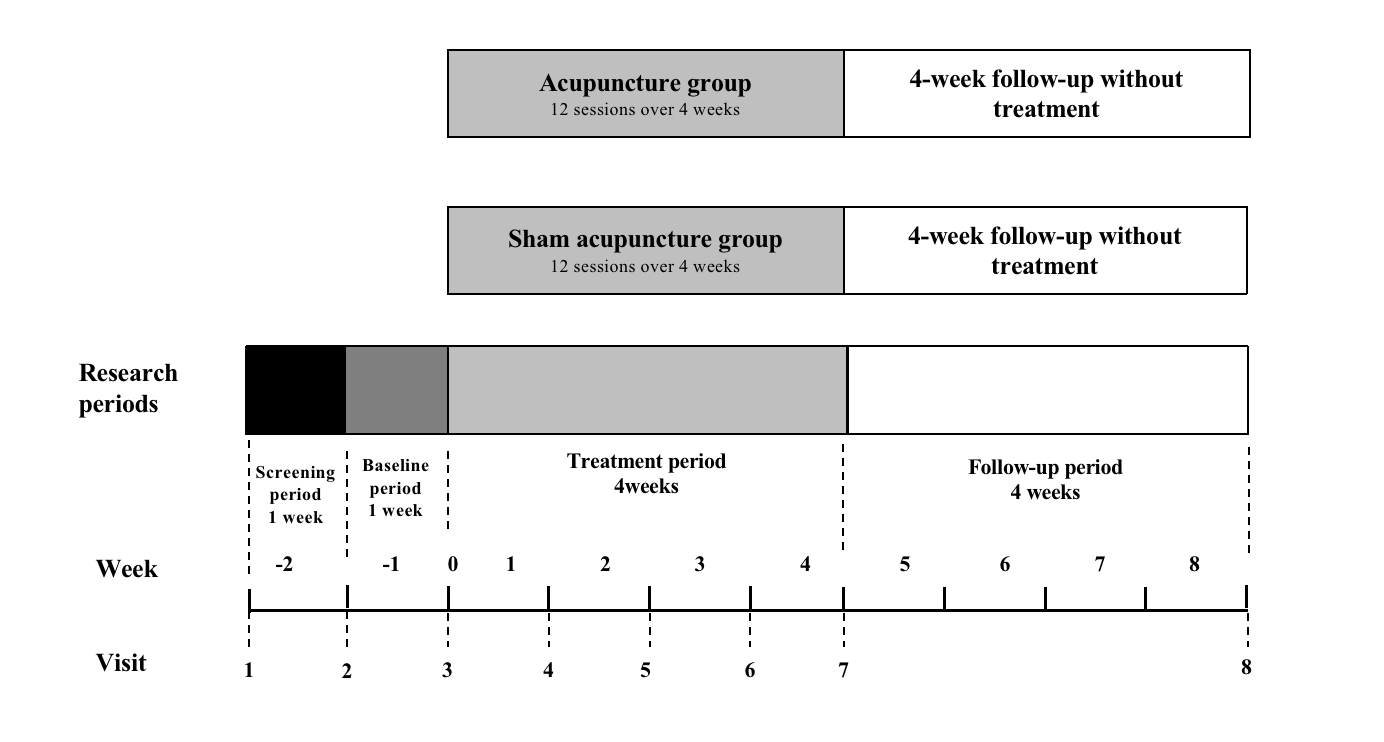


**Figure S2. Location of acupoints**


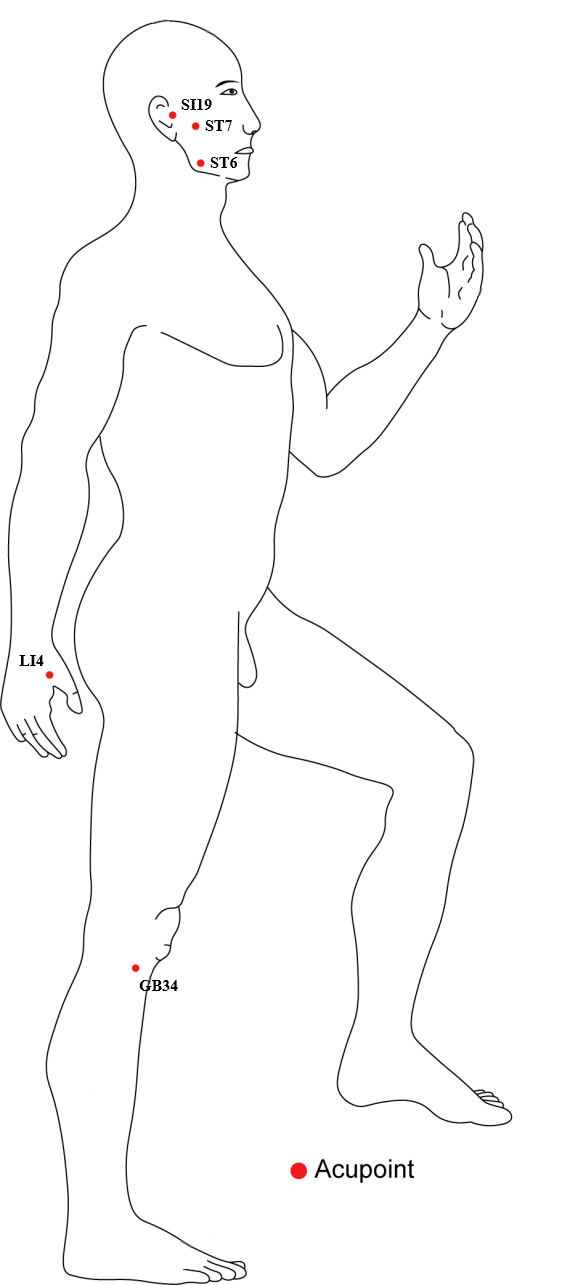


**Figure S3. Normal guide device and Park sham device**


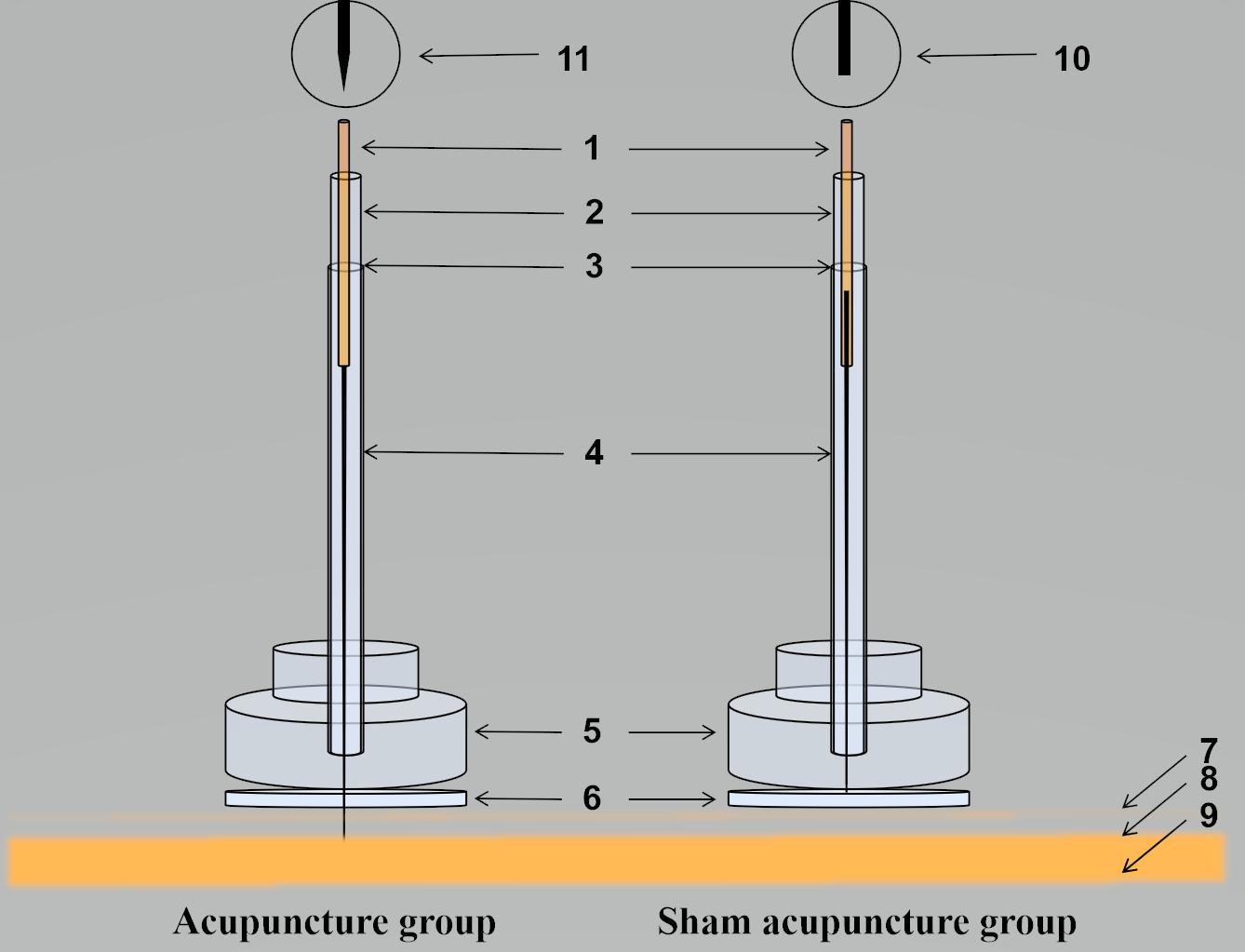


1. Needle handle, 2. Guide tube, 3. Guide o-ring, 4. Park tube, 5. Flange, 6. Adhesive pad, 7. Skin, 8. Dermis, 9. Muscle, 10. Blunt tip of sham needle, 11. Sharp tip of real needle.

**Figure S4.** **The sites of PPT measurement**


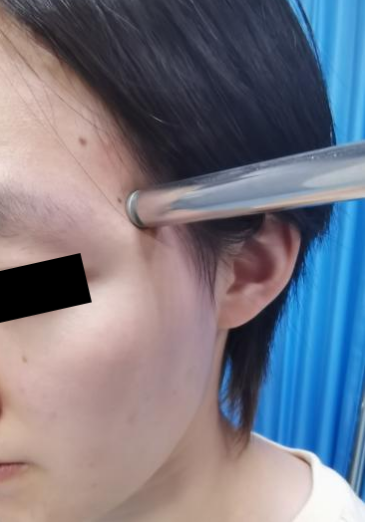
 **
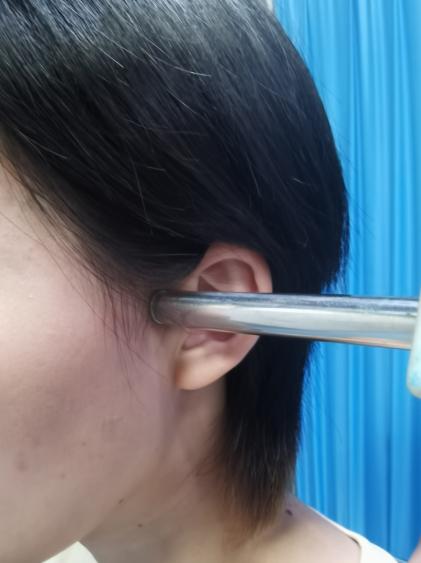

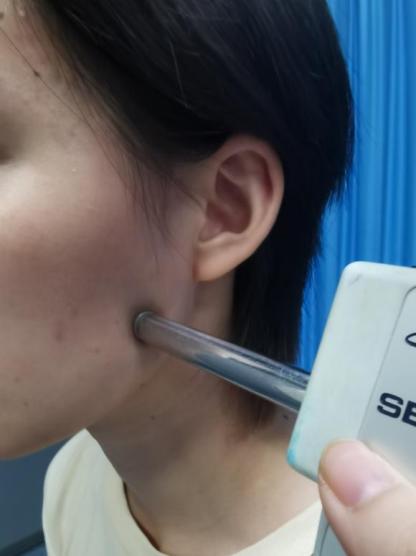
**

·Anterior temporalis muscle ·Temporomandibular joint ·Masseter muscle

**
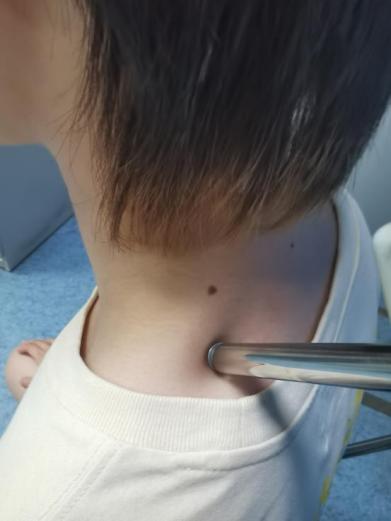

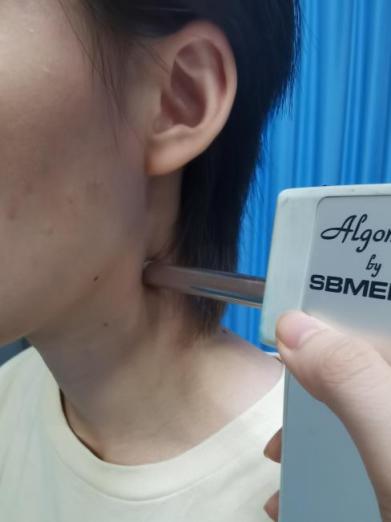
**

·Sternocleidomastoid ·Trapezius muscle

**Figure S5. The sites of sEMG measurement**

**
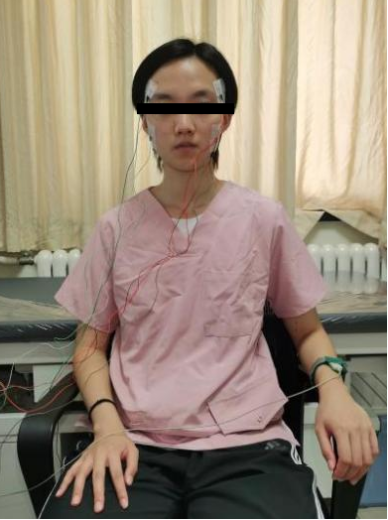

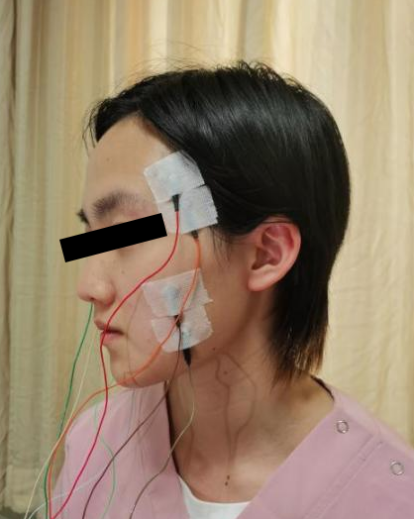

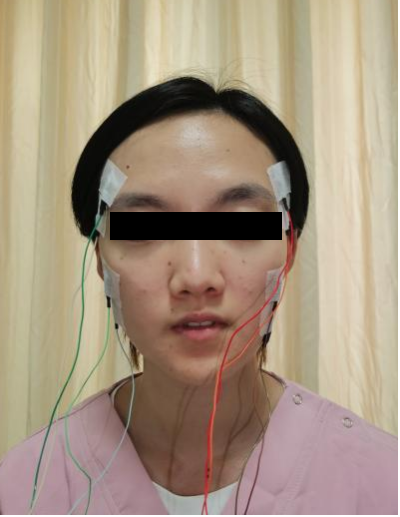
**

**Figure S6. Proportion of patients with ≥30% reduction in weakly pain intensity during treatment and follow-up periods**


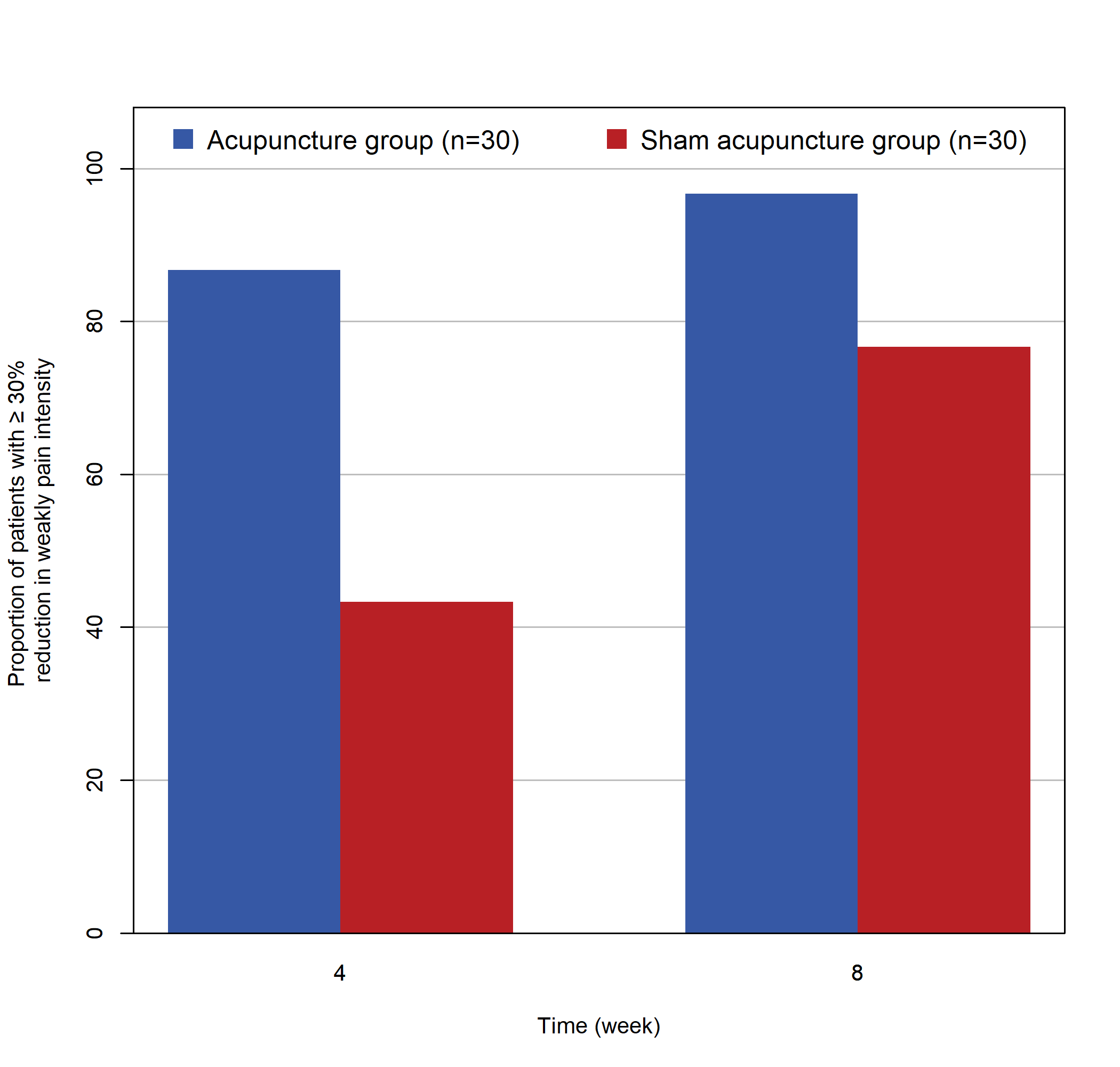


**Figure S7. Proportion of patients with ≥50% reduction in weakly pain intensity during treatment and follow-up periods**


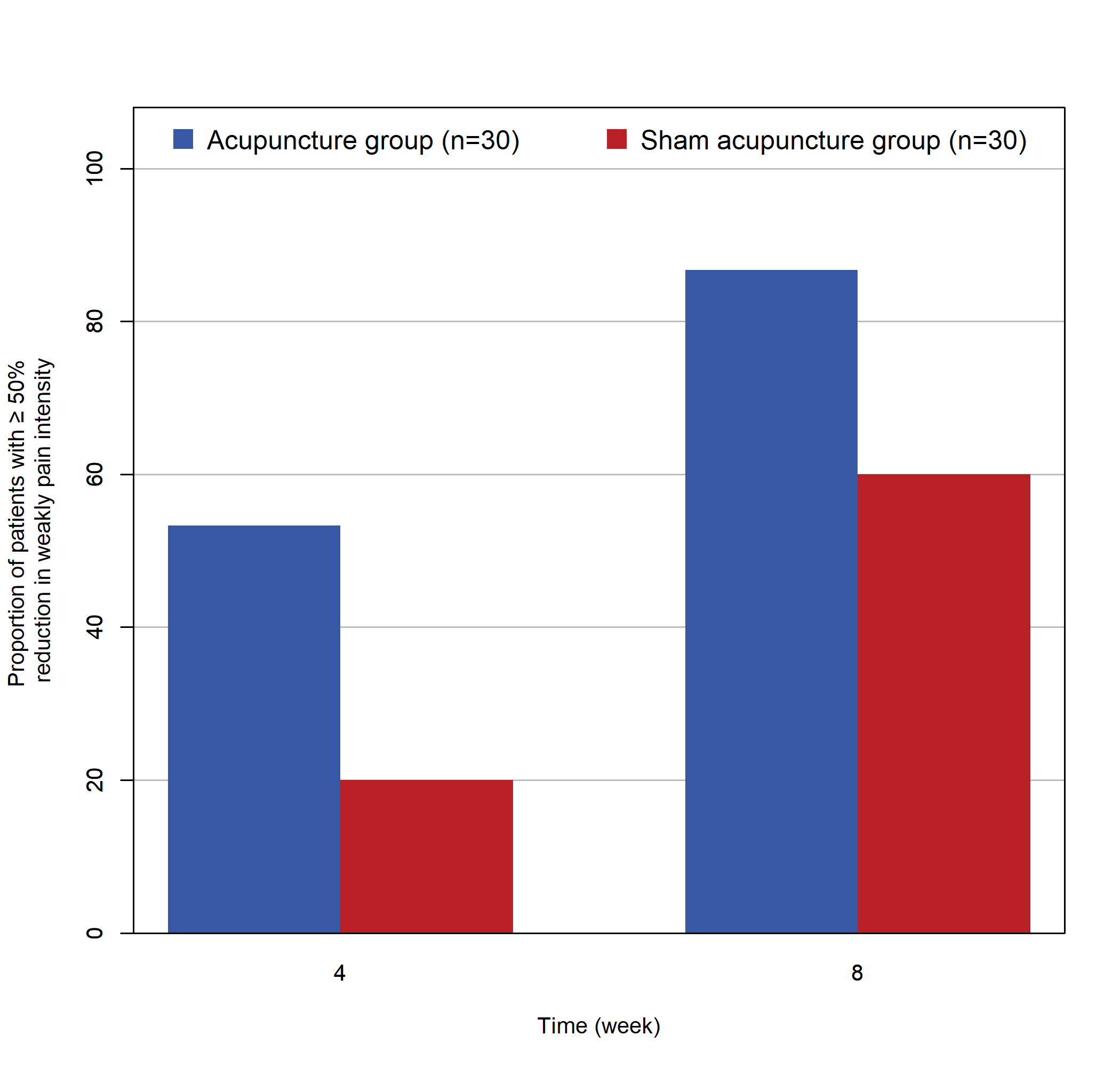

Supplement: hcae094_Supplementary_Data [file hcae094_supplementary_data.zip › hcae094_Supplementary_Data/Supplement_2-20240503.docx]
